# Engineering Bulk Photovoltaic Effect in 2D Transition Metal Dichalcogenides

**DOI:** 10.1002/advs.202514337

**Published:** 2025-10-30

**Authors:** Sikandar Aftab, Arden L. Moore

**Affiliations:** ^1^ Institute for Micromanufacturing Louisiana Tech University Ruston LA 71272 USA

**Keywords:** 2D TMDs, bulk photovoltaic effect, phase engineering, polarization effects, structural engineering, van der Waals heterostructures

## Abstract

A promising method for producing photocurrents above the Shockley–Queisser limit in non‐centrosymmetric materials is the bulk photovoltaic effect (BPVE), especially in low‐dimensional materials. 2D transition metal dichalcogenides (2D TMDs) provide a unique platform for BPVE engineering because of their strong light–matter interactions, varied electronic phases, and tunable crystal symmetry. This review systematically examines how 2D TMDs can induce or improve BPVE through phase engineering and structural modification. Phase transitions (such as 2H → 1T/1T′ and 2H → 3R) that break inversion symmetry and allow shift currents are discussed. Strain fields, van der Waals (vdWs) heterostructures, twisted bilayers, lattice distortions, and rolled morphologies are considered in the context of structural engineering, all of which modify symmetry and electronic structure to regulate BPVE. Large photocurrents can be driven by spontaneous polarization in piezoelectric and ferroelectric 2D materials, including single‐ and dual‐polarization systems. Attention is also drawn to how the directionality of the polarization field, edge contacts, and depolarization fields affect the photovoltaic response. In this comprehensive review, Design guidelines and new approaches for improving BPVE in 2D TMD systems are presented, with ramifications for energy‐harvesting and next‐generation optoelectronic devices.

## Graphical abstract

Structural and phase engineering in two‐dimensional transition metal dichalcogenides (2D TMDs), through lattice distortion, phase transitions, and polarization modulation, to induce the bulk photovoltaic effect (BPVE) for optoelectronic and energy conversion applications.

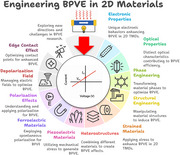

## 1 Introduction

A key component of the shift to renewable energy is photovoltaic (PV) technology, which offers an emissions‐free, scalable approach to electricity production with a significantly lower potential for global warming than fossil fuels.^[^ ^1^, ^2^, ^3^ ^]^ The market is dominated by silicon‐based PV because of its maturity, efficiency (≈26%), and established infrastructure, but newer alternatives like perovskite solar cells (PSCs) are gaining traction; tandem designs of these cells have efficiencies that are almost 34%.^[^ ^4^, ^5^ ^]^ Solar PV has inherent limitations, such as efficiency plateaus, material purity requirements, and complex manufacturing, despite being the fastest‐growing renewable source and predicted to drive capacity expansion through 2030. Next‐generation approaches, including quantum materials, tandem systems, and perovskites, are being investigated to address these issues.^[^ ^6^, ^7^, ^8^ ^]^ Mechanisms such as the bulk photovoltaic effect (BPVE) present encouraging avenues to overcome present technological constraints and propel further advancements in solar energy conversion in this dynamic environment.^[^ ^9^, ^10^, ^11^, ^12^ ^]^ A nonlinear optical phenomenon known as BPVE is seen in non‐centrosymmetric materials, where the absence of inversion symmetry permits the production of direct current (DC) under uniform illumination. BPVE operates in homogeneous, single‐phase materials without requiring complicated device architectures or external bias, in contrast to conventional PV effects that rely on heterojunctions or interfaces to separate charge carriers.^[^ ^13^, ^14^, ^15^ ^]^

When materials without inversion symmetry are exposed to uniform illumination, BPVE generates a constant DC current regardless of interfaces. The shift current, which originates from the coherent displacement of electronic charge during photoexcitation, is one of its intrinsic mechanisms that is mainly insensitive to scattering.^[^ ^16^, ^17^ ^]^ It can now be precisely calculated using first‐principles quantum mechanical techniques that assess the second‐order nonlinear optical response tensor.^[^ ^13^ ^]^ The electronic band structure, Wannier center shifts, and Berry connections of the material all have a significant impact on this response. The ballistic current, on the other hand, is more extrinsic and arises from momentum‐asymmetric carrier distributions that are dependent on phonon and exciton interactions, among other scattering processes, making numerical treatment difficult. The injection current reflects the quantum geometric properties of the bands and opens up new experimental possibilities. It is an intrinsic form of ballistic current generated under circularly polarized light. In order to optimize BPVE‐driven photocurrent, materials with customized symmetry, polarization, and quantum topology can be logically designed thanks to recent theoretical developments. In order to improve photovoltaic performance and identify realistic efficiency limits, it is now crucial to comprehend the quantitative relationships between these mechanisms—shift currents, injection currents, and polarization effects—and device efficiency.^[^ ^17^, ^18^ ^]^

BPVE provides a way to overcome the Shockley–Queisser (SQ) that limits conventional solar cell efficiencies by producing open‐circuit voltages (V_OC_) that can surpass the material's bandgap thanks to this inherent mechanism.^[^ ^19^ ^]^ A single‐junction solar cell's theoretical maximum efficiency (≈33.7% under standard AM1.5 solar illumination) is defined by the SQ limit, which was established in 1961 by William Shockley and Hans‐Joachim Queisser.^[^ ^20^, ^21^, ^22^, ^23^, ^24^ ^]^ It results from fundamental losses like thermalization (excess photon energy dissipated as heat), spectral mismatch (inability to absorb sub‐bandgap photons), and radiative recombination (loss of carriers prior to current generation).^[^ ^25^, ^26^ ^]^ These limits are predicated on perfect optics and a single p‐n junction, while real‐world silicon solar cells usually only reach ≈24% efficiency because of extra material and optical flaws.^[^ ^27^ ^]^ **Table** **1** summarizes the efficiency and key features of various solar cell technologies, while **Table** **2** highlights their respective advantages and challenges. The BPVE is one promising alternative PV mechanism to get around these limitations. In contrast to traditional devices that rely on intrinsic electric fields, BPVE happens in non‐centrosymmetric materials where asymmetric crystal structures allow shift currents to generate direct current, frequently resulting in PVs that are higher than the bandgap of the material.^[^ ^10^, ^12^ ^]^ BPVE‐based architecture might provide a workable way to get past the SQ limit and open the door to next‐generation high‐efficiency solar technologies, provided that research into cutting‐edge materials like ferroelectrics, topological insulators, and low‐dimensional systems continues.

**Table 1:** Efficiency and characteristics of various solar cell technologies.

Solar cell type	Commercial efficiency	Laboratory efficiency	Cost	Remarks
Crystalline Silicon (c‐Si)	Up to 22%^[^ ^28^ ^]^	≈26.3%^[^ ^28^ ^]^	Low–Medium	The most popular, established, and reasonably priced technology
AlGaAs–GaAs (Heterojunction)	≈26–29% (theoretical)^[^^]^	≈31.1% (simulated)^[^ ^29^ ^]^	High	High performance, expensive, usually utilized in aerospace or specialized applications
Multi‐junction (III–V)	>30% (theoretical)^[^ ^30^ ^]^	>30% (lab)^[^ ^30^ ^]^	Very High	Efficiency that breaks records; perfect for space missions and concentrated photovoltaics
GaAs Nanowires on Si	≈7.7% (lab, NW array)^[^ ^31^ ^]^	—	Potentially Lower	A high power‐to‐weight ratio that shows promise for cost reduction and integration
Organic PVs (OPV)	≈6–15%^[^ ^28^ ^]^	Up to 25%^[^ ^28^ ^]^	Low	Flexible and lightweight, but with reduced long‐term stability and effectiveness
BPVE‐based Cells	<5% (e.g., 4.8%)^[^ ^19^ ^]^	Orders of magnitude below c‐Si	—	Theoretically exceeds the SQ limit, but has low efficiency in real‐world devices right now

**Table 2:** Advantages and challenges of various solar cell technologies.

Technology	Typical efficiency [%]	Material	Bandgap [eV]	Advantages	Challenges	Refs.
Monocrystalline Silicon (c‐Si)	20–26%	Silicon	≈1.1	Long lifespan and high efficiency	High production costs	[32]
Poly‐Si, or polycrystalline silicon	15–20%	Silicon	≈1.1	less expensive than monocrystalline	Lower efficiency	[32]
Thin‐Film CdTe	13–18%	Cadmium Telluride	≈1.5	Low‐cost manufacturing, good performance in low light	Toxic material (cadmium), lower efficiency	[32]
Thin‐Film CIGS	13–21%	Cu(In,Ga)Se_2_	≈1.0–1.7	High absorption coefficient and adaptable substrate compatibility	complicated manufacturing procedures	[32]
Perovskite	20–25% (lab)	Hybrid organic–inorganic	≈1.5	Low‐cost, lightweight, and adjustable bandgap	Concerns about toxicity and stability	[33, 34]
Organic PV (OPV)	10–15% (lab)	Organic polymers/small molecules	≈1.5–2.5	Lightweight, inexpensive, and flexible	Short lifespan and low efficiency	[32]
Dye‐Sensitized (DSSC)	6–12%	Dye + Electrolyte + Semiconductor	≈1.1–2.0	Transparent, inexpensive applications	Leakage of electrolytes and low efficiency	[32]
Multi‐junction (Tandem)	30–47% (lab)	III‐V compounds, Si, Perovskites	Varies	Exceptionally high effectiveness	Extremely expensive and intricate manufacturing	[32, 35]
BPVE	<5% (lab, emerging)	Non‐centrosymmetric oxides (e.g., BiFeO_3_)	>2	Open‐circuit voltage >bandgap, no p‐n junction required.	Low current output and early‐stage research	[36, 37]

**Figure 1:**
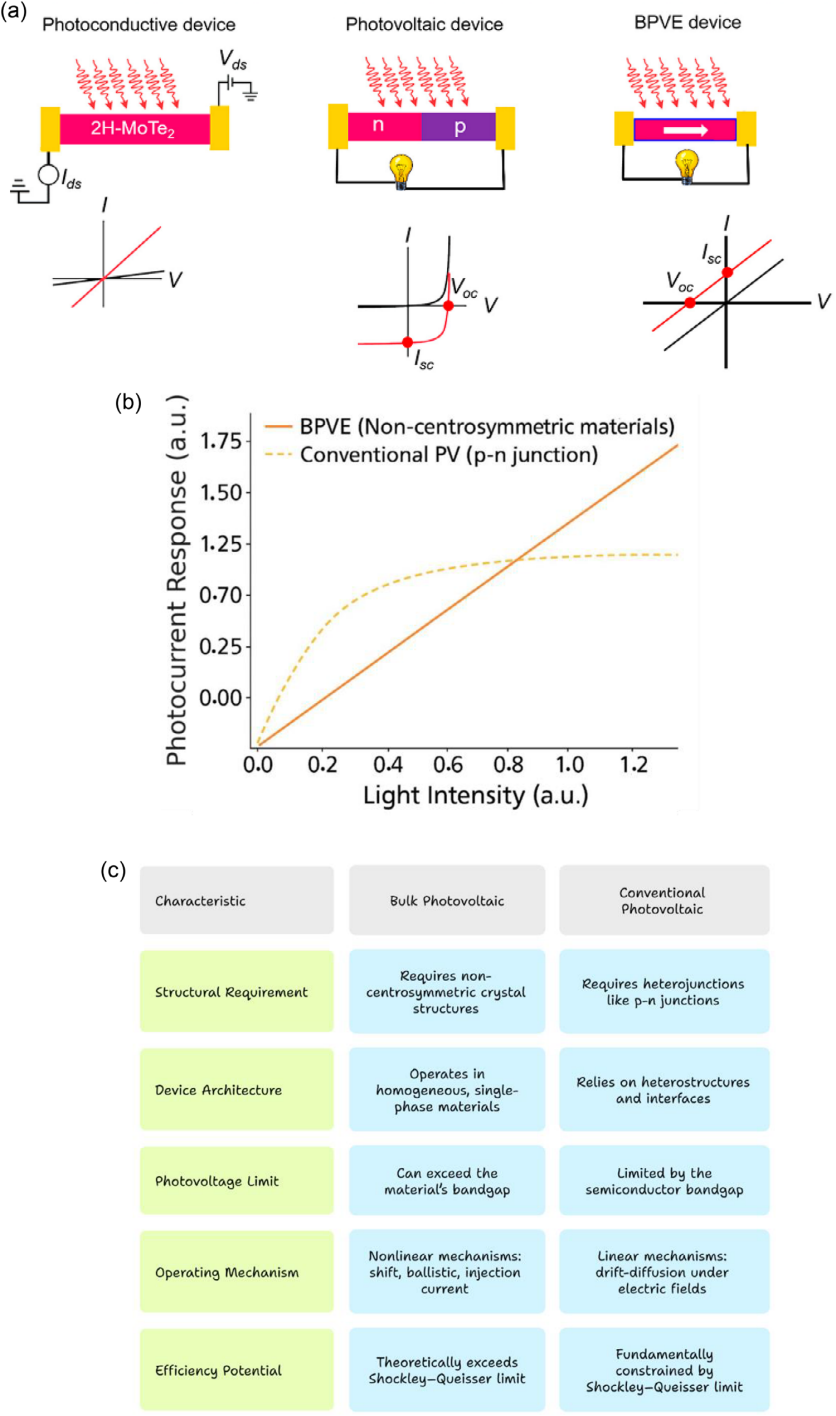
Comparison of BPVE vs. conventional PV effect. a) Three different photovoltaic devices are shown schematically: a photoconductive device, which requires an external bias to generate photocurrent because it lacks an internal electric field; a conventional photovoltaic device based on a p–n junction, where the internal electric field drives charge separation and produces distinctive *I*–*V* behavior; and a BPVE device, which uses the material's non‐centrosymmetric nature to generate photocurrent without the use of junctions or an external bias. Reproduced with permission.^[^ ^43^ ^]^ Copyright 2023, ACS. b) A comparison between BPVE and the conventional PV effect. The two mechanisms whose photocurrent response is plotted against light intensity are the conventional PV effect (dashed line) and BPVE (solid line). The conventional effect saturates at higher intensities because of p–n junction constraints, but BPVE, which comes from non‐centrosymmetric materials without junctions, responds linearly and more strongly. c) Comparison of the materials, photocurrent response, and operating principles of the BPVE and the traditional PV effect.

It is essential that BPVE have non‐centrosymmetry, a special structural requirement that allows light‐induced transitions to produce a net current.^[^ ^13^, ^38^ ^]^ Although BPVE is based on a number of physical mechanisms, the shift current is the most significant. This mechanism results from the displacement of electron charge centers in real space during photoexcitation, which produces a robust and scattering‐insensitive net photocurrent. Additional mechanisms include the injection current, which happens under circularly polarized light because of changes in the velocity of excited electrons, and ballistic current, which results from asymmetries in the momentum distribution of photoexcited carriers.^[^ ^13^, ^17^, ^39^, ^40^ ^]^ **Figure** **1**a–c contrasts the BPVE with the conventional PV effect, emphasizing the differences in charge carrier generation and transport in non‐centrosymmetric materials as opposed to p‐n junction‐based systems.^[^ ^13^, ^39^, ^41^, ^42^ ^]^

Thanks to new photophysical mechanisms and sophisticated material engineering, it is now possible to overcome the SQ limit for single‐junction PV cells. Some of the most promising approaches include using non‐centrosymmetric low‐dimensional materials, designing ultrathin solar cell architectures with improved light‐trapping capabilities, and utilizing BPVE, which enables photocurrent generation without the use of conventional p‐n junctions. In materials lacking inversion symmetry, current generation is enabled by BPVE, a linear optical process. This phenomenon has been observed to produce significant photocurrents, especially when it manifests as a shift current. For example, strain‐induced polarization and effective carrier collection in 3R‐phase MoS_2_ result in BPVE photocurrent that is more than 100 times enhanced when utilized with an edge‐contact geometry.^[^ ^37^ ^]^ The photocurrent predictions of the SQ limit are also exceeded by heterostructures made of transition metal dichalcogenides (TMDs) like MoS_2_ and WSe_2_, which combine BPVE with traditional PV mechanisms.^[^ ^37^, ^44^, ^45^ ^]^ Since low‐dimensional ferroelectrics have high joint densities of states and intrinsically asymmetric crystal structures, they also show notable shift currents.^[^ ^44^ ^]^ This includes certain 2D elemental materials.

Another crucial area for surpassing the SQ limit is the development of ultrathin solar cells. Although these thin‐film devices improve carrier extraction and use less material, they still need to overcome decreased absorption. To address this, light‐trapping strategies like transparent quasi‐random (QR) nanostructures have been used.^[^ ^46^ ^]^ For instance, waveguide mode coupling and multiresonant absorption enable ultrathin GaAs cells with QR structures to achieve 20% efficiency with just an 80 nm absorber.^[^ ^46^ ^]^ Furthermore, by improving light scattering and increasing luminescence outcoupling, the addition of textured rear mirrors to GaAs cells increases short‐circuit current (I_SC_) by 15%, up to 24.8 mA cm^−^^2^.^[^ ^46^ ^]^ The SQ threshold is further exceeded by combining low‐dimensional and low‐bandgap materials. In addition to being more environmentally stable, materials such as graphene (Gr), MoS_2_, and black phosphorus (BP) also have better light‐absorbing capabilities. Particularly, reduced graphene oxide (rGO) and graphene quantum dots (GQDs) have shown promise in raising PSC efficiency and lowering their environmental impact at the same time.^[^ ^47^ ^]^ Photocurrents in BPVE‐optimized TMDs, such as strained 3R‐MoS_2_, are several orders of magnitude higher than those in conventional ferroelectric oxides due to their improved light‐matter interaction at the nanoscale.^[^ ^44^, ^48^, ^49^ ^]^

A comparison of these crucial strategies demonstrates their unique contributions: Low‐dimensional materials enhance stability while lowering environmental impact by ≈25%; ultrathin light‐trapping architectures provide up to a 43% increase in current; and BPVE in TMDs provides a photocurrent boost of more than 100×.^[^ ^44^, ^47^, ^48^ ^]^ Examples include Gr and MoS_2_ for eco‐friendly improvements, GaAs with QR structures for ultrathin cells, and 3R‐MoS_2_ and WSe_2_ for BPVE. Theoretically, by introducing mechanisms that circumvent common restrictions, BPVE‐based devices challenge the limitations of the SQ limit. These devices use ultrathin architectures to reduce carrier recombination, absorb sub‐bandgap photons through shift current generation, and eliminate thermalization losses by directly harvesting hot carriers.^[^ ^44^, ^46^, ^50^ ^]^ In conclusion, it is becoming increasingly possible to surpass the S–Q limit by combining BPVE‐enabled materials, advanced nanostructured architectures, and ecologically friendly low‐dimensional materials. These developments pave the way for next‐generation PV technologies that promise much higher efficiencies and lower costs for the future of renewable energy.

Because 2D TMDs have tunable crystal symmetry, diverse electronic phases, and strong light–matter interactions, they offer a unique platform for BPVE engineering. The methods by which 2D TMDs can induce or enhance BPVE through phase engineering and structural modification are systematically examined in this review. We discuss phase transitions that enable shift currents and break inversion symmetry, such as 2H → 1T/1T′ and 2H → 3R. In the context of structural engineering, we consider strain fields, van der Waals (vdWs) heterostructures, twisted bilayers, lattice distortions, and rolled morphologies, all of which modify symmetry and electronic structure to control BPVE. In piezoelectric and ferroelectric 2D materials, including single and dual polarization systems, spontaneous polarization can drive large photocurrents. We also highlight the effects of edge contacts, depolarization fields, and polarization field directionality on PV response. This comprehensive review includes implications for energy‐harvesting and next‐generation optoelectronic devices, as well as design guidelines and novel methods for enhancing BPVE in 2D TMD systems. **Table** **3** outlines the potential benefits and major obstacles of using low‐dimensional materials for BPVE, emphasizing their unique electronic properties, robust light‐matter interactions, and persistent issues related to stability, scalability, and device integration. In our review, we focus on BPVE in 2D TMDs, which can surpass the S–Q limit. Unlike theory‐ or PV‐focused reviews,^[^ ^13^, ^51^, ^52^ ^]^ we emphasize mechanisms such as shift currents, phase engineering, structural modulation, and polarization effects, outlining strategies to enhance photocurrents and guide next‐generation optoelectronic and energy‐harvesting devices.

**Table 3:** Potential and challenges of low‐dimensional‐based materials for BPVE.

Material system	Mechanism for BPVE	Crystal structure/Symmetry	Advantages	Challenges	Refs.
Single‐layer MoS_2_	Shift current	Prismatic trigonal (2H phase); no inversion symmetry	Scalable, high absorption, direct bandgap (≈1.8 eV)	Exciton binding energy is high and PCE is low.	[53]
WSe_2_ monolayer	Injection current and shift current	Non‐centrosymmetric, hexagonal	High spin‐orbit coupling and effects of valley polarization	Contact resistance and uniformity of fabrication	[54]
The Janus MoSSe	The injection current and shift caused by the broken mirror symmetry	The asymmetric vertical structure known as D_3_h symmetry	Integrated electric field, improved BPVE	Complexity of synthesis and stability in the ambient	[55]
MoTe_2_ (phase 1T′)	BPVE in topology using Berry curvature	Non‐centrosymmetric and orthorhombic (1T′)	Weyl semimetal characteristics of type‐II, high shift current	1T′ phase metastability and synthesis difficulty	[56]
Heterostructures (such as WSe/MoS_2_)	Current shift between layers	The heterostructures of vdWs	Improved charge separation, type‐II interfaces, and adjustable band alignment	Interface flaws and misaligned lattice	[57]
Doped TMDs, such as MoS_2_ doped	Band engineering creates a higher shift current.	2H phase modification	Customized electrical characteristics and adjustable symmetry	Defect formation and dopant control	[58]
Ferroelectric TMDs, or hybrids based on CuInP_2_S_6_ (CIPS)	BPVE Ferroelectric	Non‐centrosymmetric, layered	Switchable current direction and built‐in polarization	TMD integration and limited mobility	[59]
3R‐MoS_2_ with Bi EC	BPVE via edge contact (EC), strain, in‐plane polarization	3R phase, edge‐contacted	BPVE enhancement, constructive coupling with conventional PV, high photocurrent	Scalability, contact stability	[60]
3R‐MoS_2_/WSe_2_ heterojunction	BPVE + conventional PV	3R‐MoS_2_/WSe_2_ stack	Enhanced photocurrent, synergy of effects	Interface quality, strain control	[60]
2D interlayer‐sliding ferroelectrics	Non‐synchronous BPVE, unswitchable in‐plane BPVE	2D, layered, ferroelectric	New physics, robust in‐plane effect	Limited tunability, device integration	[61]
MoS_2_, WS_2_, WSe_2_, MoSe_2_, etc. (general TMDs)	BPVE in non‐centrosymmetric structures	MX_2_, layered, various polytypes (2H, 1T, 3R)	Tunable bandgap, strong light‐matter interaction	Low efficiency in some configurations, strain sensitivity	[60, 62]
WTe_2_, 1T′‐phase	Realization of fractional‐layer TMDs (WTe_2_)	STM tip manipulation, fractional‐layer	Controllable removal of Te atoms, atomic reconstruction, unidirectional charge density redistribution	Complex fabrication, limited scalability	[63]
(CrTaVHfZr)S_2_ (1T), (CrNbVTiZr)S_2_ (1T), (MoNbTaVTi)S_2_ (2H)	Activating the Basal Plane of 2D TMDs by Alloying	High‐entropy alloying, 2H/1T phase control	Creation of catalytically active basal planes, high stability, novel HEA TMDs	Synthesis complexity, phase control	[64]
With bi‐edge contacts, 3R‐MoS_2_	In‐plane polarization is activated and symmetry is broken by the Bi strain	Non‐centrosymmetric stacking of 3R (ABC)	>100 × BPVE, strong Bi adhesion, better light access via lateral geometry	Top contacts prevent polarization; precise fabrication and strain/contact management are necessary	[48]
The stack of Gr and α‐In_2_Se_3_ (α‐In_2_Se_3_: 2D ferroelectric, ≈1.3 eV)	FPVE is driven by the depolarization field; current ↑ as α‐In_2_Se_3_ thickness ↑	Gr: semimetal; α‐In_2_Se_3_: out‐of‐plane ferroelectric	Ideal bandgap, thin, stable; no p‐n junction;	Low effectiveness; requires exact thickness and interface; the effect is diminished by screening	[65]
VdW heterostructures (e.g., MoS_2_‐based)	In‐plane and out‐of‐plane polarizations increase charge transfer and BPVE	Polarization results from interface symmetry breaking	Extremely quick reaction; high effectiveness; adjustable through interface design	Complex fabrication, precise interface control, and unproven scalability	[66]
NbOI_2_/MoSe_2_ vdW heterojunction	BPVE is driven by in‐plane polarization in NbOI_2_; ultrafast charge transfer	High absorption TMD for MoSe_2_; non‐centrosymmetric ferroelectric for NbOI_2_	Polarization‐sensitive, quick, self‐powered, and highly responsive	Single ferroelectrics perform poorly; exact interface and domain control are required	[67]
3R‐WS_2_	Switchable in‐plane polarization enables nonvolatile BPVE photocurrent	Non‐centrosymmetric 3R‐stacked WS_2_ that facilitates spontaneous polarization	Perfect for neuromorphic vision because it is rewriteable, self‐powered, and nonvolatile	Requires precise domain control; scalability and endurance require validation	[68]
2H‐MoS_2_ monolayer (noncentrosymmetric TMD)	Piezoelectric polarization is induced by in‐plane strain (≈0.2%), which increases photocurrent	Noncentrosymmetric and hexagonal, it facilitates in‐plane piezoelectricity	Ultrathin, flexible, and strain‐tunable	Low absolute current, complex strain‐photo coupling, and strain uniformity	[69]
Unmatched or twisted vdW heterostructures (WSe_2_/BP)	Shift current is driven by in‐plane polarization from symmetry‐breaking at the interface	In‐plane inversion symmetry is broken by the interface, but layers may still be centrosymmetric	Anisotropic, adjustable, and extremely quick response; no p‐n junction is required	Photocurrent is restricted to the polarization direction and requires exact stacking	[70]
Nanoflakes of 2H‐ and 1T′‐MoTe_2_ on stretchable materials	Strong BPVE is made possible by the strain‐induced phase transition, which breaks inversion symmetry	1T′: distorted octahedral (semi‐metallic)	self‐powered operation, adjustable bandgap	Needs precise strain control; 1T′ phase may limit some optoelectronic performance; scalability concerns	[71]
3R‐MoS_2_ (non‐centrosymmetric, rhombohedral/trigonal phase, ABC stacking)	Photocarriers are separated by strain‐induced in‐plane piezoelectric polarization, producing a massive PV response	Inversion symmetry is broken by trigonal (ABC stacking), allowing for piezoelectric and PV effects	High piezoelectric constants, scalable, effective carrier separation, and enormous photocurrent/voltage	Requires precise strain control, interface/device engineering, and limited effects in ultrathin films	[72]
WS_2_ nanotubes (rolled 2D layers)	Curvature disrupts symmetry, resulting in BPVE	Non‐centrosymmetric, polar structure.	High photocurrent, no p‐n junction, miniaturized, polarization‐sensitive	Hard to scale, uniform synthesis is difficult, and efficiency is lower than p‐n cells	[73]
Distorted MoTe_2_ (2H to 1T′ phase transition)	Strain breaks inversion symmetry, resulting in BPVE with in‐plane polarity	1T′: distorted, non‐centrosymmetric; 2H: symmetric	self‐powered, high responsive	semi‐metallic, restricted scalability, and precise strain control	[43]
Heterostructures of twisted BP	Twist breaks symmetry, allowing interfacial BPVE and prohibited infrared emission	Twist breaks in‐plane symmetry	IR detection powered by self‐power, twist/thickness adjustable, bright mid‐IR light	Control of scalability, bubble removal, and twist/strain	[74]

## 2 Unique Advantages of BPVE in 2D TMDs Materials

### 2.1 Electronic Properties

**Figure 2:**
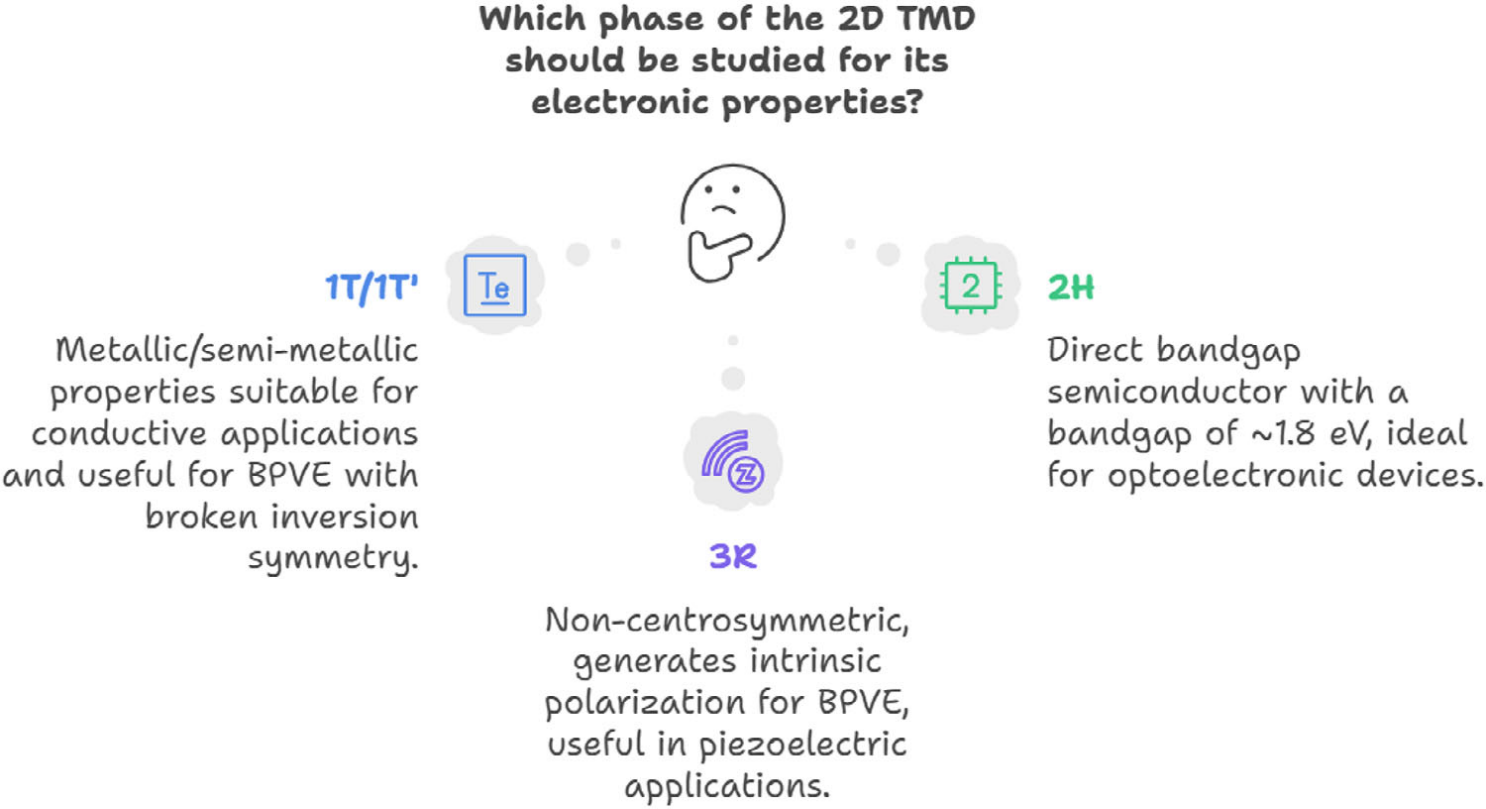
Phase‐dependent properties of 2D TMDs.

BPVE is made possible by the electronic and optical characteristics of 2D TMDs, such as MoS_2_, WS_2_, MoSe_2_, and WSe_2_, especially in non‐centrosymmetric phases like the 3R phase.^[^ ^48^ ^]^ The performance of BPVE is greatly influenced by the electronic characteristics of the material. The electronic behavior of TMDs is influenced by their phase‐dependent band structures; the 1T/1T′ phases show metallic or semi‐metallic characteristics, whereas the 2H phase functions as a direct bandgap semiconductor.^[^ ^75^ ^]^ Being non‐centrosymmetric, the 3R phase is crucial for BPVE because it produces intrinsic polarization fields that propel photocurrents in the absence of an outside bias. Through the use of defects like chalcogen vacancies, bandgap engineering can reduce the 2H phase's bandgap from ≈1.8 to below 0.8 eV, allowing for adjustable optoelectronic responses.^[^ ^75^ ^]^ The light absorption characteristics of W‐based TMDs, such as WS_2_, are affected by their wider bandgaps compared to their Mo‐based counterparts.^[^ ^75^ ^]^ Furthermore, the energy difference between phases (2H, 1T, 1T′) is reduced by defect‐induced modifications, especially chalcogen vacancies, which destabilize the 2H structure and introduce mid‐gap states that improve the efficiency of charge separation in PV devices.^[^ ^75^, ^76^ ^]^ BPVE performance is further optimized by strain and contact engineering, which improves photocurrent and PVs by applying tensile strain to 3R‐MoS_2_, increasing its BPVE coefficient by up to 4.93 × 10^−1^ V^−1^.^[^ ^48^ ^]^ BPVE output is increased, and carrier loss is reduced by using low‐resistance electrical contacts, such as semi‐metallic Bi electrodes (**Figure** **2**).

### 2.2 Optical Properties

**Figure 3:**
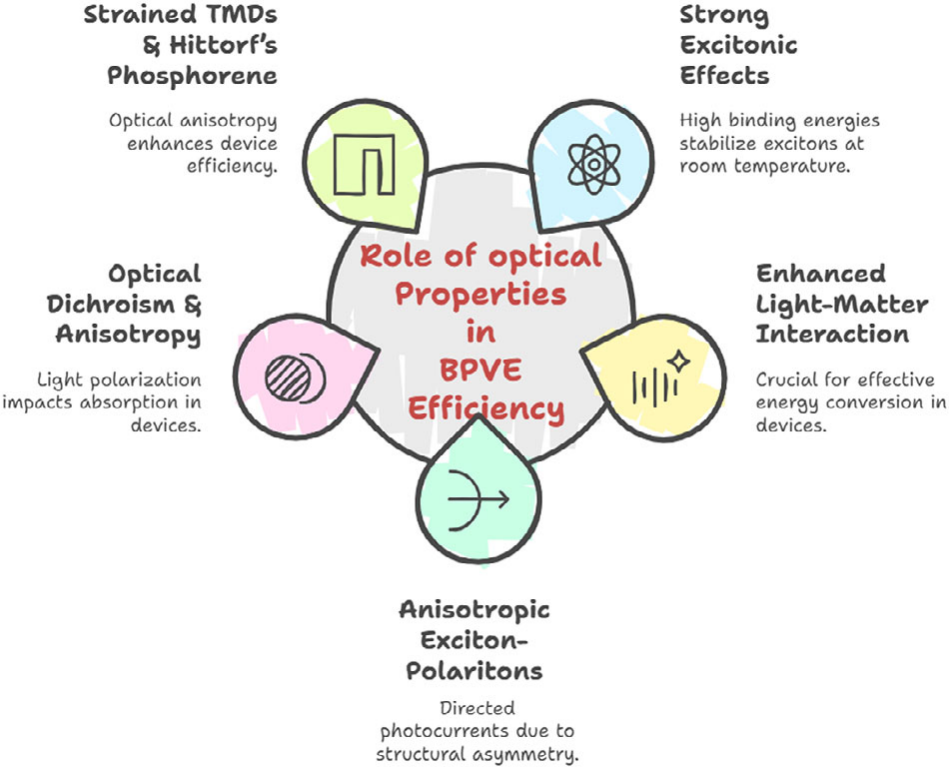
Optical properties of 2D TMDs.

In BPVE, the optical characteristics of TMDs are also critically important. Owing to high exciton binding energies (300–500 meV in MoS_2_ and WS_2_), these materials exhibit strong excitonic effects and enable stable exciton formation at room temperature.^[^ ^76^, ^77^ ^]^ This improves the interactions between light and matter, which are important for PV and polaritonic applications.^[^ ^77^, ^78^ ^]^ For instance, monolayer WSe_2_ displays anisotropic exciton‐polaritons as a result of in‐plane structural asymmetry, enabling the generation of directed photocurrents.^[^ ^77^, ^79^ ^]^ Moreover, TMDs show optical dichroism and anisotropy, in which the polarization of the light affects the absorption.^[^ ^79^ ^]^ This characteristic can be used to create polarization‐sensitive BPVE devices and is observed in materials such as strained TMDs and Hittorf's phosphorene (**Figure** **3**).

## 3 Centrosymmetric vs Non‐Centrosymmetric Systems in 2D TMDs

**Figure 4:**
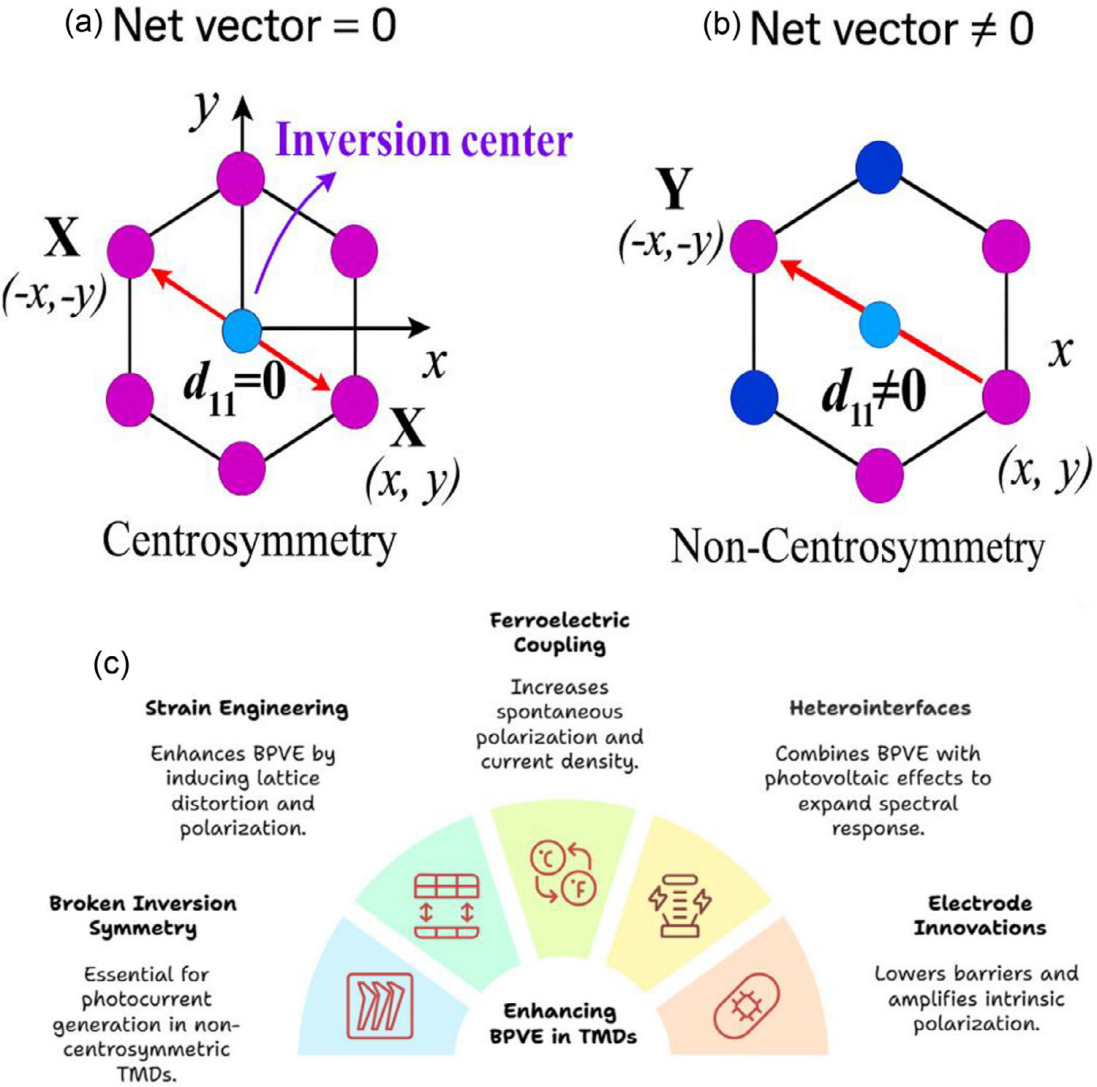
Differentiation of a) centrosymmetry and b) non‐centrosymmetry crystal structure in 2D TMDs. c) Optimization of BPVE in 2D TMDs.

In 2D TMDs, BPVE displays different behaviors depending on whether the system is centrosymmetric or non‐centrosymmetric, with symmetry serving as a key factor in determining its efficiency (**Figure** **4**a,b). Through crystal asymmetry‐driven carrier separation, where non‐centrosymmetric materials such as 3R‐MoS_2_ exhibit direction‐dependent transition probabilities, allowing electron–hole pair separation along polarization axes without the need for p‐n junctions, BPVE requires broken inversion symmetry to generate photocurrents.^[^ ^80^ ^]^ This mechanism is dominated by shift currents. In contrast, centrosymmetric polytypes, such as 2H‐MoS_2_, lack this intrinsic property due to symmetric electron transitions, the absence of intrinsic polarization, and a dependence on drift‐diffusion mechanisms.^[^ ^61^, ^80^, ^81^ ^]^ This means that net photocurrent generation cannot occur unless external symmetry‐breaking methods, such as ferroelectric coupling, edge contacts, strain engineering, or heterointerfaces, are used. In contrast to centrosymmetric materials, which require external modifications to achieve even limited PV responses, strategies such as interface strain induced by Bi semimetal electrodes can enhance BPVE by lowering Schottky barriers and amplifying intrinsic polarization, resulting in significantly higher I_SC_, V_OC_, and quantum efficiencies.^[^ ^48^, ^80^ ^]^ Combining BPVE with traditional PV effects may overcome the SQ limit, according to recent developments, especially in 3R‐MoS_2_/WSe_2_ heterojunctions.^[^ ^48^, ^80^ ^]^ This suggests that symmetry‐engineered 2D TMDs could be important contenders for next‐generation optoelectronics. The process of optimizing BPVE in 2D TMDs is depicted in Figure 4c, which also highlights the contributions of heterointerfaces, edge contacts, strain engineering, and inversion symmetrical breaking to improved photocurrent generation. To increase efficiency, a number of BPVE optimization techniques have been proposed. To balance material stability and efficiency, band structures and carrier densities can be precisely modulated by controlling defect concentrations, especially tuning chalcogen vacancy levels between 3.13% and 21.88%.^[^ ^75^, ^76^ ^]^ Furthermore, band inversions up to 0.78 eV are introduced by strong spin‐orbit coupling in W‐based TMDs, such as WSe_2_, allowing spin‐polarized photocurrents, which are advantageous for spintronic‐PV hybrid applications.^[^ ^75^ ^]^ These observations demonstrate how engineered TMDs can be used to improve BPVE performance in next‐generation optoelectronic devices by utilizing their special electronic and optical characteristics.^[^ ^48^, ^75^, ^79^ ^]^

## 4 Phase Engineering in 2D Materials for BPVE

Phase transitions in 2D TMDs, such as 2H → 1T/1T' and 2H → 3R, result in significant changes in material properties that enable a range of applications in energy storage, electronics, and catalysis (**Table** **4**).^[^ ^82^, ^83^, ^84^, ^85^ ^]^ In the next sections that follow, we go into more detail about two of the most promising types of transitions.

**Table 4:** The structural phases of 2D TMDs and their role in phase engineering.

Phase	Symmetry	Coordination	Properties	Key features	Role in phase engineering
2H	Hexagonal	Trigonal prismatic	Semiconducting	PL active; ABA stacking; most stable.	Ideal for electronic and optoelectronic devices, this phase is the beginning of many transitions.
3R	Rhombohedral	Trigonal prismatic	Semiconducting	Breaks inversion symmetry; metastable; ABC stacking.	Stacked to accommodate valleytronics and nonlinear optics (like SHG).
1H	Hexagonal	Trigonal prismatic	Semiconducting	2H monolayer form; vertical alignment of chalcogens.	Targeted by exfoliation or CVD; pertinent to monolayer electronics.
1T	Tetragonal	Octahedral	Metallic	Metastable; frequently unstable when standing alone.	Chemically induced for catalysis and electronics, precursor to 1T′/1Td.
1T′	Monoclinic	Distorted octahedral	Metallic/Topological	High conductivity; zigzag metal chains.	Utilized in HER catalysis and topological quantum devices, it is accomplished by doping and strain.
1Td	Orthorhombic	Distorted octahedral	Semimetallic/Topological	Low‐T phase; distinct stacking.	Stabilized by heat or strain; utilized in Weyl semimetal studies.
2Hd	Distorted hex.	Distorted trig. prismatic	Semimetallic/Transitional	A structure that changes under field or strain.	A crucial transitional phase in straintronics and dynamic switching.
1T″	Triclinic	Distorted octahedral	Metallic/Metastable	Shows off superstructures, like tetramers.	Doping/strain produces a rare phase that aids in the investigation of charge/lattice instabilities.
CDW (Charge Density Wave)	Modulated	Variable	Variable (often insulating)	Lattice/electron modulations that occur periodically.	Correlated electron systems are investigated; tunable via T, strain, and doping.

### 4.1 2H → 1T/1T' Phase Transition

**Figure 5:**
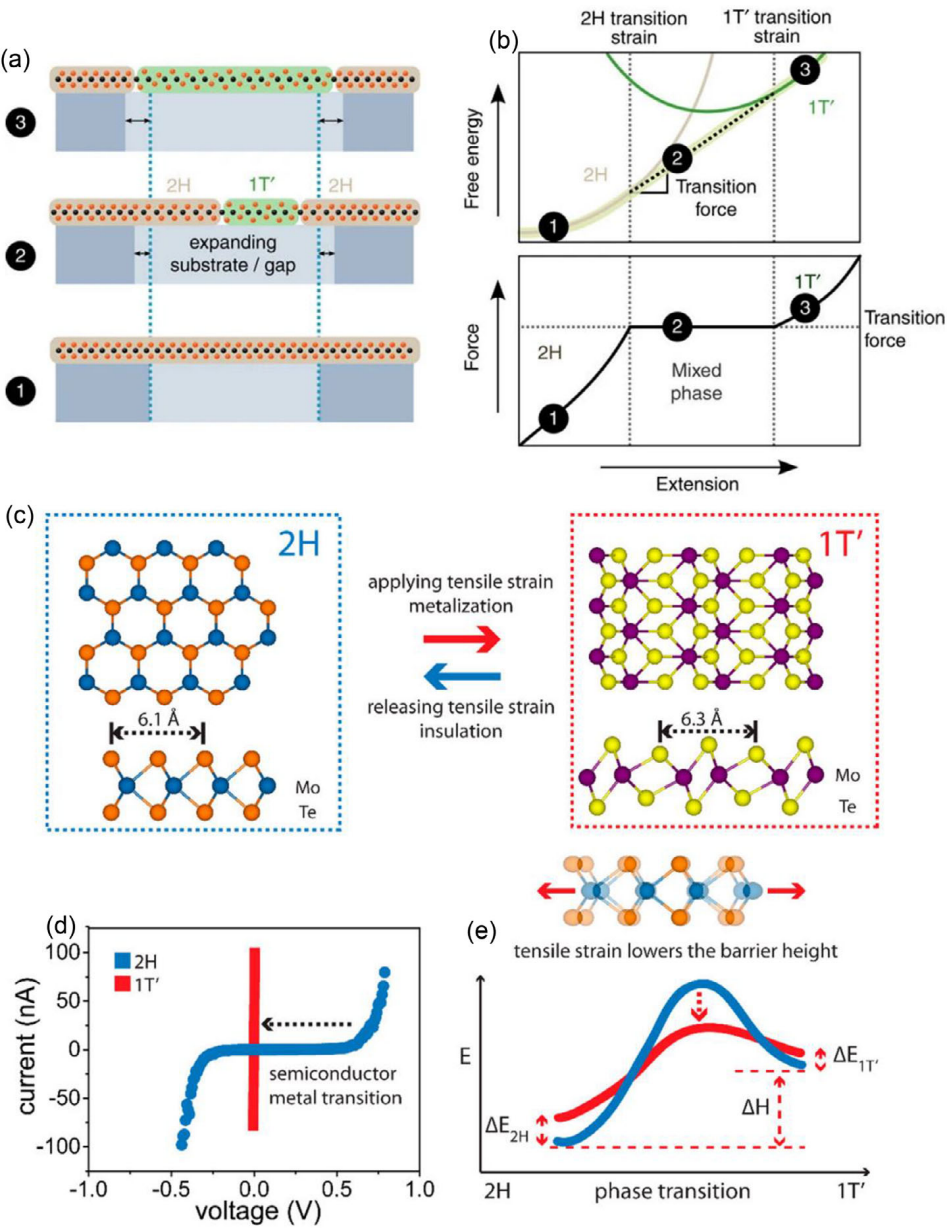
a) The TMD monolayer can be expanded by tensile mechanical deformation in a region that may be locally slide or freely suspended over a low‐friction substrate. b) The system changes throughout the 2H/1T′ Helmholtz free energy landscape during this process. (a‐b) Reproduced under the terms of the CC‐BY 4.0 license.^[^ ^89^ ^]^ Copyright 2014, Duerloo et al. c) The polymorphic phase of MoTe_2_ can be controlled by in‐plane tensile strain, as demonstrated by the atomic structures of 2H and 1T′ MoTe_2_. d) The 2H to 1T′ phase transition and the semiconductor‐to‐metal transition happen at the same time, as shown by variations in the *I*–*V* curves. e) The strain‐modulated phase transition barrier schematic shows how tensile strain lowers the activation energy and, consequently, the phase transition temperature. (c–e) Reproduced with permission.^[^ ^90^ ^]^ Copyright 2016, ACS.

In a 2H lattice, the energetically demanding nucleation of 1T/1T' phases usually starts at phase boundaries or defect sites.^[^ ^86^ ^]^ Compared to armchair (AC) boundaries, zigzag (ZZ) boundaries spread more slowly, resulting in rounded domains during shrinkage and triangular or hexagonal 1T domains during growth.^[^ ^86^, ^87^ ^]^ This change from semiconducting 2H to metallic 1T/1T' phases makes low‐power electronics possible.^[^ ^84^, ^88^ ^]^ Under strain or electrochemical doping, MoTe_2_ undergoes reversible 2H ↔ 1T' transitions, and Raman spectroscopy verifies metallic behavior in the 1T' phase. This transition, which causes a shift from semiconductor to metal, can be triggered by a tensile strain of 0.2–3%.^[^ ^84^, ^88^ ^]^ Duerloo et al.^[^ ^89^ ^]^ discovered that mechanical deformations in monolayer materials can alter the thermodynamic stability of metallic and semiconducting crystal structures. The overall idea of mixed‐phase regimes as a monolayer experiences increasing tensile strain (such as hydrostatic pressure, uniaxial load, or uniaxial strain) is depicted in **Figure** **5**a. The 2H phase undergoes elastic deformation in step 1 without undergoing a phase transition. The coexistence regime, in which both phases maintain mechanical equilibrium, is created when the lowest free‐energy path creates a common tangent between the 2H and 1T′ energy surfaces following a critical strain in step 2. When a rigid AFM probe controls the extension, this area shows up as a plateau in the applied force curve (Figure 5b). The system fully adopts the lowest energy 1T′ phase at the strain level of step 3, marking the end of the mechanically induced phase transition. The transformation of MoTe_2_ under uniaxial conditions at 300 K is found to require a range of 0.3–3% tensile strain.

Another study by Song et al.^[^ ^90^ ^]^ shows a strain‐engineered semiconductor‐metal transition at room temperature in thin‐film MoTe_2_. They were able to reduce the 2H–1T′ phase transition temperature of MoTe_2_ to room temperature by putting it under a 0.2% tensile strain. The atomic ball‐and‐stick structures of the 2H and 1T′ phases of MoTe_2_ are depicted in Figure 5c. Te atoms in the 2H phase align to preserve hexagonal symmetry in the top view, whereas in the 1T′ phase, distorted coordination causes the Te atoms to be offset, increasing the in‐plane lattice constant by ≈3% (6.3 vs 6.1 Å). The semi‐metallic 1T′ phase has a higher low‐bias conductivity than the semiconducting 2H phase, which has a bandgap of 1 eV, as illustrated in Figure 5d.^[^ ^91^, ^92^ ^]^ A decrease in the activation energy barrier during the transition state and strain‐driven modulation of the cohesive energy of each phase are responsible for the tensile‐strain‐induced phase transition of 2H‐MoTe_2_, which is schematically depicted in Figure 5e. It is anticipated that other TMDs will benefit from the strain modulation of the phase transition temperature that has been demonstrated, allowing for the creation of 2D electronics that utilize TMD polymorphism in addition to established materials.

### 4.2 2H → 3R Phase Transition

**Figure 6:**
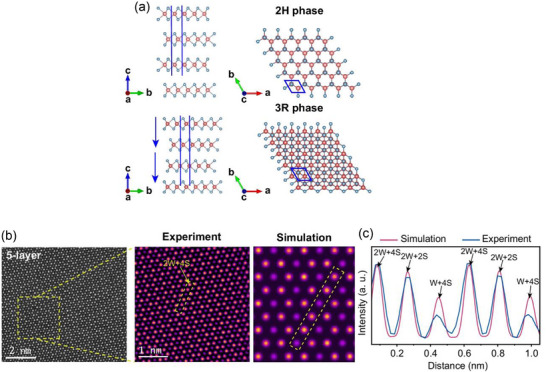
a) Schematics of the 2H and 3R WS_2_ crystals in top and side views along various axes; atoms of sulfur and tungsten are represented by the blue and red spheres, respectively. The unit cells are highlighted by blue boxes, and the spontaneous polarization direction is indicated by blue arrows. b) Five‐layer 3R‐WS_2_ atomic‐resolution ADF‐STEM images and related simulations. c) Simulated and experimental intensity line profiles for the area in (b) that is indicated by the yellow dashed rectangle. (a–c) Reproduced under the terms of the CC‐BY 4.0 license.^[^ ^68^ ^]^ Copyright 2024, Gong et al.

The WS_2_ polytype 3R‐WS_2_ (*space group: R3m*) is made up of W−S triangular prism layers that are covalently bonded together (**Figure** **6**a). The ABC‐type stacking order of 3R‐WS_2_ results in non‐centrosymmetric triangular symmetry from bilayer to bulk, which induces spontaneous polarization (sliding ferroelectricity) along the out‐of‐plane directions.^[^ ^93^ ^]^ The 2H phase of WS_2_ (*space group: P6_3_/mmc*) does not show out‐of‐plane spontaneous polarization since the even‐layer system maintains inversion symmetry and the odd‐layer system maintains mirror symmetry. Gong et al.^[^ ^68^ ^]^ used the chemical vapor transport (CVT) method to successfully synthesize 3R‐WS_2_ crystals. Each hexagon has weak spots in the middle that set 3R‐WS_2_ apart from the 2H phase. The five‐layer 3R‐WS_2_ intensity line distribution shows a rhombic crystal structure with alternating contrasts of light and dark along the lattice direction tilted at 60°. This observation is consistent with the 3R‐WS_2_ crystal structure shown in Figure 6b. The atoms in the yellow dotted line box are arranged as follows, according to the atomic structure of 3R‐WS_2_: 2W + 4S, 2W + 2S, W + 4S, 2W + 4S, 2W + 2S, and W + 4S. This arrangement closely resembles the simulated STEM images, as seen in Figure 6c. The structural symmetry of WS_2_ crystals can be effectively examined using second harmonic generation (SHG) microscopy. It is anticipated that the strong intrinsic piezoelectric field created when the inversion symmetry of 3R‐WS_2_ is broken will strengthen the SHG signal.^[^ ^68^ ^]^

## 5 Structural Engineering in 2D TMDs to Induce BPVE

### 5.1 Twisted 2D TMDs

**Figure 7:**
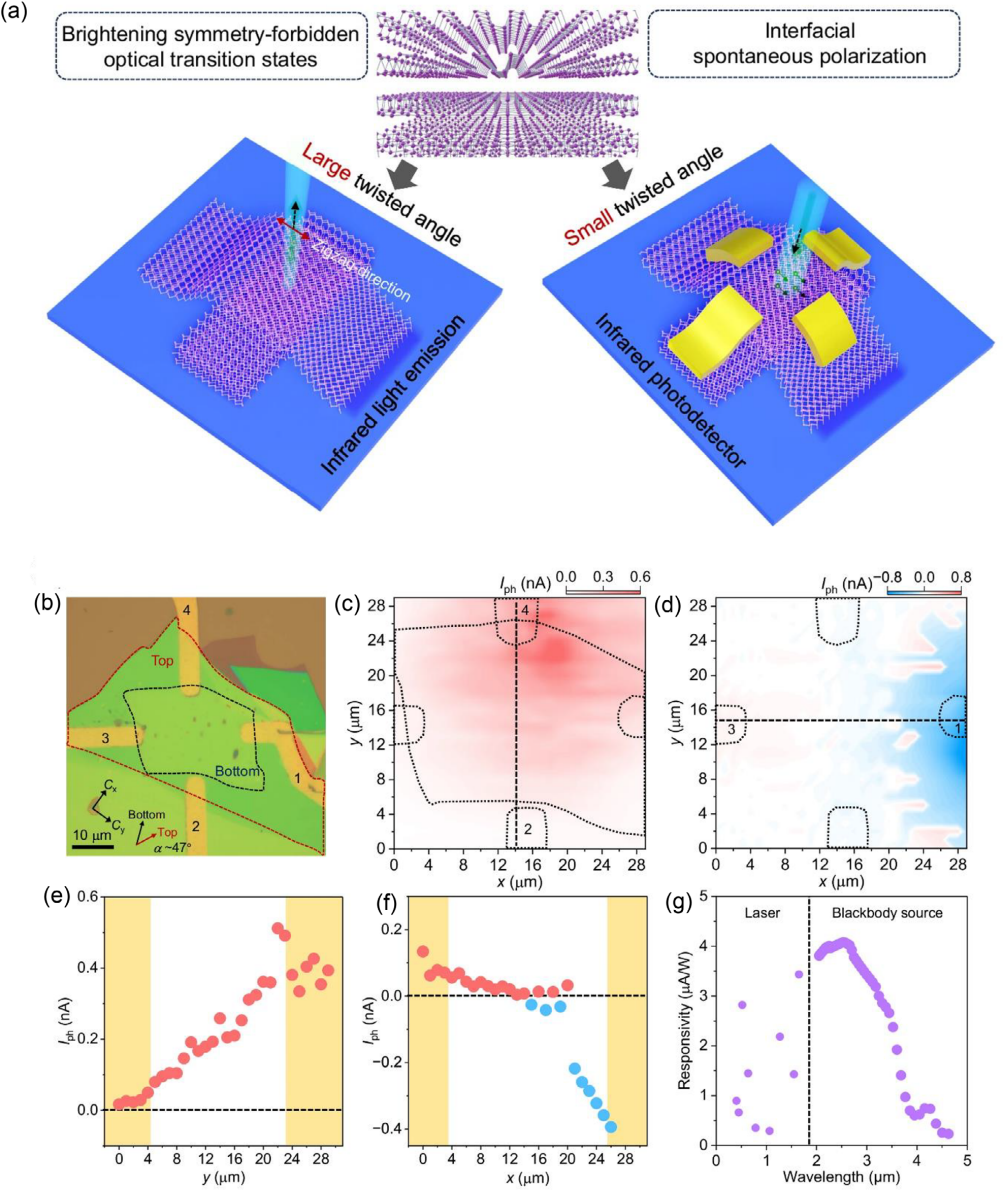
a) At large twist angles, the normally symmetry‐forbidden zigzag direction is followed by unusual emission of infrared light. At small twist angles, strong spontaneous polarization and BPVE dominate electron–hole pair separation. b) A twisted BP photodetector (Sample PA) with a twist angle of roughly 47° is depicted optically. The bottom and top BP layers are denoted by black and red dashed lines, respectively, and the top and bottom BP armchair directions are shown by black and red arrows. A scale bar is equivalent to 10 µm. Electrode pairs 2–4 c) and 1–3 d) exhibit photocurrent mappings (at zero voltage bias, λ = 520 nm), respectively. e) For electrode pairs 2–4, the photocurrent is shown in (c) as a dashed line. f) Electrode pair 1–3's photocurrent along the dashed line in (d). g) A blackbody radiation source for wavelengths above 2 µm and lasers with wavelengths between 0.4 and 1.7 µm were used as excitation sources in an experimental measurement of wavelength‐dependent responsivity for electrode pairs 2–4 at zero voltage bias. (a–g) Reproduced under the terms of the CC‐BY 4.0 license.^[^ ^74^ ^]^ Copyright 2024, Chen et al.

The main charge carriers in semiconductors, electrons and holes, control optical transitions and detection systems. Twisted vdWs heterostructures, which form atomically sharp interfaces, provide an effective way to control the radiation, separation, and collection of electron–hole pairs.^[^ ^94^ ^]^ Chen et al.^[^ ^74^ ^]^ showed that in vdW layered black BP, an infrared semiconductor with a highly anisotropic crystal structure and properties, twisted interfaces have a significant impact on electron–hole pair separation and recombination. On the one hand, infrared light emission from initially symmetry‐forbidden states along the zigzag direction is made possible by the twisted interface, which breaks the symmetry of optical transition states. However, effective electron–hole pair separation is made possible by spontaneous electronic polarization and BPVE at the twisted interface, which eliminates the need for an external voltage bias. In order to achieve effective electron–hole pair separation and collection without the need for an external voltage bias, twisted BP heterostructures are used to induce interfacial BPVE and activate symmetry‐forbidden polarized mid‐infrared light emission along the zigzag direction in thin‐film BP (**Figure** **7**a). Photocurrent measurements were used to further investigate the electron–hole pair separation processes in twisted BP. An optical image of a twisted BP photodetector (Sample PA) is shown in Figure 7b. Photocurrent characterizations were performed using electrode pairs 2–4, where at zero voltage bias, the carrier collection direction closely matches the Cx direction. The twisted region, as illustrated in Figure 7c,e, displays an unaltered polarity of photocurrent, in contrast to extrinsic PV effects like the photo‐thermoelectric effect and those brought on by intrinsic electric fields in Schottky junctions. In the Schottky junction effect, the intrinsic electric fields close to the two electrodes point in opposite directions, producing opposing photocurrents. In the photo‐thermoelectric effect, light creates a temperature gradient that pushes carrier diffusion toward the electrodes by raising the temperature of the lattice and carriers. A vanishing photocurrent is the result of carriers diffusing equally to both electrodes when the light spot is centered in the channel. Consequently, the photo‐thermoelectric effect generates a maximum photocurrent close to the electrodes and a minimum at the channel's center, much like the Schottky effect. The effects of the Schottky junction and photo‐thermoelectric contributions cannot be completely eliminated in mid‐infrared photocurrent measurements due to the large light spot from the mid‐infrared source. Nevertheless, their impact was lessened by placing the light spot in the middle of the channel. Furthermore, a wide spectral range up to 4 µm remains visible for the BPVE phenomenon (Figure 7g). A typical pattern can be seen in the photocurrents measured by electrode pairs 1–3 (along the Cy direction), which approach zero near the channel center and show opposite polarity near the two electrodes (Figure 7d,f). This suggests that the photo‐thermoelectric effect or the Schottky junction effect at the metal–semiconductor interface are the main factors controlling the electron–hole pair separation process. It was shown that BPVE happens in twisted structures over a broad range of BP flake thicknesses and twist angles. This makes it easier to fabricate twisted structures and expands the options beyond single‐ or bilayer vdW materials. Lastly, other vdW twisted systems might also benefit from this strategy. An efficient method for customizing the optoelectronic characteristics of materials and creating useful devices is to engineer vdW twisted interfaces.

### 5.2 Distorted 2D TMDs

Broken inversion symmetry in materials is the source of BPVE, which enables photocurrent generation without the application of an external electric field. This effect is especially noticeable in materials such as MoTe_2_, where the crystal lattice is distorted by mechanical strain or phase transitions, which leads to symmetry breaking and increased photocurrents. In this process, the material's symmetry properties—such as inversion symmetry breaking and crystal symmetries like ferroelectric or piezoelectric properties—are very important. By enhancing light absorption, carrier dynamics, and overall PV response, strain engineering fine‐tunes the material's electronic band structure. The efficiency of unconventional PV materials, such as 2D materials, can be significantly raised by optimizing strain, opening the door to more sophisticated energy harvesting technologies and higher power conversion efficiency.

The remarkably small energy gap (ΔE <50 meV) among TMDs makes molybdenum ditelluride (MoTe_2_)—which exhibits room‐temperature ferroelectricity—one of the most promising 2D layered nanomaterials for enabling phase transitions from the semiconducting 2H phase to the metallic 1T′ phase.^[^ ^89^, ^95^, ^96^ ^]^ The shift‐current model has been used in earlier studies to interpret the BPVE effect in distorted 1T (1T‴) 2D TMDs, such as MoTe_2_.^[^ ^19^, ^97^, ^98^ ^]^ This model suggests effective solar energy utilization driven by intrinsic crystal anisotropy. It predicts strong shift‐current responses in both bulk and monolayer 1T (1T‴) phases, with absorption spanning from the near‐infrared to the visible region.^[^ ^98^ ^]^ Additionally, compared to their monolayer counterparts, bulk 2D TMDs exhibit significantly stronger PV effects due to their smaller bandgaps and more delocalized valence band states.

**Figure 8:**
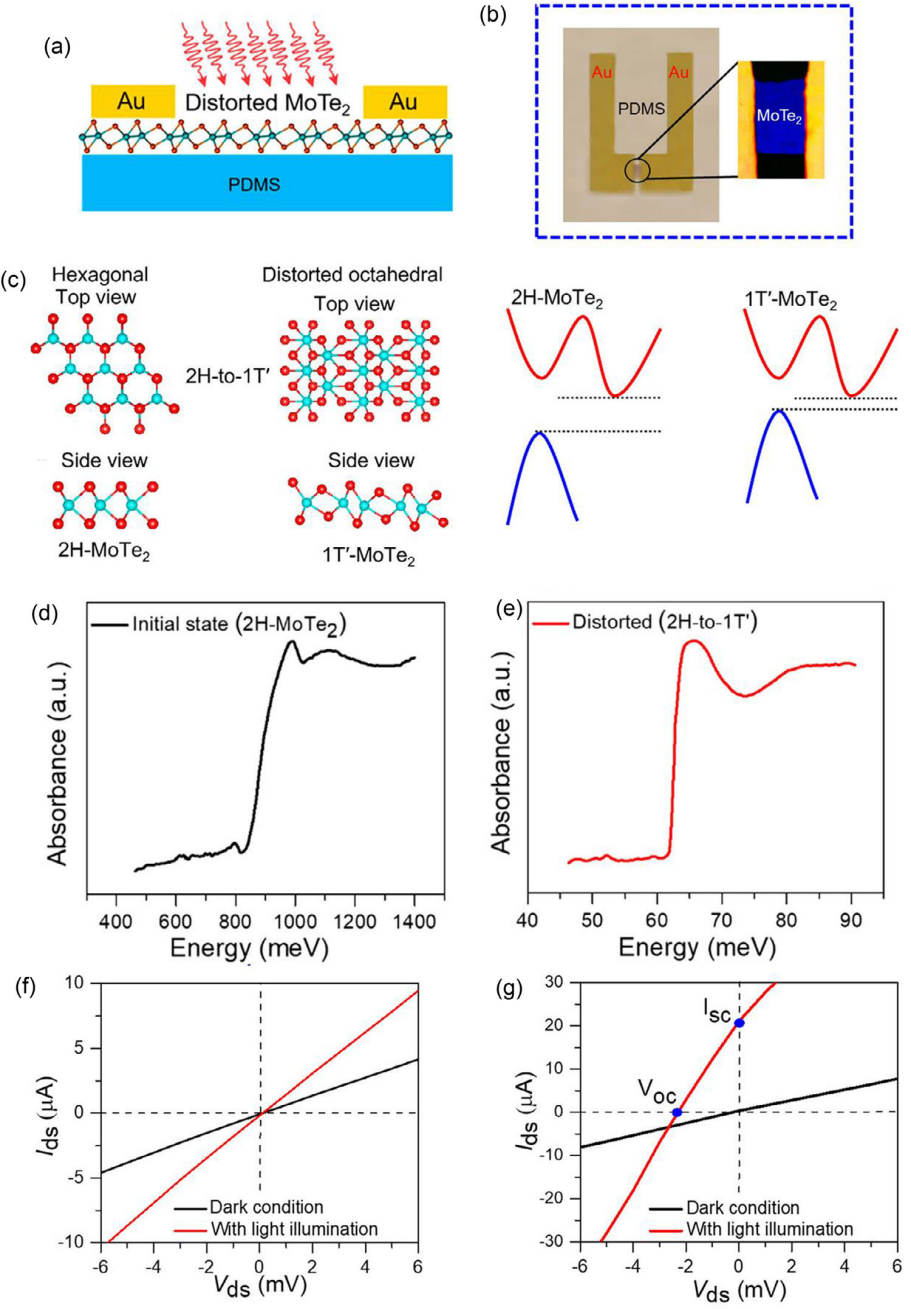
a) Distorted 2H‐MoTe_2_ (1T′‐MoTe_2_) nanoflake on a stretchable PDMS substrate is shown schematically. b) The sample on a stretched PDMS substrate with Au electrodes acting as electrical contact. c) Electronic and schematic band structures of MoTe_2_ before and after distorted states. d) 2H‐MoTe_2_ and e) distorted MoTe_2_ microbased FTIR absorption spectroscopy. f) *I*–*V* characteristics of a 2H‐MoTe_2_ nanoflake demonstrating the photoconductive effect. g) *I*_ds_–*V*_ds_ characteristics of a distorted MoTe_2_ nanoflake showing induced photocurrent under visible light illumination. (a–g) Reproduced with permission.^[^ ^43^ ^]^ Copyright 2023, ACS.

In the 2D material MoTe_2_, Aftab et al.,^[^ ^43^ ^]^ discovered mechanical distortion‐induced bulk PV behavior, which they attributed to phase transition and the breaking of inversion symmetry in the crystal structure. It is possible to control or modify the electronic and optoelectronic properties of 2D materials by effectively tuning their mechanical and structural properties through strain engineering.^[^ ^99^ ^]^ Mechanical deformation was utilized to cause a phase transition in MoTe_2_ nanoflakes from 2H‐MoTe_2_ to 1T′‐MoTe_2_ in order to lower the optical bandgap and break symmetry in multilayer MoTe_2_ nanocrystals and enable BPVE effects. A BPVE device configuration based on distorted 2H‐MoTe_2_ (1T′‐MoTe_2_) nanoflakes on a stretchable PDMS substrate is shown schematically in **Figure** **8**a, and an actual optical microscopy image of the device with distorted octahedral 1T′‐MoTe_2_ on a stretched PDMS stamp is shown in Figure 8b. The initial schematic band structure of 2H‐MoTe_2_ and its state after distortion are shown in Figure 8c, along with top and side views of various 2D phases and schematic representations of various phase structures, including 2H‐to‐1T′ (also referred to as 1T″) transitions. The optical absorption spectra of 2H‐MoTe_2_ and 1T′‐MoTe_2_ showed absorption band edges near 800 and 63 meV, respectively (Figure 8d,e). The absorption spectroscopy of the multilayered MoTe_2_ was carried out using micro‐based Fourier transform infrared spectroscopy (FTIR). The results are in line with a prior study that found that 1T′‐MoTe_2_ has a bandgap of up to 60 meV because of strong coupling between spins and orbits in various bands, according to density functional theory simulations.^[^ ^91^ ^]^ The 2H phase's bandgap is in good agreement with its semiconducting behavior, whereas the 1T′ phase's bandgap is indicative of the narrow gap found in MoTe_2_'s semi‐metallic state.^[^ ^91^ ^]^ Another study found that 1T′‐MoTe_2_ deposited using CVD showed characteristics that were in line with its metallic nature.^[^ ^100^ ^]^ As seen in Figure 8f, the photoconductive device based on 2H‐MoTe_2_ displays representative *I*–*V* curves, demonstrating a photoreaction with a photocurrent to dark current ratio of 45:1 at a −6 mV bias. The *I*–*V* properties of a distorted MoTe_2_ (2H to 1T′) device on a PDMS substrate with effectively broken inversion symmetry,^[^ ^101^, ^102^ ^]^ are shown in Figure 8e. A common self‐biased phenomenon is observed, producing a current in the presence of uniform illumination without a junction or interface. The photocurrent induced as a bulk PV is comparable to the maximum photovoltage induced of ≈−2.33 mV, obtained (at I_ds_ = 0 A), and a positive photocurrent response of 21 µA, obtained (at V_ds_ = 0 V). This phenomenon is clearly observed in nanocrystals with broken inversion symmetry in the absence of external fields.^[^ ^103^, ^104^ ^]^ The results, which were obtained from a wide variety of well‐known TMD‐based nanomaterials, highlight unique characteristics of the photocurrent responses induced by in‐plane polarity and its potential. This method provides a state‐of‐the‐art method to maximize photon conversion efficiency into electrical power for optoelectronic power harvesting devices.

### 5.3 Rolling 2D TMDs

One particularly notable advancement in TMD research is the conversion of flat 2D nanosheets into 1D structures such as nanoscrolls (NSs) and nanotubes (NTs).^[^ ^105^, ^106^ ^]^ There are several ways to accomplish this rolling process. When a drop of ethanol is applied to monolayers grown by CVD, for example, self‐assembly triggered by solvent interaction can cause rapid scrolling with a yield of almost 100% in a matter of seconds. Alternatively, TMD films can be patterned using methods such as focused ion beam (FIB) etching to create ribbons, allowing for the production of nanoscroll arrays with precise length and chirality.^[^ ^105^, ^107^ ^]^ The crystallographic orientation of the original 2D sheet determines the chirality and rolling direction of these 1D structures. The design of particular NT configurations based on TMD unit cells is made easier by computational tools like Chiral tube.^[^ ^107^ ^]^ Compared to their 2D counterparts, rolled 1D TMDs offer a number of advantages. Compared to flat monolayers, electron mobility in NSs can be increased by up to 30 times, providing better performance in electronic applications. They are perfect for sensing, catalysis, and hybrid devices because of their cylindrical topology, which creates adjustable vdWs gaps that allow molecules or other materials to be encapsulated. Furthermore, these rolled structures improve mechanical robustness and flexibility while preserving the inherent qualities of the 2D materials. Additionally, they facilitate the development of new heterostructures by incorporating various TMDs into intricate device designs. There are a number of differences between flat 2D TMD nanosheets and their rolled 1D counterparts. While rolled TMDs facilitate the development of flexible electronics, energy storage systems, and sophisticated sensors, 2D sheets are best suited for planar devices such as field‐effect transistors (FETs) and photodetectors.

A practical technique for producing 2D TMD nanoflakes on a large scale is roll‐to‐roll (R2R) deposition.^[^ ^105^, ^107^, ^108^ ^]^ This technique allows for the continuous printing of thin films up to 100 mm wide at high speeds of 10 mm s^−1^. R2R supports both single‐layer and multilayer film formation and also makes it possible to create heterojunctions between various TMDs. The optoelectronic performance of films made using this technique is comparable to that of their batch‐processed counterparts, opening the door for inexpensive, highly effective devices that can be used in solar energy conversion and other industrial applications. In the future, rolled TMD nanostructures like NSs and NTs are attracting interest due to their potential in a variety of applications, such as high‐mobility transistors, sensors, energy storage and conversion devices, flexible and wearable electronics, catalysts, and multipurpose hybrid architectures. With scalable techniques like R2R deposition and the capacity to regulate rolling dynamics and chirality, rolled TMDs are positioned as key components for upcoming nanoelectronics and next‐generation device engineering.

**Figure 9:**
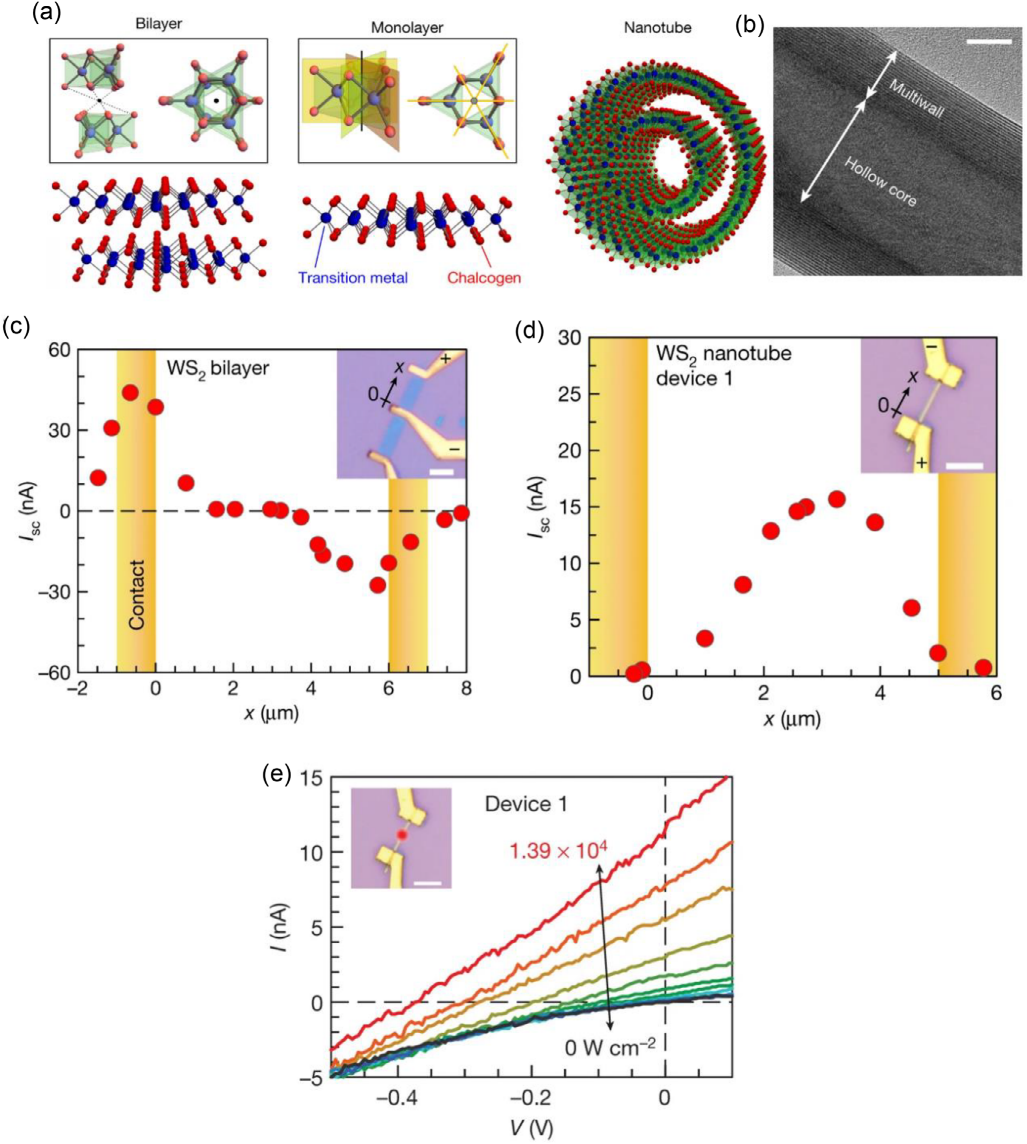
a) Three different symmetries of WS_2_ devices—a bilayer, a monolayer, and a multiwall NT—are shown in terms of their crystal structures. The center of spatial inversion symmetry in the bilayer is denoted by certain points. Mirror planes and a threefold rotation axis are two examples of the monolayer's unique symmetry features. b) Hollow‐core multiwall WS_2_ NT is shown in a TEM image. A white scale bar indicates that the NT is 10 nm in length. c) A WS_2_ monolayer device's I_SC_ variation with laser spot position demonstrates that PV response occurs only in the vicinity of the contact area when exposed to laser illumination. d) WS_2_ NT device's I_SC_ fluctuates according to the location of the laser spot; the main reaction happens when the laser shines on the device's center, away from the contacts. This suggests a BPVE. e) A laser with a wavelength of 632.8 nm was used to measure the *I*–*V* characteristics under various illumination intensities. An optical micrograph of the apparatus, with the laser spot fixed at the center of the NT, is included in the inset. A white scale bar is equivalent to 4 µm. (a–e) Reproduced with permission.^[^ ^73^ ^]^ Copyright 2019, Nature.

According to Zhang et al.,^[^ ^73^ ^]^ BPVE was discovered in devices based on tungsten disulfide, a member of the TMD family. BPVE is greatly increased by methodically reducing the crystal symmetry beyond broken inversion symmetry, which involves changing from a 2D monolayer to a NT with polar properties. Group‐VI‐B TMDs, including WS_2_, are 2D semiconductors with bandgaps between 1.2 and 2.1 eV.^[^ ^109^, ^110^ ^]^ In the stable 2H phase, the unit cell of bulk TMDs is centrosymmetric and belongs to the D6h point group. However, by isolating a single layer, inversion symmetry can be broken because the unit cell consists of a bilayer (**Figure** **9**a). A typical transmission electron microscopy (TEM) image of multiwall NTs with a hollow core is shown in Figure 9b. To differentiate BPVE from the Schottky barrier PV effect and the photothermal effect near the contacts, the laser spot was scanned from one electrode to the other.^[^ ^54^, ^111^ ^]^ Only when the contacts in the WS_2_ bilayer device are exposed to laser light does a PV response occur; when the WS_2_ flake is the only component illuminated, no signal is detected (Figure 9c). On the other hand, when the center of the NT is illuminated, the WS_2_ NT device increases dramatically (Figure 9d). This significant increase, despite the distance from the contacts in NT devices, cannot be attributed to variations in light absorption. Although the NTs' multiwall structure might result in more light absorption than a monolayer, the absorbed light intensity is only twice as high as that of a WS_2_ monolayer when the diameter of the NT is compared to the size of the laser spot. The *I*–*V* characteristics at different laser power levels are displayed in Figure 9e. The V_OC_ and both fluctuate monotonically with *P*_laser_. BPVE is greatly improved by systematically reducing the crystal symmetry beyond simple broken inversion symmetry, for example, from a 2D monolayer to a polar NT. Other BPVE materials have a photocurrent density that is orders of magnitude lower than this one. These results highlight the potential of TMD‐based nanomaterials as well as the more general significance of crystal symmetry reduction in raising solar‐to‐electric power conversion efficiency.

## 6 Strained 2D TMDs

Taking advantage of the remarkable mechanical flexibility of 2D TMDs, strain engineering provides a powerful means to customize their electrical, optical, and mechanical characteristics.^[^ ^112^, ^113^ ^]^ As demonstrated in monolayer MoS_2_, applying strain can cause important changes, including tuning the band structure, which can result in direct‐to‐indirect bandgap transitions or even semiconductor‐to‐metal phase changes.^[^ ^112^, ^113^ ^]^ These effects differ amongst TMDs and are contingent upon the type and magnitude of the applied strain. In order to facilitate transitions between semiconducting (2H phase) and metallic (1T' phase) states, strain can also change the phase of TMDs.^[^ ^71^ ^]^ This is particularly crucial for phase engineering in a variety of device applications and for forming metal‐semiconductor‐metal junctions.^[^ ^114^ ^]^ Additionally, strain modifies optical responses, allowing for control over nonlinear phenomena such as second‐harmonic generation (SHG), which is a sensitive indicator of local strain, as well as absorption and emission.^[^ ^112^ ^]^ The mechanical strength of 2D TMDs significantly exceeds that of bulk semiconductors, allowing for localized and reversible property modulation. Several strain engineering methods, including alloying,^[^ ^113^ ^]^ kirigami,^[^ ^114^, ^115^ ^]^ substrate patterning, and mechanical deformation,^[^ ^43^ ^]^ enable fine control at the nanoscale. These advances establish strained 2D TMDs as essential components for next‐generation technologies by making them perfect for a variety of uses such as sensors, phase‐change memory, tunable optoelectronics, and quantum photonics.^[^ ^112^, ^113^, ^114^, ^115^ ^]^

**Figure 10:**
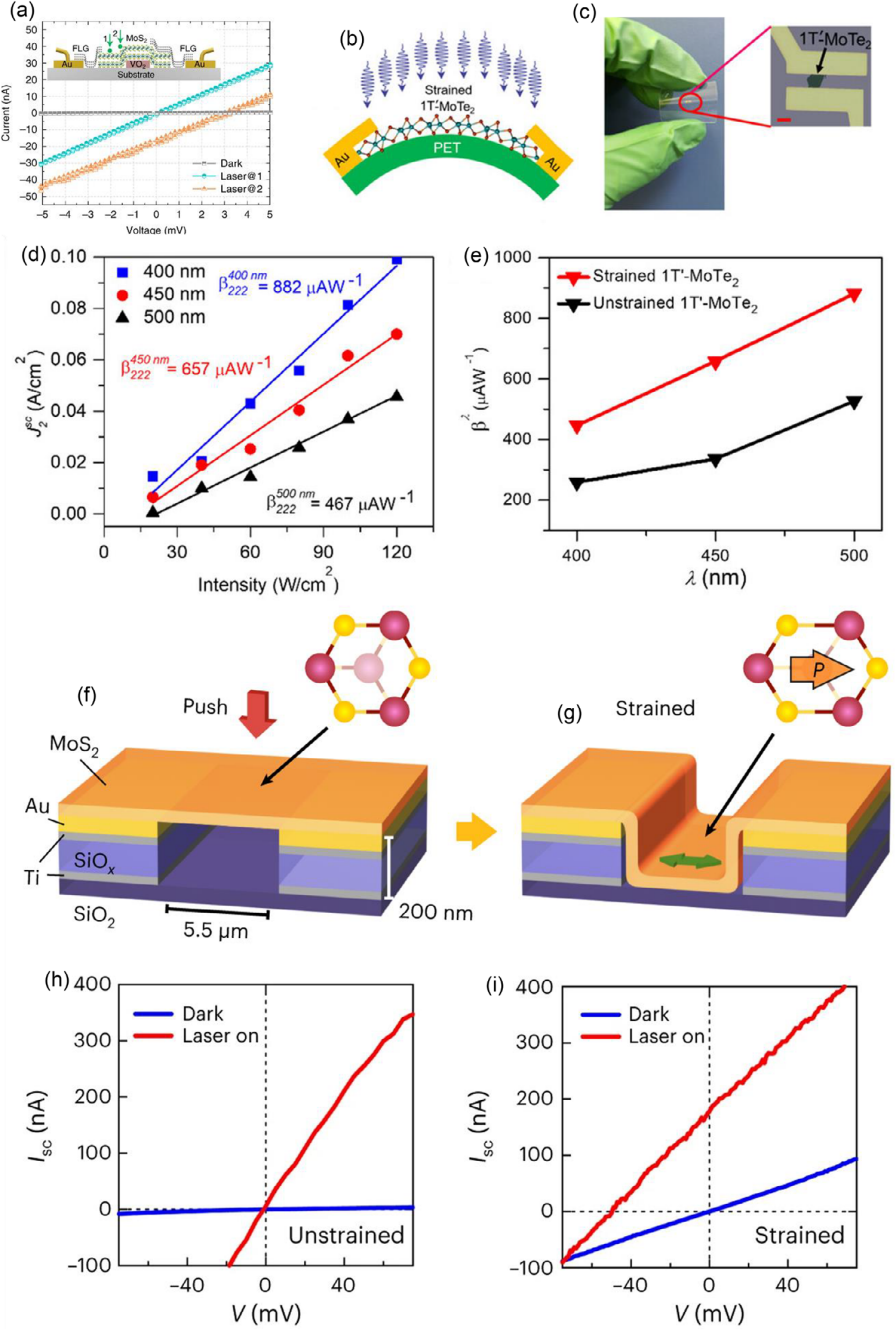
a) *I*–*V* characteristics of the device. The inset presents a schematic of its cross‐sectional view. Reproduced with permission.^[^ ^116^ ^]^ Copyright 2021, Nature. b) A flexible polyimide substrate with strained 1T′‐MoTe_2_. c) A captured image of the device on a flexible substrate. d) Current density of strained 1T′‐MoTe_2_ as a function of incident light intensity at various wavelengths where the slope indicating the BPVE tensor component. e) BPVE tensor for strained and unstrained 1T′‐MoTe_2_ nanoflakes as a function of incident light wavelength. (b–e) Reproduced with permission.^[^ ^71^ ^]^ Copyright 2019, Wiley. Diagrammatic representation of a fully strained 3R‐MoS_2_ flake f) a suspended 3R‐MoS_2_ flake in its intermediate state, g) on a substrate with a pre‐patterned design. In this device structure, strain is created by pressing the flake all the way to the bottom of the channel. h) The *I*–*V* characteristics of an unstrained 3R‐MoS_2_ device and i) a strained (≈0.18%) 3R‐MoS_2_ device (laser power = 70 µW). (f–i) Reproduced with permission.^[^ ^72^ ^]^ Copyright 2023, Nature.

The flexo‐PV effect, also known as the strain‐gradient‐induced BPVE, can be triggered in centrosymmetric semiconductors through strain‐gradient engineering, greatly expanding the materials' potential for future energy and sensing applications. Using a strain‐gradient engineering technique that takes advantage of the structural inhomogeneity and phase transition of a hybrid system made up of MoS_2_ and VO_2_, Jie et al.^[^ ^116^ ^]^ reported an experimental demonstration of the flexo‐PV effect in the prototypical 2D material MoS_2_. As illustrated in **Figure** **10**a, current–voltage curves were measured with illumination from Laser@1 and Laser@2, as well as without illumination (Dark). Compared to the majority of non‐centrosymmetric materials, MoS_2_ exhibits an experimental bulk PV coefficient that is orders of magnitude higher. The fundamental relationship between strain gradients and the flexo‐PV effect in low‐dimensional materials is demonstrated by research, which could help identify new optoelectronic phenomena in materials that have strain gradient engineering. In another report, BPVE in 1T′‐MoTe_2_ was first experimentally demonstrated by Aftab et al.^[^ ^71^ ^]^ A schematic of 1T′‐MoTe_2_ on a flexible substrate is shown in Figure 10a, and a photograph of the deformed device being manually worked on a flexible polyimide (PET) substrate is shown in Figure 10b. An enlarged optical microscopy image of a strained 1T′‐MoTe_2_ nanoflake on PET is shown in the inset of Figure 10c. These findings confirm that the broken inversion symmetry in 1T′‐MoTe_2_ has been successfully investigated.^[^ ^101^, ^102^ ^]^ The slopes of linear fits at different wavelengths were used to analyze the wavelength dependence of BPV tensor 𝛽^𝜆^ in strained 1T′‐MoTe_2_, as illustrated in Figure 10d. As the incident wavelength decreased from 500 to 400 nm, the BPV tensor 𝛽^𝜆^ value rose dramatically from 467 to 882 A W^−1^. This suggests a proportional relationship between the incident light's wavelength and the BPV tensor components, as shown in Figure 10e. This improvement is probably the result of increased strain causing a larger shift vector in the BPV mechanism, which raises the BPV voltage or current and intensifies BPVE as a whole. These results demonstrate how strained 1T′‐MoTe_2_ can be used to create tunable and effective optoelectronic devices, providing a platform that unites metallic and semiconducting behavior and opening up possibilities for the creation of next‐generation material systems.

Strain‐induced polarization has been shown by Dong et al.^[^ ^72^ ^]^ to significantly increase BPVE in non‐centrosymmetric rhombohedral‐type MoS_2_ multilayer flakes (3R‐MoS_2_). The coupling between internal polarization and mechanical strain results in an enhanced BPVE response. Interestingly, the enhancement is mainly seen along the armchair direction of the 3R‐MoS_2_ lattice, whereas BPVE is primarily unaffected along the zigzag direction. This indicates that the effect is strongly dependent on crystallographic orientation. This anisotropic behavior highlights the significance of strain direction and lattice symmetry in optimizing PV performance in 2D materials. A specially made pre‐patterned substrate with two parallel steps was created in order to study BPVE in 3R‐MoS_2_ under strain. This substrate provided a controlled platform for applying uniaxial strain. This stepped substrate was then covered with exfoliated 3R‐MoS_2_ flakes (Figure 10f,g). This allowed for accurate control of the mechanical deformation and a methodical examination of the strain‐induced polarization that resulted and its impact on the bulk PV response. Unstrained 3R‐MoS_2_ flakes were found to have only a photoconductive effect, with a negligible Isc under illumination (Figure 10h). This suggests that BPVE is either nonexistent or very minimal in these flakes in the absence of strain‐induced polarization. The strained region (0.18%) in the channel, on the other hand, shows a significant I_SC_ of 170 nA at 70 × µW illumination (Figure 10i), indicating that strain‐induced polarization in 3R‐MoS_2_ significantly enhances BPVE. This method reveals how strain engineering can improve PV performance, providing a viable route to the creation of cutting‐edge optoelectronic devices. To expand the functional scope of next‐generation energy and sensing technologies, this study may stimulate the investigation of novel photoelectric processes in strained 2D layered materials and their vdWs heterostructures by utilizing strain‐induced effects, such as the piezo PV and flexo‐PV phenomena.

## 7 Van der Waals Heterostructures

The utilization of BPVE has been transformed by vdWs heterostructures, which use interfacial interactions to disrupt crystal symmetries and facilitate the production of ultrafast, efficient photocurrents. Achieving an intrinsic response time of 26 ps and a high BPVE coefficient of 0.6 V^−1^ requires dual polarization mechanisms, such as those found in MoS_2_/BP structures, where in‐plane and out‐of‐plane polarizations work in concert to improve carrier transport.^[^ ^66^ ^]^ Through nano‐edge configurations, edge engineering creates geometric asymmetries that generate polarization‐dependent photocurrents and tunable photoresponses in systems such as ReS_2_/ReS_2_ and WS_2_/ReS_2_.^[^ ^117^ ^]^ Meanwhile, contact‐free symmetry breaking and the creation of spin and charge photocurrents with adjustable directionality are enabled by magnetic proximity effects in heterostructures like MnBi_2_Te_5_/CrI_3_, increasing functionality without sacrificing host material characteristics.^[^ ^118^ ^]^ All of these methods show how vdW heterostructures can be used to overcome BPVE limitations, opening the door to next‐generation energy‐harvesting and optoelectronic applications.

**Figure 11:**
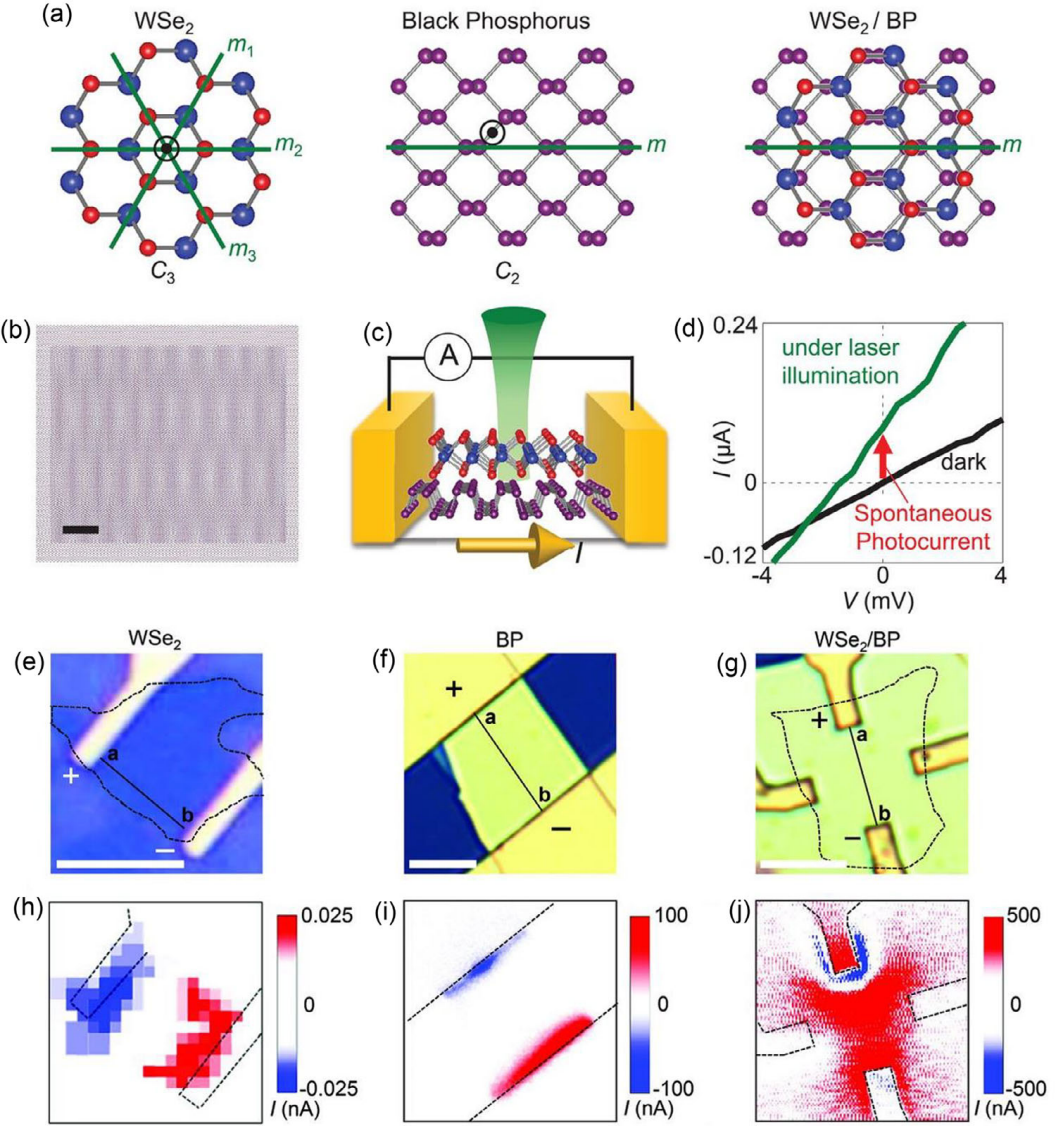
a) Schematic representations of the WSe_2_/BP heterointerface (right), monolayer WSe_2_ (left), and BP (middle). Rotational axes and mirror planes are indicated by circled dots and green lines, respectively. b) Moiré patterns of the WSe_2_/BP heterointerface with a scale bar for 5 nm when the WSe_2_ and BP mirror planes are aligned. c) Experimental schematic, in which the electrical system for measuring photocurrent is indicated by the circle "A." d) WSe_2_/BP device's *I*–*V* characteristics. Optical Images of devices for e) monolayer WSe_2_, f) BP, and g) WSe_2_/BP heterointerface. Scale bars (white lines) indicate 5 µm. Under linearly polarized 532‐nm light, photocurrent mapping of h) monolayer WSe_2_, i) BP, and j) WSe_2_/BP devices shows the emergence of SPE at the WSe_2_/BP interface center. (a–j) Reproduced with permission.^[^ ^70^ ^]^ Copyright 2021, Science.

Rotating layers of one material or creating heterointerfaces between different materials can result in unique properties at the vdW material interface. Akamatsu et al.^[^ ^70^ ^]^ intentionally broke in‐plane inversion symmetry by combining BP, which has twofold rotational symmetry, with tungsten diselenide, which has threefold rotational symmetry, to form such an interface. A shift current mechanism is responsible for the spontaneous PV effect that is limited to the polarization direction as a result of this configuration's induction of in‐plane electronic polarization. BP and WSe_2_ were selected as the interface's building blocks due to their unique mirror and rotational symmetries. Whereas BP has twofold rotational symmetry with its own set of mirror planes, WSe_2_ has threefold rotational symmetry with mirror planes oriented along the armchair direction. The deliberate breaking of in‐plane inversion symmetry at the interface is enabled by these differing symmetry elements, which are shown in **Figure** **11**a as green lines for mirror planes and circled dots for rotational axes. However, due to the twofold rotational symmetries of BP and the threefold symmetries of WSe_2_ are incompatible, the heterointerface formed by these two materials lacks rotational symmetry. If the mirror planes of the two materials are parallel, mirror symmetry can still be maintained. When only one mirror plane remains, electronic polarization is anticipated to develop parallel to it, generating photocurrent along the in‐plane polar direction. In contrast to the hexagonal Moiré patterns commonly observed in twisted Gr, a stripe Moiré pattern appears at the WSe_2_/BP interface (Figure 11b) both parallel and perpendicular to the mirror plane, as well as along the polarization direction. The lattice mismatch between WSe_2_ and BP gives rise to this stripe pattern, which reflects the combined effect of BP's anisotropic potential and WSe_2_'s trigonal symmetry, which together produce the in‐plane polarity seen at the interface. A laser was used to light a monolayer of WSe_2_ from the WSe_2_ side onto BP that was between 40 and 50 nm thick (Figure 11c; see Supplementary Materials for details). The WSe_2_/BP interface's *I*–*V* characteristics at room temperature are displayed in Figure 11d, which contrasts the response in linearly polarized light (green) and darkness (black). In contrast to the absence of light, a spontaneous photocurrent—represented as an I_SC_ under zero bias—was detected upon laser illumination (λ = 532 nm, intensity of 1.44 Mw). The spatial distribution of spontaneous photocurrent in monolayer WSe_2_ (Figure 11e,h), BP (Figure 11f,i), and the WSe_2_/BP interface (Figure 11g,j) was systematically analyzed. A laser spot was scanned across the corresponding devices in Figure 11e–g to obtain the photocurrent mapping images in Figure 11h–j. When the laser shone on the center of the monolayer WSe_2_ (Figure 11e) and BP device (Figure 11f), no spontaneous PV effect (SPE) was seen. The antisymmetric spatial profile of the photocurrent, which was only produced close to the electrodes, was probably brought on by either Schottky barrier effects, photothermal effects, or a combination of the two.^[^ ^119^, ^120^ ^]^ The SPE in the WSe_2_/BP stacking device was caused by symmetry changes at the heterointerface, as evidenced by the detection of photocurrent even when the laser spot was placed far from the electrodes (Figure 11j). This interfacial SPE was consistently observed in several devices. Furthermore, the position‐dependent measurements along the lines in Figure 11g revealed antisymmetric photocurrent patterns surrounding the electrodes. The findings offer a simple symmetrical engineering guideline that can be used with a variety of vdW interfaces.

## 8 Piezoelectric 2D Materials

In 2D systems, shift current generation under uniform light relies on enhanced quantum geometric features such as symmetry‐breaking and Berry curvature. Strain‐induced ferroelectricity in materials such as CuInP_2_S_6_ (CIPS) and strain‐induced piezoelectricity in monolayer MoS_2_ both dramatically increase photocurrent by introducing in‐plane polarization and changing band structures.^[^ ^69^, ^121^ ^]^ Interestingly, CIPS has photocurrent densities up to 100 times greater than those of bulk perovskite oxides and polar ordering at room temperature.^[^ ^43^, ^69^ ^]^ Additional tuning options are provided by phase transitions in materials such as MoTe_2_ and anisotropic behavior in WS_2_ and ReS_2_, and device performance is further improved by stacking heterostructures and external fields.^[^ ^43^, ^121^ ^]^ Scaling up these non‐centrosymmetric structures and reaching theoretical efficiency potential are still difficult tasks, though. However, combining ferroelectricity, piezoelectricity, and optical absorption in vdW heterostructures creates opportunities for ultrathin PV and optoelectronic technologies of the future.

**Figure 12:**
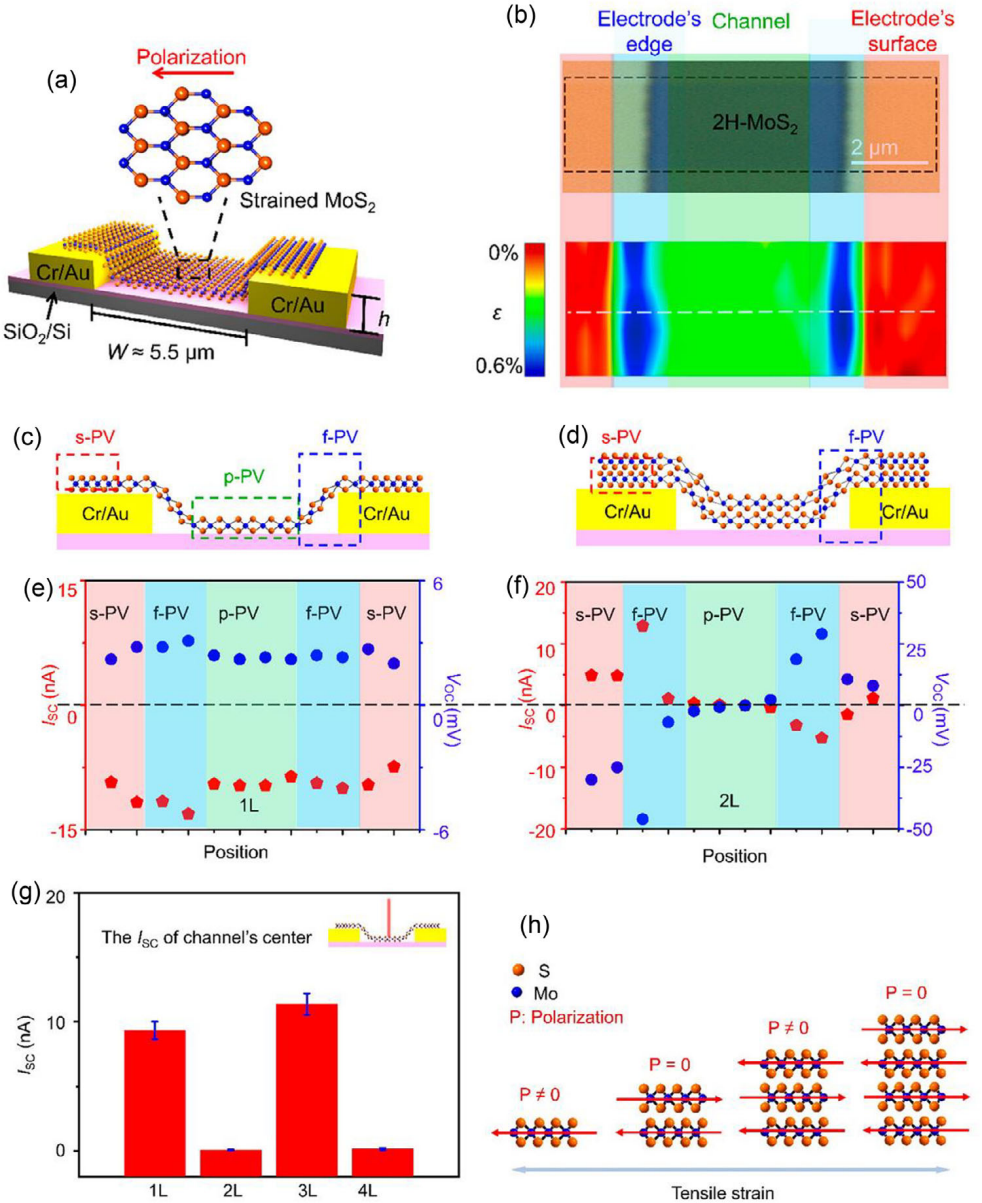
a) Schematic of the strained MoS_2_ device. b) Monolayer MoS_2_ device with an electrode height of 200 nm, along with the associated strain distribution and optical image. Distinct regions are represented by different background colors: the area close to the electrode edges is blue, the channel center is green, and the electrode surface is red. c) Schematic diagram showing the three PV mechanisms—the Schottky‐induced PV (s‐PV) effect, the piezo‐photovoltaic (p‐PV) effect, and the flexo‐photovoltaic (f‐PV) effect—in the strained monolayer MoS_2_ device. d) Schematic diagram showing the strained bilayer MoS_2_ device's two PV mechanisms. Spatial distribution of I_SC_ and V_OC_ on the strained e) monolayer, f) bilayer. g) Layer‐dependent in strained MoS_2_. h) Mechanism of the p‐PV effect. (a–h) Reproduced with permission.^[^ ^69^ ^]^ Copyright 2024, ACS.

BPVE is an effective way for noncentrosymmetric bulk materials to generate electricity from light energy. It is yet unknown, however, if this effect holds true when the material thickness is lowered to the atomic level. Wei et al.^[^ ^69^ ^]^ showed how strain‐induced polarization in noncentrosymmetric mono‐ and few‐layer 2H‐MoS_2_ crystals can produce PV outputs, demonstrating the piezo‐PV effect in atomically thin 2D materials. A prepatterned substrate comprising two metal contacts with steps of controlled heights was utilized to apply strain to the 2H‐MoS_2_ crystals and investigate the corresponding piezo‐PV effects (**Figure** **12**a). The optical image (Figure 12b, top panel) shows that the atomically thin MoS_2_ crystals remain continuous after transfer, with no discernible cracks or breaks at the nanometer scale. The PV responses were further measured by scanning the laser spots across various MoS_2_ device regions in order to look into the source of the observed BPVE effect (Figure 12b,c). Since inversion symmetry breaking only happens in MoS_2_ with an odd number of layers, the thickness of the MoS_2_ is also varied from one to four layers. MoS_2_ devices with an odd number of layers can be found to have a nonzero photocurrent when they are illuminated in all three different regions, as illustrated in Figure 12e,f. On the other hand, MoS_2_ devices with even numbers of layers do not show any PV response when illuminated at the channel region. When light is applied at or close to different electrodes in these situations, the signs of V_OC_ and I_SC_ also change. These findings imply that distinct mechanisms may be responsible for the observed PV effects in each location. The PV response in the electrode region is attributed to the Schottky barrier effect where MoS_2_ crystals and Au electrodes with various work functions come into contact,^[^ ^122^, ^123^ ^]^ which separates the photoinduced carriers. In this region, MoS_2_ maintains inversion symmetry (in the case of two and four layers) and is not under any strain. Additionally, since the PV effect is present in MoS_2_ of all thicknesses examined, it cannot be ascribed to any noncentrosymmetric mechanism in the strain‐gradient region. Even in centrosymmetric 2D semiconductors, where a nonzero dipole moment results from the nonuniform displacement of atoms within the crystal lattice, it has been reported that a strain gradient can cause BPVE effects.^[^ ^116^, ^124^ ^]^ In this situation, the so‐called flexo‐PV effect also applies, explaining why the strain gradient points in opposite directions close to the two electrodes. In fact, the values the V_OC_ and I_SC_ is consistent with those of the flexo‐PV effect in multilayer MoS_2_, which are roughly 3 mV and 20 nA, respectively.^[^ ^116^ ^]^

The layer‐dependent PV effect in the channel region indicates that the noncentrosymmetric lattice structure is closely linked to its origin (Figure 12g). Furthermore, for both single‐ and trilayer MoS_2_ devices, I_SC_ and V_OC_ show the same sign in the channel region. This enables the piezo‐PV effect to the PV responses. In particular, the in‐plane piezoelectric field effectively separates the photogenerated carriers in MoS_2_ flakes with an odd number of layers, resulting in the piezoelectric effect, leading to the I_SC_. A is used to maintain the net current at zero in order to offset the photocurrents. However, due to the opposite lattice orientations of neighboring layers, where the dipole moments cancel each other out, MoS_2_ crystals with an even number of layers are nonpiezoelectric (Figure 12h). To provide more evidence for the importance of noncentrosymmetric lattices in relation to the p‐PV effect. The device's PV performance was negligible when illuminated in the channel region due to the lack of asymmetry in bulk‐like MoS_2_ crystals. These findings also shed light on the mechanisms underlying the piezo‐PV effect in 2D materials in which the thicknesses are atomic.

## 9 Ferroelectric 2D Materials

Ferroelectric 2D materials have emerged as highly promising candidates for exploiting BPVE, which could result in higher photocurrent generation and efficiencies beyond the SQ limit, by exploiting their inherent lack of inversion symmetry and spontaneous polarization—without the need for p‐n junctions. Among the notable examples are CIPS, which exhibits a switchable photovoltage of ≈1.0 V and photocurrent densities of ≈10 mA cm^−^^2^.^[^ ^42^ ^]^ It performs best in ultrathin films (less than 40 nm); α‐In_2_Se_3_, which combines a narrow bandgap with strong polarization to achieve broadband visible‐light response and photocurrent densities that are almost 100 × greater than those of conventional ferroelectrics; and 3R‐WS_2_, which provides reconfigurable, non‐volatile photoresponses through polarization‐controlled Fermi levels, allowing for applications such as retinomorphic in‐sensor computing.^[^ ^42^, ^68^ ^]^ Meanwhile, 3R‐WS_2_ demonstrates a reconfigurable and nonvolatile BPVE in which photocurrent is controlled by the direction of ferroelectric polarization; it further supports retinomorphic in‐sensor computing through ferroelectric modulation of Fermi levels in Gr electrodes.^[^ ^68^ ^]^ These remarkable advances are underpinned by unique mechanisms intrinsic to 2D systems: the dimensionality effect minimizes carrier recombination and boosts shift currents compared to 3D counterparts; ferroelectric switching enables dynamic control over the direction and magnitude of photocurrents for adaptive optoelectronic devices; and symmetry engineering, such as interlayer sliding in bilayer crystals, provides unswitchable in‐plane BPVE and switchable out‐of‐plane responses, significantly expanding the functional design space.^[^ ^42^, ^125^ ^]^

**Figure 13:**
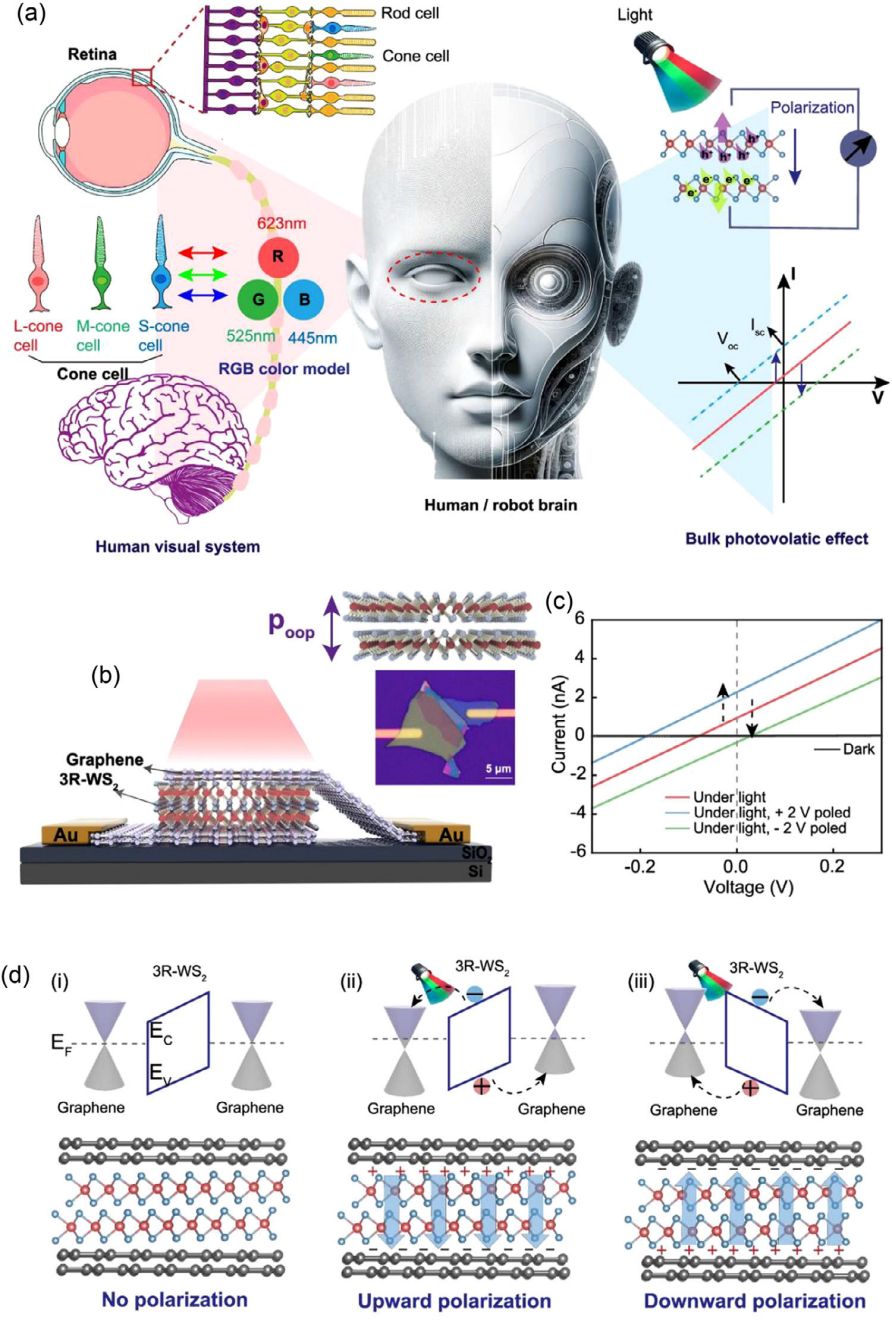
a) Schematic illustration of an artificial retinal vision system with L/M/S cones and a 2D ferroelectric BPVE inspired by the retina. b) Schematic and optical microscope image of the Gr/3R‐WS_2_/Gr retinomorphic device's crossbar structure is shown. The crystal structure's sulfur and tungsten atoms are represented by the blue and red spheres, respectively, and the spontaneous polarization's direction is shown by the purple arrows. c) Distinctive *I*–*V* curve shows both positive and negative PV behavior at different poling voltages. d) Gr/3R‐WS_2_/Gr energy band diagrams in various polarization states under light show how ferroelectric polarization affects the production and movement of photogenerated carriers. The integrated electric fields are shown by orange arrows, and the top and bottom Gr electrodes are represented by gray atoms. (a–d) Reproduced under the terms of the CC‐BY 4.0 license.^[^ ^68^ ^]^ Copyright 2025, Gong et al.

Reconfigurable and nonvolatile photoresponsivity must be implemented on hardware in order to advance in‐sensor computing for machine vision applications. However, the SQ limit limits the photoelectric efficiency of p‐n junctions, which is the primary source of current reconfigurable photoresponsivity. This limits the ability to achieve high‐performance nonvolatile photoresponsivity. Yue et al.^[^ ^68^ ^]^ used a retinomorphic PV device to overcome this limitation and achieve highly reconfigurable, nonvolatile photoresponsivity by utilizing BPVE of rhombohedral (3R) stacked/interlayer sliding WS_2_. **Figure** **13**a illustrates the long‐wavelength (L) cones for red (R) perception, the medium‐wavelength (M) cones for green (G), and the short‐wavelength (S) cones for blue (B) perception.^[^ ^126^, ^127^ ^]^ The internal electric fields of these materials can be shifted by changing their stable ferroelectric polarization states. This modification improves the tunability of photoresponsivity and performance beyond conventional bounds by influencing the effectiveness of charge carrier separation and collection. 2D ferroelectrics with tunable thickness enable reconfigurable and nonvolatile photoresponsivity and are ideal for generating more photocurrent through BPVE. This highlights the development of in‐sensor computing systems for machine vision (Figure 13a). A retinomorphic device structure was created using a 2D all vdWs layered Gr/3R‐WS_2_/Gr (Figure 13b) configuration in order to illustrate the BPVE concept for in‐sensor computing, which is depicted in Figure 13a. In this setup, direct measurement of photogenerated carriers and electrically controlled polarization are made possible by the high transmittance of Gr electrodes in the visible range. These devices were created by vertically stacking 2D 3R‐WS_2_ with Gr electrodes using mechanical exfoliation and a controlled dry transfer technique; the corresponding optical microscopy image is displayed in the inset of Figure 13b.

The 3R‐WS_2_‐based retinomorphic device's ability to generate the PV effect under uniform light illumination was investigated. Under uniform illumination (wavelength: 623 nm, light intensity: 20 mW cm^−2^), as illustrated in Figure 13c, the device's *I*–*V* characteristics clearly demonstrate a PV effect, with a positive I_SC_ of 0.95 nA and a negative V_OC_ = −78 mV. It is possible to switch both I_SC_ and V_OC_ efficiently by applying poling voltages in opposite directions. The Gr/3R‐WS_2_/Gr structure's switchable energy band alignments, as shown in Figure 13d, provide a physical explanation for the polarization‐controlled PV behavior. The Fermi level of Gr can be shifted by the polarization of ferroelectric materials, which can modulate the asymmetric band structure and result in asymmetric barrier changes. The Fermi level of the top Gr electrode rises and lowers the contact barrier height when the polarization points upward, while the Fermi level of the bottom Gr electrode moves downward and raises the contact barrier height. This produces a built‐in, downward electric field (represented by the blue arrow), which facilitates the separation and movement of photogenerated carriers in the presence of light. Consequently, the photocurrent decreases due to electron build‐up on the top Gr electrode. On the other hand, the inherent electric field reverses direction when the polarization points downward. As a result, more holes form on the top Gr, increasing the photocurrent. Therefore, the ferroelectric polarization state controls the strength of the built‐in electric field, which in turn determines the photocurrent magnitude. The Gr electrode exhibits a Fermi level shift of roughly 0.35 eV, which is indicative of ferroelectric polarization switching in 3R‐WS_2_. This provides direct proof of polarization‐modulated electronic properties at the Gr/WS_2_ interface. The reconfigurable 3R‐WS_2_ ferroelectric PV retinomorphic devices with in‐sensor computing capabilities can effectively enable target recognition and low‐power machine vision in autonomous robotic chips.

## 10 Polarization Effects on 2D Materials

Polarization is crucial for inducing the BPVE effect in 2D materials. Spontaneous polarization in 2D ferroelectrics such as CIPS and α‐In_2_Se_3_ enables BPVE by separating photoexcited carriers under illumination above the bandgap and producing a shift current in the direction of the polarization.^[^ ^14^, ^42^, ^128^ ^]^ The polarization can be switched with an external electric field for control and reversal of the direction and magnitude of this photocurrent.^[^ ^42^ ^]^ In addition to their visible light absorption and tunable polarization enabling high photocurrent densities and efficient solar energy conversion, ultrathin 2D materials' strong ferroelectric polarization frequently results in a BPVE significantly larger than in their bulk counterparts, making them promising candidates for advanced PV applications.

### 10.1 Single Polarization Effect

In 2D materials, spontaneous polarization that occurs only in one direction, either in‐plane or out‐of‐plane, is referred to as a single polarization effect. This breaks inversion symmetry and makes BPVE possible. The photocurrent produced by BPVE in 2D ferroelectrics with single in‐plane polarization is usually robust but non‐switchable because material symmetry constraints prevent the in‐plane polarization from being reversed.^[^ ^61^, ^125^ ^]^ On the other hand, materials with a single out‐of‐plane polarization enable switchable BPVE, which is ideal for tunable photoelectric applications because it reverses the direction of the polarization and, in turn, the direction of the photocurrent when an external electric field is applied.^[^ ^125^ ^]^ This single‐directional polarization is essential because it enables effective light‐to‐electricity conversion without p‐n junctions, provides the symmetry breaking required for BPVE, and allows control over photocurrent characteristics through the engineering of material symmetry and polarization direction.^[^ ^42^, ^61^, ^67^ ^]^

**Figure 14:**
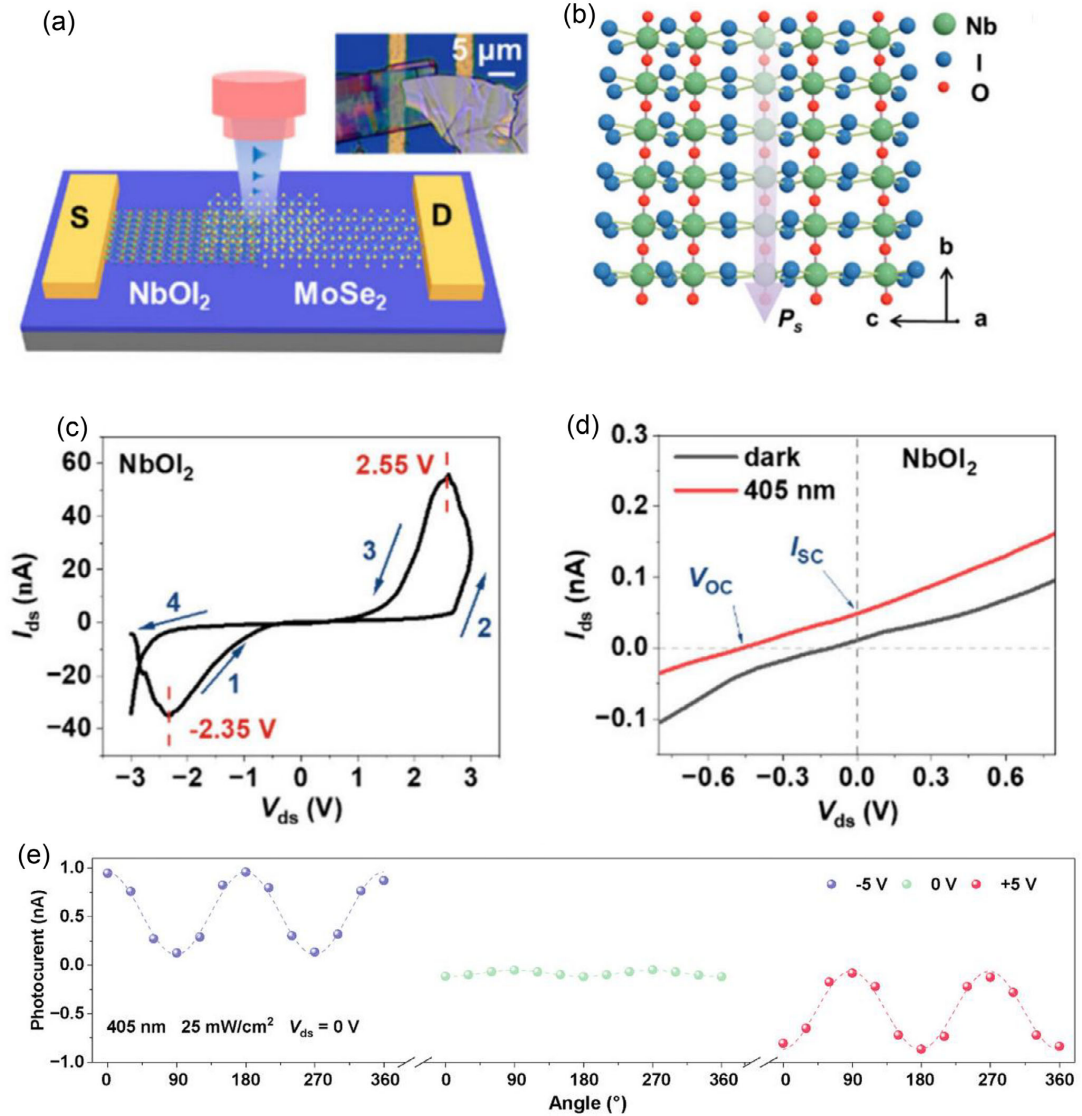
a) Schematic of a MoSe_2_/NbOI_2_ heterojunction‐based self‐powered photodetector. An optical micrograph of the apparatus is displayed in the inset. b) NbOI_2_ crystal structure. The spontaneous polarization direction is indicated by the purple arrow. c) NbOI_2_ device's *I*–*V* hysteresis curve in the dark along its *b*‐axis. d) NbOI_2_ device's *I*–*V* characteristics under 405 nm light (800 mW cm^−2^) and in the dark. e) Photocurrent at 0 V bias as a function of polarization angle. (a–e) Reproduced with permission.^[^ ^67^ ^]^ Copyright 2025, ACS.

Although 2D ferroelectric materials have the potential to function as self‐powered photodetectors due to their ability to exhibit BPVE, their practical application is frequently limited by weak photoresponse, which is primarily caused by low optical transition strength and naturally wide bandgaps. Xiong et al.^[^ ^67^ ^]^ combined a highly light‐absorbing MoSe_2_ layer with NbOI_2_, which exhibits strong in‐plane polarization, to create a vdWs heterojunction that improved PV performance. As shown in **Figure** **14**a, mechanical exfoliation and a dry transfer technique were used to fabricate the MoSe_2_/NbOI_2_ photodetector. As shown in Figure 14b, NbOI_2_ has a typical vdWs layered structure with lattice parameters a = 7.52 Å, b = 3.924 Å, and c = 15.036 Å. It is a member of the monoclinic space group *C*2.^[^ ^129^ ^]^ The dimerization of Nb atoms caused by the first‐order Peierls distortion along the c‐axis results in alternating Nb–Nb bond lengths. Meanwhile, the Nb atoms at the octahedron's center shift considerably toward the oxygen atoms due to the second‐order Peierls distortion along the b‐axis, producing a strong spontaneous in‐plane ferroelectric polarization.^[^ ^130^ ^]^ The *I*–*V* characteristics of the material measured along the b‐axis at room temperature in Figure 14c. BPVE is characterized by a direction‐dependent photoresponse, as shown in Figure 14d, where a positive I_SC_ and a negative V_OC_ are generated along the *b*‐axis but not along the *c*‐axis.^[^ ^42^, ^131^ ^]^ The production of non‐zero V_OC_ and I_SC_ through BPVE is generally ascribed to either a real‐space shift through the ballistic transport of nonequilibrium carriers or an asymmetric momentum distribution of nonthermalized (hot) carriers, both of which are caused by the absence of inversion symmetry in the material.^[^ ^43^, ^132^ ^]^ It is believed that the photoresponse associated with incident light polarization is an inherent feature of BPVE.^[^ ^133^ ^]^ As shown in Figure 14e, the heterojunction device and the NbOI_2_‐only device both displayed polarity‐switchable polarization sensitivity in this situation, which is consistent with their ambipolar photoresponse behavior. This approach suggests new strategies for the development of upcoming self‐powered devices and deepens our understanding of self‐powering mechanisms fueled by BPVE.

### 10.2 Dual Polarization Effect

Dual polarization mechanisms that exploit interfacial and ferroelectric properties can significantly enhance BPVE in 2D materials, paving the way for efficiency beyond traditional limits. When Gr and ultrathin ferroelectric CIPS are combined in vdW heterostructures, the result is an inherent electric field that allows for ultrafast charge separation and high photocurrent densities (≈100 nA·µm^−2^) without the need for external bias.^[^ ^42^, ^66^ ^]^ Room temperature operation and electric field‐controlled photocurrent direction are made possible by ferroelectric polarization in 2D materials like CIPS, which is driven by off‐center Cu ions. Its performance is two orders of magnitude better than that of 3D ferroelectrics.^[^ ^42^ ^]^ Efficiency is improved by the dimensional crossover at ≈40 nm thickness because of increased Berry‐phase effects and reduced recombination.^[^ ^42^ ^]^

**Figure 15:**
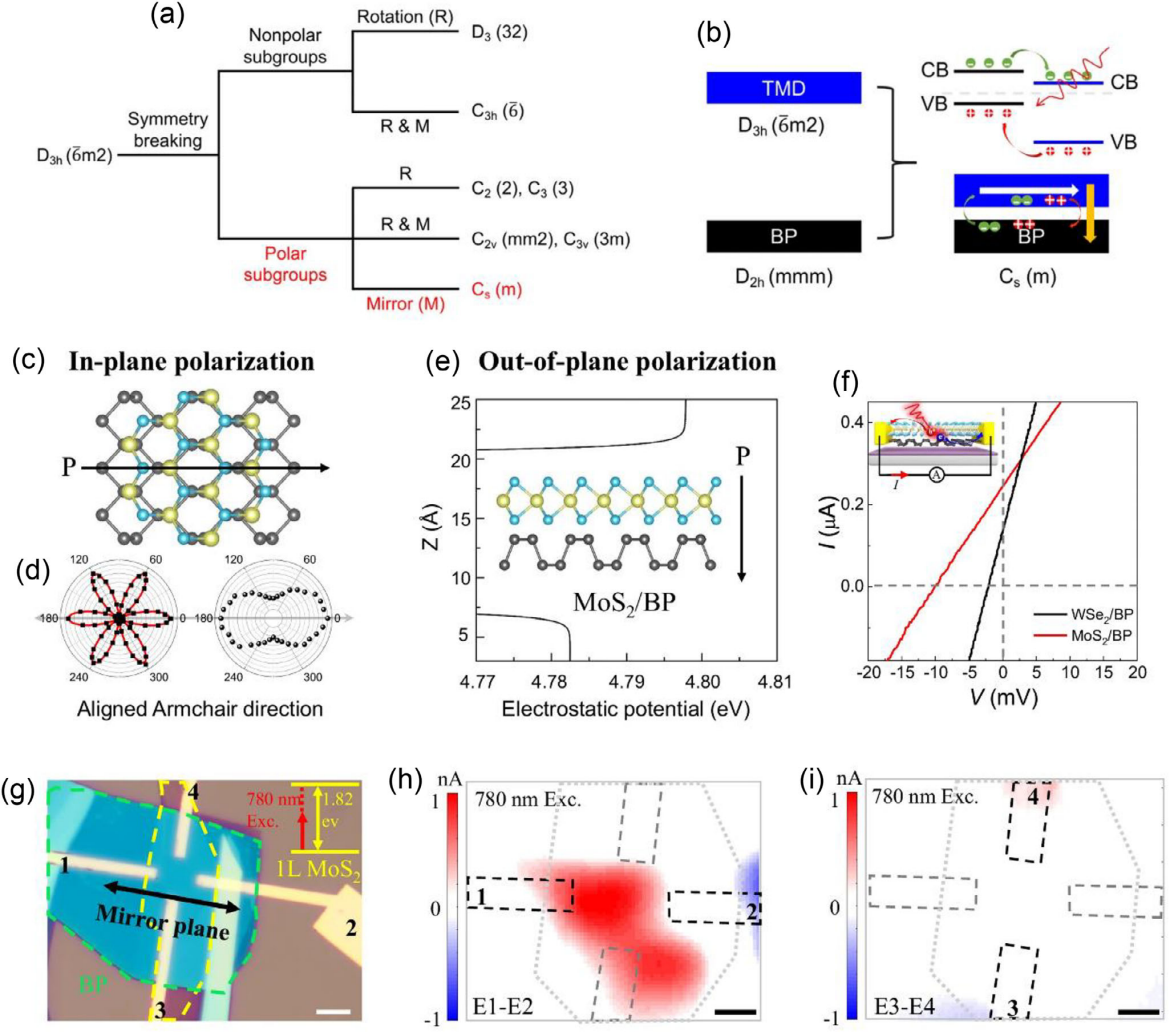
a) TMD monolayer symmetry breaking. For BPVE, the D_3h_ symmetry is broken to form five polar groups. b) The TMD monolayer is stacked on top of a BP monolayer to produce polar symmetry Cs. The white and yellow arrows, respectively, indicate the generated in‐plane and out‐of‐plane polarizations. Charge carrier transfer in VB and CB is indicated by the red and green arrows, respectively. c) Diagrammatic representation of the armchair direction‐aligned MoS_2_/BP heterostructure's in‐plane polarization generation (shown by the black arrow). d) Raman intensity ratio of the BP peak (right panel) and SHG polarization in monolayer MoS_2_ (left panel) are shown. The gray arrow in the right panel denotes the polar direction, which defines the armchair direction in the two distinct materials, respectively. e) Simulation of the armchair direction‐aligned MoS_2_/BP heterostructure's planar‐averaged electrostatic potential along the z‐direction, indicating the formation of out‐of‐plane polarization (shown by the black arrow). The schematic side view of the monolayer MoS_2_/BP heterostructure is displayed in the inset. f) Photocurrent comparison between WSe_2_/BP (black) and MoS_2_/BP (red) devices (P = 71.3 mW cm^−^^2^, λ = 633 nm).The TMD/BP BPV device's schematic illustration is displayed in the inset. g) An optical picture of the MoS_2_/BP BPV device with two electrode pairs, where electrodes E1–E2 are parallel to the device's mirror plane and electrodes E3–E4 are perpendicular. The monolayer MoS_2_ and BP are delineated by the yellow and green curves, respectively. The excitation at 780 nm (1.589 eV) is below the MoS_2_ bandgap (1.82 eV), as the inset illustrates (white scale bar is 7 µm). Images from a scanning photocurrent microscope using a 780 nm laser and electrodes h) E1–E2 and i) E3–E4. The heterostructure's overlap area is indicated by the dashed gray lines (scale bar is 4 µm). (a–i) Reproduced under the terms of the CC‐BY 4.0 license.^[^ ^66^ ^]^ Copyright 2024, Zeng et al.

New vdWs heterostructures provide an ideal platform for BPVE because interfacial interactions naturally cause charge polarizations by disrupting the crystal symmetries of the constituent parts. Zeng et al.^[^ ^66^ ^]^ used dual interfacial polarizations in vdW heterostructures to show how to achieve ultrafast BPVE. Broken inversion symmetry, which can be introduced by polar groups, is necessary for the observation of BPVE. TMD monolayers can produce five different types of polar groups by breaking the D3h symmetry, as shown in **Figure** **15**a. C2, C2v, and Cs can induce in‐plane polarization. This process is facilitated for the TMD/BP heterostructure by the type‐II band alignment and the intrinsic electric field that is directed from the TMD layer to BP (Figure 15b). To prepare MoS_2_/BP heterostructures, a MoS_2_ monolayer was carefully stacked onto a BP flake with aligned armchair directions (Figure 15c). The crystal orientations were then determined (Supplementary Note 2) using the Raman intensity ratio in BP (Figure 15d, right panel) and second harmonic generation (SHG) polarization in the MoS_2_ monolayer (Figure 15d, left panel). The out‐of‐plane polarization strength was found to be on the order of tens of microvolts by the planar‐averaged electrostatic potential simulation along the z‐direction (Figure 15e). The MoS_2_/BP device shows an I_SC_ of ≈250 nA when illuminated with a 633 nm continuous wave (CW) laser, which is double that of the WSe_2_/BP device (Figure 15f). Scanning photocurrent microscope (SPCM) measurements were performed without the use of an external bias in order to ascertain whether the photoresponse under 780 nm (1.589 eV) illumination originated from BPVE. Two pairs of crossed electrodes were positioned parallel (E1–E2) and perpendicular (E3–E4) to the mirror plane, respectively, in a different MoS_2_/BP device to better demonstrate photocurrent generation (Figure 15g). A uniformly polarized photocurrent spread across the heterostructure region when the E1–E2 electrodes were connected, and the intensity of the photocurrent rose away from the electrodes (Figure 15h), suggesting spontaneous charge carrier separation fueled by in‐plane polarization. Conversely, the photothermoelectric effect was responsible for the weak photocurrent with different polarities that was produced close to the electrode regions when the E3–E4 electrodes were connected (Figure 15i).^[^ ^134^ ^]^ The experimentally observed intrinsic BPVE response time in MoS_2_/BP heterostructures reaches 26 ps, which is orders of magnitude faster than in traditional non‐centrosymmetric materials, demonstrating the idea. Moreover, the heterostructure device exhibits one of the highest reported bulk PV coefficients for vdW BPV devices to date (0.6 V^−1^) and an extrinsic response time of ≈2.2 ns. This study demonstrates a successful method for creating ultrafast BPVE for high‐speed photodetection.

## 11 Depolarization Field Effect

BPVE in ferroelectric and 2D materials, particularly 2D systems, is modulated in large part by the depolarization field. In materials such as α‐In_2_Se_3_ and CIPS, a strong depolarization field created by incomplete screening of spontaneous polarization promotes asymmetric carrier separation and permits the generation of photocurrents without the need for an external bias.^[^ ^42^, ^65^, ^128^, ^135^ ^]^ As demonstrated by ion migration in CIPS, this field can even reverse photocurrent polarity regardless of the direction of polarization.^[^ ^136^ ^]^ Depolarization effects in vdW ferroelectrics are amplified by reduced thickness, and dual interfacial polarizations are used in engineered heterostructures such as MoS_2_/BP to improve both in‐plane and out‐of‐plane charge transport.^[^ ^42^, ^66^, ^137^ ^]^ Electrode design also affects depolarization dynamics; edge‐contact configurations enhance carrier mobility and strain distribution, while symmetrical setups isolate intrinsic BPVE. Additional factors, such as tensile strain in 3R‐MoS_2_, have been shown to tune depolarization fields and produce record‐high BPVE coefficients.^[^ ^48^ ^]^ In‐plane polarization in MoS_2_/BP heterostructures is confirmed to align with photoexcited carrier asymmetry through theoretical studies using DFT, leading to ultrafast BPVE responses (≈26 ps).^[^ ^66^ ^]^ These results demonstrate that the depolarization field is a controllable and advantageous characteristic rather than a drawback, with significant ramifications for the design of high‐efficiency nanoelectronic and PV devices.

**Figure 16:**
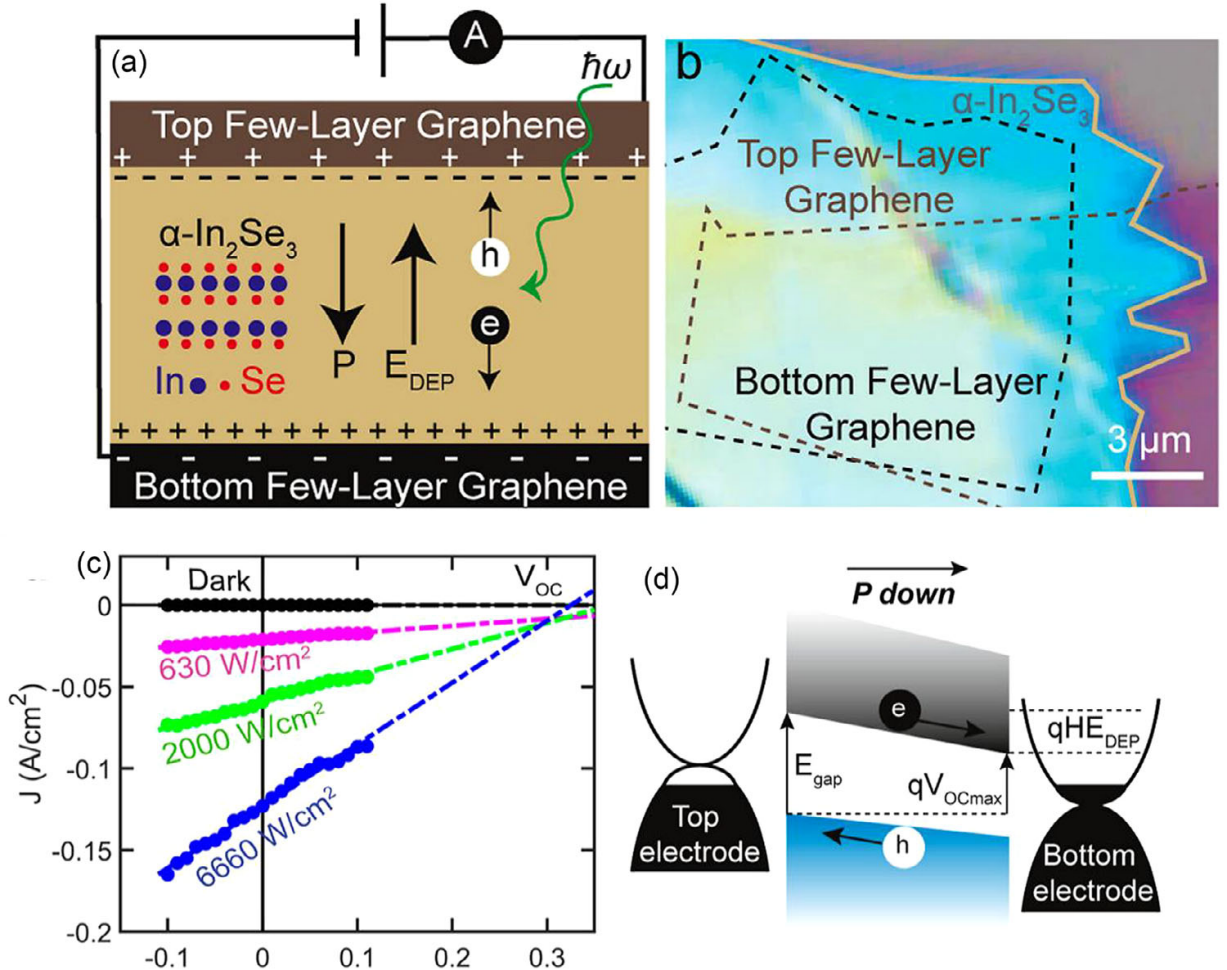
a) Device architecture and depolarization field‐induced FPVE schematic diagram. The crystal structure of α‐In_2_Se_3_ with down polarization along the ⟨1̅100⟩ axis is displayed in the inset in (a). Induced charges in the few‐layer Gr electrodes are represented by the white + and − signs, whereas the black + and − signs in the α‐In_2_Se_3_ layer indicate bound charges because of polarization. A current flow that is antiparallel to the polarization is produced by a depolarization field, which is caused by incomplete screening of the bound charges. b) Optical representation of a standard FPVE apparatus. The top few‐layer Gr, α‐In_2_Se_3_, and bottom few‐layer Gr are marked in brown, tan, and black, respectively, and the FPVE devices are made up of few‐layer Gr /α‐In_2_Se_3_/few‐layer Gr heterostructures. c) *J*–*V* output curves at a small bias (±0.1 V) with increasing laser intensity. The curves' linear fits are shown by the dashed lines. d) Band diagram of the heterostructure. The depolarization field, which is oriented against the direction of polarization, induces the slope and produces the photocurrent. The top and bottom few‐layer Gr electrodes receive opposite hole and electron doping from the ferroelectric's interfacial polarization charges, respectively. (a–d) Reproduced with permission.^[^ ^65^ ^]^ Copyright 2024, ACS.

The ferroelectric PV effect (FPVE) takes advantage of symmetry‐breaking in non‐centrosymmetric materials to provide energy conversion pathways that are not permitted in centrosymmetric systems. To fully realize the efficiency potential of FPVE, it is imperative to comprehend the dominant mechanism at the ultrathin limit. 2D α‐In_2_Se_3_ is a perfect candidate for creating ultrathin, stable heterostructures because of its remarkable structural stability and optimal bandgap of 1.3 eV, which sets it apart from conventional wide bandgap thin‐film ferroelectrics. This unique combination enables the investigation of FPVE at the nanoscale, paving the way for highly efficient optoelectronic devices of the future. In vertical few‐layer Gr /α‐In_2_Se_3_/ Gr heterostructures, Nahid et al.^[^ ^65^ ^]^ examined the thickness‐dependent FPVE. According to their research, the short‐circuit photocurrent increases exponentially with decreasing ferroelectric layer thickness and is antiparallel to the ferroelectric polarization. This underscores the crucial role that dimensional scaling plays in improving FPVE performance at the nanoscale. The FPVE device architecture schematic is shown in **Figure** **16**a, emphasizing the physical mechanism that is fueled by the depolarization field. This configuration establishes α‐In_2_Se_3_ as a room‐temperature 2D ferroelectric semiconductor that is suited for effective nanoscale PV applications owing to its ideal bandgap of 1.3 eV and out‐of‐plane polarization.^[^ ^138^, ^139^, ^140^ ^]^ The α‐In_2_Se_3_ channel, which has a thickness of 18–50 nm, is positioned between two overlapping transparent few‐layer Gr electrodes (2–8 nm thick) in an out‐of‐plane device geometry. In addition to facilitating efficient charge collection, this vertical arrangement emphasizes how the depolarization field drives the PV response. The crystal structure of α‐In_2_Se_3_ is shown in the inset of Figure 16a, highlighting its layered structure and inherent out‐of‐plane ferroelectric polarization. The α‐In_2_Se_3_ layer thickness was systematically varied from 18 to 50 nm across five few‐layer Gr /α‐In_2_Se_3_/few‐layer Gr heterostructure devices (labeled #1 to #5). A controlled investigation of thickness‐dependent ferroelectric PV behavior was made possible by the transparent few‐layer Gr electrodes utilized in these devices, which had a thickness ranging from 2 to 8 nm, or roughly 6 to 24 Gr layers. An optical image of device #2, for instance, with a 25 nm‐thick α‐In_2_Se_3_ layer, is shown in Figure 16b. This image shows the typical structure and size of the manufactured heterostructures. To examine the impact of laser intensity on the photoresponse, the diffraction‐limited laser spot was directed toward the area of maximum I_SC_. A distinct intensity‐dependent improvement in the device's PV performance is observed when the photocurrent density is plotted as a function of bias voltage under various laser intensities, as seen in Figure 16c. The suggested band diagram, which shows how the depolarization field affects band energies and promotes the generation of photocurrent, is shown in Figure 16d. The out‐of‐plane depolarization field in α‐In_2_Se_3_ tilts the band edges under laser illumination, allowing photogenerated carriers to be separated spatially. When the ferroelectric polarization points downward, the energy barrier for electrons at the bottom electrode is lower than that at the top electrode. A photocurrent that is antiparallel to the direction of polarization is produced as a result of the preferential collection of electrons at the bottom electrode and holes at the top. The maximum theoretical open‐circuit voltage is corresponds to the potential difference between the valence band edge at the top interface and the conduction band edge at the bottom interface before any recombination takes place, assuming that the photoexcited carrier concentration is much higher than the intrinsic carrier concentration.^[^ ^141^, ^142^ ^]^ These findings lay the groundwork for the development of next‐generation ultrathin and highly efficient optoelectronic devices by highlighting the crucial role of the depolarization field at the nanoscale and establishing fundamental design principles for efficiently utilizing the FPVE in reduced‐dimensional systems.

## 12 Edge Contact Effect

In 2D TMDs like 3R‐MoS_2_, the edge contact (EC) configuration greatly improves BPVE by maximizing electrode‐material interactions, strain, and polarization access. In contrast to top contacts (TC), which block deeper layers, EC devices geometrically offer lateral polarization access, enabling light to pass through and interact with the in‐plane polarization across all underlying layers of 3R‐MoS_2_.^[^ ^48^ ^]^ This optimizes carrier generation and light‐matter interaction. Furthermore, EC electrodes, which are frequently composed of bismuth, adhere firmly to the edges of the material and cause interfacial bonding and thermal expansion mismatch, which results in tensile strain of ≈0.14%.^[^ ^48^ ^]^ This strain is verified by Raman spectroscopy using a 0.24 cm^−1^ redshift in the $E_{2g}^{1}$ phonon mode, which intensifies the structural asymmetry that is essential for BPVE.^[^ ^143^ ^]^ The photocurrent of both EC and TC devices shows a cosine dependence on laser polarization; however, because strain‐mediated symmetry breaking is more effective in EC devices, the photocurrent magnitude is significantly larger. In terms of electrode composition, bismuth semimetal electrodes minimize carrier recombination losses by reducing Fermi‐level pinning and offering superior Ohmic contacts in contrast to traditional metals like Cr/Au. Photocurrents in devices with Bi/Au EC electrodes are about ten times greater than those in devices with Cr/Au EC electrodes. The SQ efficiency limit may also be overcome by hybrid systems such as 3R‐MoS_2_/WSe_2_ heterojunctions, which combine BPVE with traditional PV effects to improve photodetection and energy harvesting capabilities. TMDs are promising candidates for high‐efficiency optoelectronic applications since the edge contact geometry improves BPVE through better electrode interfaces, enhanced geometric strain engineering, and full polarization exploitation.

**Figure 17:**
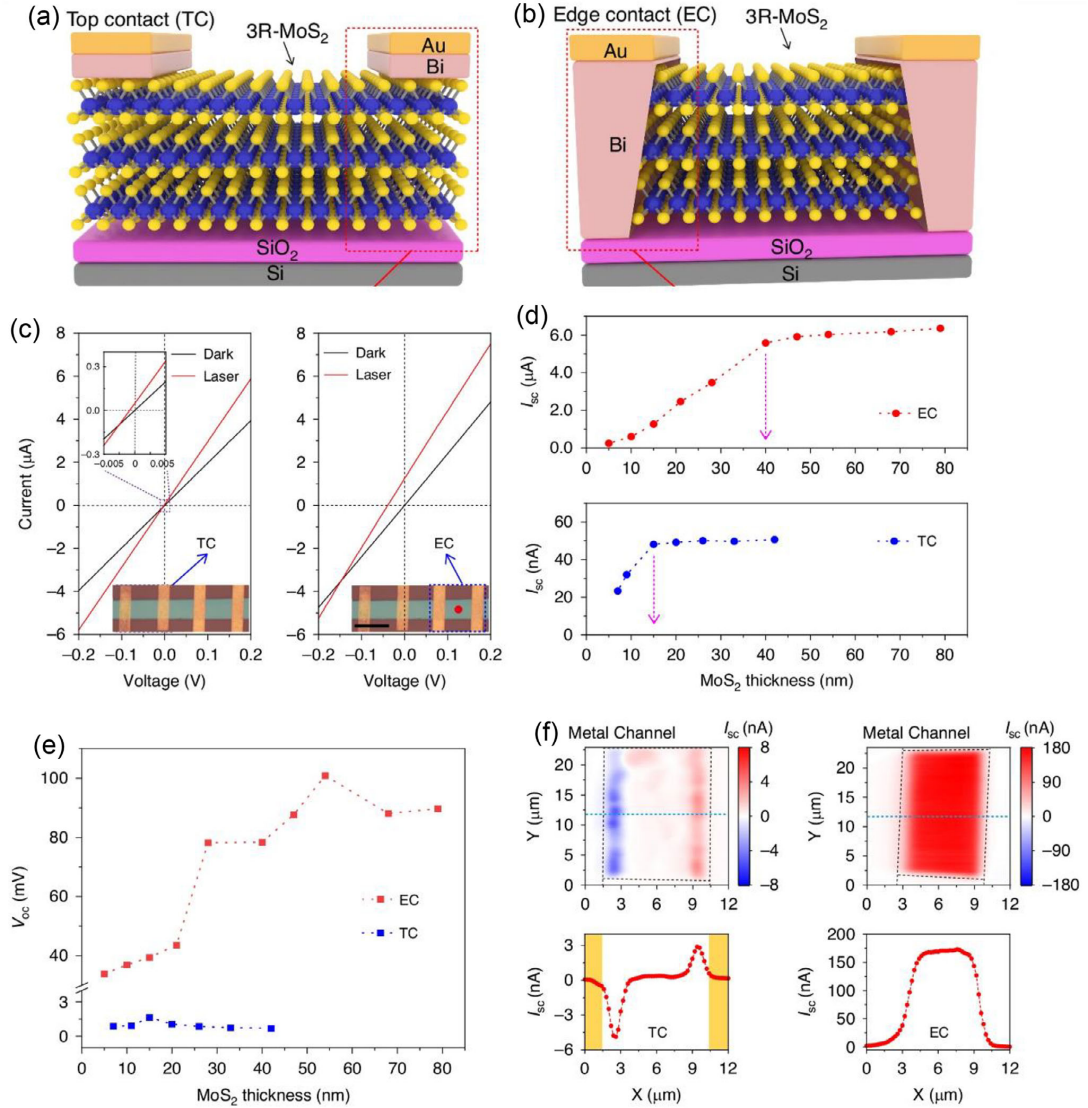
a) Schematic of the TC device. b) Schematic of the EC device. c) TC and EC 3R‐MoS_2_ devices' *I*–*V* curves under a 488 nm laser (395 µW). Inset: Red dots indicate the locations of laser illumination in optical micrographs of devices (scale bar is 10 µm). BPVE PV d) and photocurrent e) dependence on the thickness of the 3R‐MoS_2_ layer for TC and EC devices. f) Under a 532 nm laser (3 µW), spatial photocurrent mapping of TC and EC devices and I_SC_ variation along light‐blue dotted lines. (a–f) Reproduced under the terms of the CC‐BY 4.0 license.^[^ ^48^ ^]^ Copyright 2025, Qiaoet et al.

Although their cell efficiency is low, oxide materials with non‐centrosymmetric structures display BPVE. Recently, 2D layers and stacks with asymmetry‐induced spontaneous polarization exhibit comparatively higher BPVE coefficients. In 3R‐MoS_2_, Qiao et al.^[^ ^48^ ^]^ report a notable improvement in BPVE enhancement through the use of EC geometry with a bismuth semimetal electrode. The EC metal, which firmly sticks to the edges and substrates, causes noticeable tensile strain in the 3R‐MoS_2_ in contrast to the widely used TC geometry. Furthermore, the lateral contact geometry provides complete access to in‐plane polarization from the light‐exposed underlying layers, which boosts the BPVE photocurrent by more than 100 times. Two device types were fabricated (**Figure** **17**a,b) to examine the difference in BPVE between TC and EC configurations. In these devices, exfoliated 3R‐MoS_2_ flakes were transferred onto the SiO_2_/Si substrate using polydimethylsiloxane (PDMS). The impact of contact style on the BPVE of 3R‐MoS_2_ was investigated by measuring *I*–*V* curves in both laser and dark illumination settings (Figure 17c). The illumination source was a linearly polarized laser (488 nm) with a focused beam diameter of ≈1.1 µm. The polarization orientation was aligned with the device's channel direction during the measurements. The dark current values of TC and EC devices are comparable when the environment is dark. Under illumination, however, a notable variation in the BPVE response is noted. The TC device displays an open‐circuit PV of 1.65 mV and a BPVE short‐circuit photocurrent of 48.11 nA when illuminated with 395 µW of power. A significantly higher I_SC_ of 1.26 µA and V_OC_ of 39.44 mV are displayed by the EC device, in comparison. This noticeable difference suggests that the EC configuration significantly improves BPVE when compared to the TC configuration. Figure 17d,e provides a summary of the I_SC_ and V_OC_ results at an incident power of 395 µW. As the thickness of the 3R‐MoS_2_ increases from 5 to 40 nm, I_SC_ in the EC device increases rapidly and linearly before approaching saturation.

To visually distinguish these differences, measurements of the photocurrent spatial distribution were made for both TC and EC devices. A concentrated laser spot was scanned over active regions of the devices to achieve this (Figure 17f). The presence of BPVE in 3R‐MoS_2_ was confirmed by the observation of a positively low I_SC_ of ≈0.5 nA in the TC device, which was distributed nearly evenly throughout the channel region. Furthermore, comparatively greater photocurrents with antisymmetric polarity were found close to the two electrodes; these were ascribed to either the photothermal effect or the existence of a tiny Schottky barrier between the metal and 3R‐MoS_2_. This finding is consistent with prior research showing that the Schottky barrier effect is usually stronger than BPVE.^[^ ^70^, ^72^ ^]^ In stark contrast, the EC device shows no mapping signatures of Schottky barriers close to the electrodes and a uniformly distributed high I_SC_ of ≈171.2 nA throughout the device area. The depinning effect and the existence of a clean edge contact between 3R‐MoS_2_ and Bi are further supported by this observation.^[^ ^144^ ^]^ In the designed heterojunction solar cell device, BPVE makes a substantial contribution. To the best of our knowledge, the coupling of these two effects in TMDs has not been previously observed, which could lead to a number of applications.

## 13 Challenges and Future Perspectives

### 13.1 Engineered Phase Stability in Advanced Materials

Phase transitions, such as 2H to 1T′ or 3R, are essential for adjusting electronic properties in materials like TMDs, but they are frequently hindered by metastability. Environmental sensitivity to elements such as temperature changes, exposure to oxygen or water, and electric fields—which can result in reversion to stable phases—are major challenges. Degradation is also caused by thermodynamic instability, such as 1T′‐MoS_2_ existing in local energy minima, which affects the dependability of devices in FETs, batteries, and optoelectronics. Encapsulation techniques employing materials such as Gr, hexagonal boron nitride (h‐BN), or oxide coatings (e.g., AlO_3_, HfO_2_) can improve stability by protecting against ambient degradation. Through interlayer coupling, substrate engineering aids in the stabilization of phases, such as 3R‐WS_2_/WSe_2_. This includes strain modulation and the construction of vdWs heterostructures. Phase stability is shifted by chemical methods such as covalent functionalization, alloying (e.g., Mo_1_₋_x_W_x_S_2_), and doping or intercalation with Li⁺ or thiols. Phase retention is further improved by such thermodynamic techniques as phase seeding and defect engineering. In situ characterization using TEM and Raman spectroscopy, machine learning‐guided material optimization, combining several stabilization techniques, and creating scalable, roll‐to‐roll manufacturing methods should be the main priorities of future research. Important considerations include striking a balance between cost and performance and confirming stability through prolonged testing under demanding circumstances. Advancing these methods is essential for developing robust, phase‐engineered materials for next‐generation technologies.

### 13.2 Integration into Scalable Devices

Significant challenges remain in integrating BPVE devices into large‐area, flexible, and manufacturing platforms, especially those based on phase‐ and structure‐engineered 2D TMDs. There are still a number of technical obstacles to scalable integration, despite the fact that BPVE has been demonstrated in lab settings.^[^ ^66^, ^145^, ^146^ ^]^ Achieving uniformity and reproducibility across large areas is a major challenge, as variations in material properties, synthesis techniques, thickness, composition, and defect density can impede consistent device performance.^[^ ^145^ ^]^ Another challenge is compatibility with current semiconductor manufacturing processes, which call for 2D TMDs to work with conventional lithography, deposition, and etching methods while supporting flexible or unusual substrates.^[^ ^145^, ^147^ ^]^ Although scalable fabrication techniques like transfer printing and CVD have promise, they still require improvement in terms of yield, reproducibility, and industrial compatibility.^[^ ^145^, ^146^ ^]^ For complex device architectures, solutions like improvements in CVD synthesis and transfer printing enable accurate layer placement and stacking. Techniques for layer‐by‐layer assembly provide more control over the creation of heterostructures, allowing for customized optical and electrical characteristics for high‐performance and multipurpose applications. Developing strong integration protocols, improving material quality, and standardizing processes are all necessary to ensure reliability and manufacturability at scale. In conclusion, BPVE in engineered 2D TMDs has considerable promise for next‐generation optoelectronics, but overcoming challenges with fabrication, homogeneity, and integration with existing manufacturing technologies will be necessary to achieve large‐scale deployment.

### 13.3 Co‐Engineering Phase, Structure, and Polarization (Synergistic Effects)

In advanced optoelectronic materials research, the co‐engineering of phase, structure, and polarization to produce synergistic enhancements in BPVE is a promising avenue. The combined approach could unlock higher performance by enabling multimodal symmetry breaking and enhanced electric field gradients, whereas most prior research has concentrated on manipulating these factors separately, such as tuning crystal phase transitions, engineering structural defects or interfaces, and controlling spontaneous or interfacial polarization.^[^ ^12^, ^39^, ^66^ ^]^ In order for BPVE to work, inversion symmetry must be broken, which can be done by adding polar groups, changing the structure, or undergoing phase transitions.^[^ ^13^, ^39^ ^]^ Materials can display more intricate and adjustable symmetry breaking by co‐engineering these features, which could result in more robust and manageable BPVE responses. Furthermore, steeper or more localized built‐in electric fields can be produced by combining structural engineering (such as strain, domain walls, or heterostructures) with polarization control. This enhances charge separation and extraction, which is essential for BPVE efficiency. However, there are highly nonlinear and interdependent interactions between phase, structure, and polarization; for instance, structural strain can change polarization or cause phase transitions, while interfaces or domain walls may serve as locations for increased charge transport and generation. To fully realize this co‐engineering potential, sophisticated multi‐physics modeling that combines electrostatics, thermodynamics, and electronic structure is necessary to forecast the best combinations and direct experiments. Furthermore, dynamic changes in these properties under operating conditions can be monitored using real‐time in situ characterization techniques like electron microscopy, optical spectroscopy, and X‐ray diffraction, which connect BPVE enhancements to material modifications. Machine learning can further accelerate discovery by navigating the vast design space and predicting promising co‐engineering pathways using computational and experimental data. In concert, structural changes can produce built‐in fields and improve transport by generating localized field gradients, phase transitions can change crystal symmetry to allow for new symmetry‐breaking modes, and polarization effects can promote charge separation while intensifying field effects for better charge extraction. To summarize, the co‐engineering phase, structure, and polarization provide a potent method for enhancing BPVE synergistically; however, in order to optimize materials in the face of intricate interdependencies, it necessitates integrated modeling, sophisticated characterization, and machine learning.

### 13.4 Twisted vdW Systems for Future BPVE Applications

**Figure 18:**
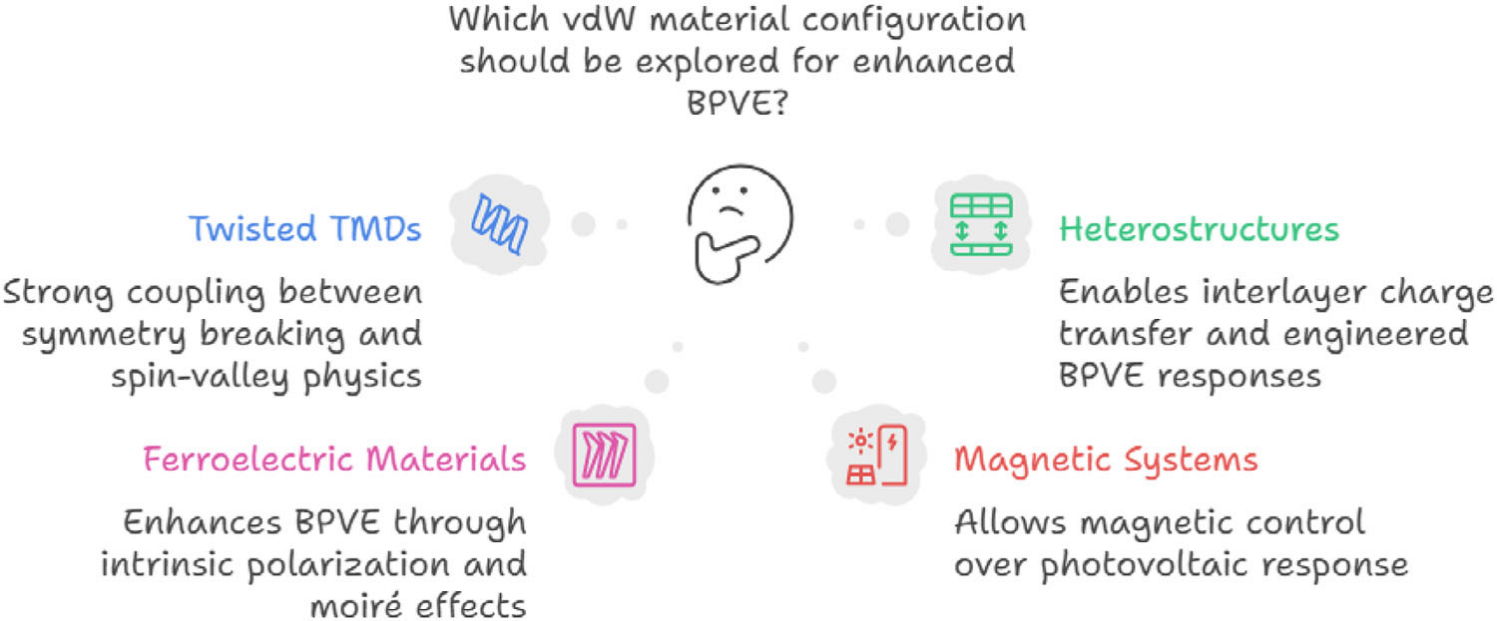
Twisted vdW materials for enhancing BPVE for optoelectronic applications.

Beyond BP, a variety of twisted vdW systems can be explored using BPVE in future research. Twisted TMD, like MoS_2_ and WS_2_, offer promising platforms due to their tunable symmetry and strong spin–valley coupling.^[^ ^148^ ^]^ Heterostructures composed of different 2D materials, like BP/MoS_2_, may enable tailored interlayer interactions and enhanced BPVE responses. While adding magnetic layers like CrI_3_ may result in spin‐polarized BPVE, enabling magnetic control of the PV response, twisting ferroelectric vdW materials such as In_2_Se_3_ could couple intrinsic polarization with Moiré‐induced effects. There is a great deal of promise in these directions for creating energy‐harvesting and tunable, highly efficient optoelectronic devices (**Figure** **18**).

### 13.5 Advancing 2D Ferroelectrics for Next‐Gen Photovoltaics and Optoelectronics

Nevertheless, key challenges remain in scaling the synthesis of ultrathin ferroelectrics below 40 nm while preserving their structural and functional integrity, optimizing integration with conductive electrodes like Gr to reduce interface losses, and discovering novel 2D ferroelectrics that combine narrow bandgaps, high polarization, and environmental stability. Collectively, these developments highlight the transformative potential of 2D ferroelectrics in next‐generation solar energy harvesting, optoelectronics, and neuromorphic technologies. The sensitivity of BPVE to light polarization also provides benefits for the design of innovative optoelectronic devices. Despite its theoretical potential, BPVE has many real‐world obstacles. These challenges include high recombination rates and low charge carrier mobility, which at the moment lead to lower device efficiencies when compared to traditional PVs. Ongoing research, however, aims to overcome these constraints by improving the design of materials, learning more about the physics at play, and discovering new compounds with improved BPVE responses.

### 13.6 Forecast for BPVE‐Based 2D Optoelectronic and Energy‐Harvesting Technologies

Thanks to recent advancements in materials engineering and device design, the future of BPVE‐based 2D optoelectronic and energy‐harvesting technologies appears highly promising. Notably, when combined with edge‐contact bismuth electrodes, 2D TMDs such as 3R‐MoS_2_ have enhanced photocurrent by more than 100× compared to conventional top‐contact configurations. They have also improved efficiency and open‐circuit voltage. These developments open the door to wearable transparent electronics, quantum optoelectronic devices, next‐generation solar cells surpassing traditional efficiency limits, and self‐powered photodetectors. However, significant challenges remain, including the need for interdisciplinary collaboration, scalable device integration, and material optimization to lower carrier recombination. To fully realize the transformative impact of BPVE‐enabled 2D systems across the energy, sensing, and quantum technology sectors, these obstacles must be overcome.^[^ ^149^ ^]^

### 13.7 BPVE Beyond 1T and 3R Phases in 2D Materials

Although the 1T and 3R phases of 2D TMDs have received most of the attention because of their inherent inversion symmetry breaking and advantageous stacking geometries, other phases, including 2Hd (distorted hexagonal), 1T′′ or 1T′′′ (distorted octahedral), and Td (orthorhombic), also show tunable symmetry and electronic properties that could support BPVE under the right circumstances, such as strain, alloying, or stacking modifications. Materials with improved BPVE response can be designed using these structurally and electronically diverse phases, especially metastable ones. Phase engineering to break symmetry in nominally centrosymmetric systems, interface and heterostructure design to take advantage of layer‐dependent inversion asymmetry, and first‐principles modeling to find new candidates with strong shift currents are all critical to the advancement of this field. Experimental investigations into the effects of strain, doping, and electric fields are also necessary. The development of scalable, junction‐free solar energy technologies will be accelerated, and a deeper understanding of quantum photovoltaic mechanisms in low‐dimensional materials will be gained by extending BPVE exploration beyond the 1T and 3R phases.

### 13.8 Accelerating BPVE Discovery in 2D Materials through Machine Learning

BPVE in low‐dimensional materials can be precisely identified and optimized with machine learning (ML). Machine learning algorithms can quickly screen a large number of 2D materials to identify those with non‐centrosymmetric structures and strong shift current responses—two important indicators of BPVE—by processing large datasets of structural, electronic, and symmetry‐related properties. Traditional approaches frequently overlook intricate structure–property correlations, which are revealed by models trained on first‐principles or experimental data. Additionally, ML facilitates inverse design by suggesting heterostructures, strain states, or atomic configurations that are likely to improve BPVE. ML speeds up discovery and focuses experimental efforts on the most promising candidates when combined with high‐throughput density functional theory and symmetry analysis. This opens the door for effective, junction‐free optoelectronic and solar energy applications.

### 13.9 Asymmetric Photoresponse

By strategically utilizing the asymmetric photoresponse for next‐generation polarization‐sensitive detectors with improved performance, scalability, and multifunctionality for advanced imaging, communication, and energy‐harvesting applications can be designed and manufactured.^[^ ^150^, ^151^, ^152^ ^]^ Strong polarization sensitivity is also provided by this inherent asymmetry, enabling devices to discriminate between polarization states for use in quantum, communication, and imaging applications. A scalable path to next‐generation polarization‐sensitive optoelectronics and energy‐harvesting systems is provided by the strategic combination of intrinsic and engineered asymmetry. The PV effect enables novel devices with high‐speed, self‐powered operation, paving the way for future applications in artificial synapses and neuromorphic computing.^[^ ^153^, ^154^, ^155^ ^]^

### 13.10 2D TMD PVs, vdWs Heterostructures, and BPVE

Next‐generation photovoltaics are showing promise thanks to 2D TMDs, which offer lightweight, ultrathin structures with tunable bandgaps and strong optical absorption.^[^ ^156^ ^]^ Simulations and detailed balance models indicate significant room for improvement, with projections of ≈12.9% in superlattice designs, ≈25% in optimized single‐junction devices, and up to 28–29% in tandem TMD–silicon architectures.^[^ ^156^, ^157^ ^]^ Current laboratory efficiencies for single‐junction TMD solar cells are modest at ≈5–7% (e.g., 5.1% for flexible WSe_2_ and up to 6.88% for MoS_2_ nanocube heterostructures).^[^ ^158^ ^]^ Key barriers include Fermi‐level pinning at contacts, limited doping strategies, interfacial contamination during flexible fabrication, and challenges in large‐area defect‐free synthesis, passivation, and stability. 2D TMDs vdWs heterostructures can also take advantage of BPVE, which occurs in non‐centrosymmetric TMD phases and produces a constant photocurrent under uniform illumination without the use of p–n junctions, in addition to traditional PV mechanisms. High photovoltages that surpass the material bandgap may result from BPVE's utilization of the inherent asymmetry of the crystal lattice, which permits shift currents impacted by electronic band structure and Berry phase effects. 2D TMD PVs have distinct advantages in ultralight weight, flexibility, and high specific power (up to 46 W g^−1^), making them perfect for wearables, aerospace, and other mobile or niche applications where conventional rigid modules are impractical.

## 14 Conclusion

### 14.1 Summary of Key Findings

- In 2D TMDs, phase engineering provides a powerful means to break inversion symmetry and activate BPVE, especially through transitions such as 2H → 1T/1T′ and 2H → 3R. Through shift current mechanisms, these transitions also considerably change the electronic band structure, allowing for increased photocurrent generation.
- To improve light‐matter interactions, structural alterations such as twisting, rolling, straining, and distorting 2D layers produce Moiré superlattices, modulate the local electric field, and introduce spatial asymmetry. These characteristics are essential for increasing BPVE's magnitude and directionality.
- In otherwise centrosymmetric 2D systems, vdWs heterostructures offer a flexible method of inducing and customizing BPVE through interfacial symmetry breaking and tunable interlayer coupling.
- Polarization engineering improves carrier separation and photocurrent, by introducing intrinsic and extrinsic electric fields by utilizing piezoelectric, ferroelectric, and dual‐polarization effects. The internal electric landscape is further modulated by depolarization fields and edge contact configurations, which intensifies BPVE responses.
- The combination of phase, structure, and polarization provides a multifaceted toolkit for creating 2D optoelectronic materials of the future. These methods, when carefully integrated, provide previously unheard‐of control over the symmetry, band topology, and photoresponse of 2D TMDs.
- Collectively, these engineering techniques point to the development of non‐centrosymmetric, high‐efficiency 2D materials with adjustable BPVE, paving the way for novel solar energy harvesting and quantum optoelectronic devices.

### 14.2 Final Comments on Design Principles and Research Directions

- A key design principle for the realization and improvement of BPVE in 2D TMDs is the intentional manipulation of phase, structure, and polarization to break inversion symmetry. This control of symmetry must be balanced with material stability and electronic tunability in order for engineering strategies to be successful.
- Phase transitions (e.g., 2H → 1T′, 2H → 3R) can be controlled through scalable techniques like strain application, electrostatic gating, or substrate‐induced effects.
- BPVE can be improved by precisely adjusting the structure to modulate local symmetry and Moiré potentials through twist angle control, curvature engineering, and strain gradients.
- In vdWs heterostructures, rational stacking could be utilized to maximize interlayer charge transfer dynamics and produce interfacial asymmetries.
- Intrinsic polarization, particularly in piezoelectric and ferroelectric 2D materials, could be utilized to generate directional and switchable PV responses.
- Computational predictive models could be developed that relate BPVE strength to symmetry breaking on various 2D platforms.
- Hybrid materials can be created with synergistic effects by combining several symmetry‐breaking mechanisms (for example: phase + ferroelectricity + strain).
- Nanoscale characterization techniques, such as ultrafast spectroscopy and photoconductive AFM, could be developed to identify and measure BPVE contributions.
- BPVE‐active 2D TMDs could be combined with useful components, such as solar cells, light‐controlled logic elements, and self‐powered photodetectors.
- The future of BPVE‐enabled quantum and energy technologies will ultimately be determined by the convergence of materials design, nanoscale control, and device integration.

## Conflict of Interest

The authors declare no conflict of interest.

## References

[1] T. Hong , T. Chen , D. Jin , Y. Zhu , H. Gao , K. Zhao , T. Zhang , W. Ren , G. Cao , npj Quantum Mater. 2025, 10, 12.

[2] S. Huo , H. Qu , F. Meng , Z. Zhang , Z. Yang , S. Zhang , X. Hu , E. Wu , Nano Lett. 2024, 24, 11937. 39269273 10.1021/acs.nanolett.4c03263

[3] S. Aftab , Z. Ali , M. I. Hussain , M. A. Assiri , N. Rubab , F. Ozel , E. Akman , Carbon Energy 2025, 7, 70018.

[4] A. O. Ali , A. T. Elgohr , M. H. El‐Mahdy , H. M. Zohir , A. Z. Emam , M. G. Mostafa , M. Al‐Razgan , H. M. Kasem , M. S. Elhadidy , Energy Convers. Manag.: X 2025, 26, 100952.

[5] L. Duan , D. Walter , N. Chang , J. Bullock , D. Kang , S. P. Phang , K. Weber , T. White , D. Macdonald , K. Catchpole , H. Shen , Nat. Rev. Mater. 2023, 8, 261.

[6] M. J. Talukder , M. Akteruzzaman , Z. Hasan , E. Haque , Am. J. Adv. Technol. Eng. Sol. 2025, 1, 201.

[7] A. A. Said , X. Li , E. Ugur , F. H. Isikgor , J. Liu , R. Azmi , M. De Bastiani , E. Aydin , S. Zhang , A. S. Subbiah , T. G. Allen , G. Lubineau , I. Mcculloch , S. De Wolf , Energy Fuels 2025, 39, 10134.

[8] S. Aftab , B. S. Goud , Z. Ali , M. A. Assiri , J. H. Kim , N. Rubab , E. Akman , Chem. Eng. J. 2025, 516, 164224.

[9] H. Zhou , D. Li , Q. Lv , C. Lee , Chem. Soc. Rev. 2025, 54, 5342. 40354162 10.1039/d4cs00427b

[10] J. Wei , C. Xu , B. Dong , C.‐W. Qiu , C. Lee , Nat. Photonics 2021, 15, 614.

[11] G. Cheng , C. Y. Kuan , K. W. Lou , Y. P. Ho , Adv. Mater. 2025, 37, 2313935. 38379512 10.1002/adma.202313935 PMC11733724

[12] Y. Dang , X. Tao , Matter 2022, 5, 2659.

[13] Z. Dai , A. M. Rappe , Chem. Phys. Rev. 2023, 4, 011303.

[14] C. Xiao‐Juan , X. Kang , Z. Xiu , L. Hai‐Yun , X. Qi‐Hua , Acta Phys. Sin. 2023, 72, 237201.

[15] C. Wang , X. Cheng , K. H. Luo , K. Nandakumar , Z. Wang , M. Ni , X. Bi , J. Zhang , C. Wang , Mater. Sci. Eng.: R: Rep. 2025, 165, 101010.

[16] R. P. Tiwari , B. Birajdar , R. K. Ghosh , Phys. Rev. B 2020, 101, 235448.

[17] A. M. Cook , B. M. Fregoso , F. De Juan , S. Coh , J. E. Moore , Nat. Commun. 2017, 8, 14176. 28120823 10.1038/ncomms14176 PMC5288499

[18] M. Ezawa , Phys. Rev. B 2025, 111, L201405.

[19] S. Aftab , M. Z. Iqbal , Z. Haider , M. W. Iqbal , G. Nazir , M. A. Shehzad , Adv. Opt. Mater. 2022, 10, 2201288.

[20] B. Ehrler , E. Alarcón‐Lladó , S. W. Tabernig , T. Veeken , E. C. Garnett , A. Polman , ACS Energy Lett. 2020, 9, 3029.

[21] T. S Markvart , Wiley Interdiscip. Rev.: Energy Environ. 2022, 11, 430.

[22] Y. H. Lee , Am. Assoc. Adv. Sci. 2024, 383, ado4308.

[23] K. Wang , L. Zheng , Y. Hou , A. Nozariasbmarz , B. Poudel , J. Yoon , T. Ye , D. Yang , A. V. Pogrebnyakov , V. Gopalan , S. Priya , Joule 2022, 6, 756.

[24] S. A. Mann , R. R. Grote , R. M. Osgood , A. Alù , E. C. Garnett , ACS Nano 2016, 10, 8620. 27580421 10.1021/acsnano.6b03950

[25] M. Stolterfoht , C. M. Wolff , Y. Amir , A. Paulke , L. Perdigón‐Toro , P. Caprioglio , D. Neher , Energy Environ. Sci. 2017, 10, 1530.

[26] M. A. Green , A. W. Ho‐Baillie , ACS Energy Lett. 2019, 4, 1639.

[27] A. M. Oni , A. S. M. Mohsin , Md. M Rahman , M. B. Hossain Bhuian , Energy Rep. 2024, 11, 3345.

[28] E. K. Solak , E. Irmak , RSC Adv. 2023, 13, 12244. 37091609 10.1039/d3ra01454a PMC10114284

[29] K. Devendra , R. Wagle , R. Gaib , A. Shrivastava , L. Mishra , Am. J. Eng. Res 2020, 9, 218.

[30] A. T. Raisa , S. N. Sakib , M. J. Hossain , K. A. Rocky , A. Kowsar , Solar Energy Adv. 2025, 5, 100105.

[31] A. Mukherjee , D. Ren , P.‐E. Vullum , J. Huh , B.‐O. Fimland , H. Weman , ACS Photonics 2021, 8, 2355.

[32] P. Chauhan , S. Agarwal , V. Srivastava , S. Maurya , M. K. Hossain , J. Madan , R. K. Yadav , P. Lohia , D. K. Dwivedi , A. A. Alothman , Prog. Photovoltaics 2024, 32, 156.

[33] W. S. Yang , J. H. Noh , N. J. Jeon , Y. C. Kim , S. Ryu , J. Seo , S. I. Seok , Science 2015, 348, 1234. 25999372 10.1126/science.aaa9272

[34] A. Kojima , K. Teshima , Y. Shirai , T. Miyasaka , J. Am. Chem. Soc. 2009, 131, 6050. 19366264 10.1021/ja809598r

[35] A. Kowsar , S. C. Debnath , M. Shafayet‐Ul‐Islam , M. J. Hossain , M. Hossain , A. Chowdhury , G. Hashmi , S. F. U. Farhad , *arXiv preprint arXiv:2405.01550*, 2024.

[36] I. Grinberg , D. V West , M. Torres , G. Gou , D. M. Stein , L. Wu , G. Chen , E. M. Gallo , A. R. Akbashev , P. K. Davies , J. E. Spanier , A. M. Rappe , Nature 2013, 503, 509. 24213630 10.1038/nature12622

[37] S. M. Young , A. M. Rappe , Phys. Rev. Lett. 2012, 109, 116601. 23005660 10.1103/PhysRevLett.109.116601

[38] Y. Zhou , X. Zhou , X. L. Yu , Z. Liang , X. Zhao , T. Wang , J. Miao , X. Chen , Nat. Commun. 2024, 15, 501. 38218730 10.1038/s41467-024-44792-4 PMC10787835

[39] L. Z. Tan , F. Zheng , S. M. Young , F. Wang , S. Liu , A. M. Rappe , npj Comput. Mater. 2016, 2, 16026.

[40] Y. M. Xie , N. Nagaosa , Proc. Natl. Acad. Sci. USA 2025, 122, 2424294122. 10.1073/pnas.2424294122 PMC11892632 40014566

[41] A. Pusch , U. Römer , D. Culcer , N. J. Ekins‐Daukes , PRX Energy 2023, 2, 013006.

[42] Y. Li , J. Fu , X. Mao , C. Chen , H. Liu , M. Gong , H. Zeng , Nat. Commun. 2021, 12, 5896. 34625541 10.1038/s41467-021-26200-3 PMC8501070

[43] S. Aftab , M. A. Shehzad , H. M. Salman Ajmal , F. Kabir , M. Z. Iqbal , A. A. Al‐Kahtani , ACS Nano 2023, 17, 17884. 37656985 10.1021/acsnano.3c03593

[44] Z. Qian , J. Zhou , H. Wang , S. Liu , npj Comput. Mater. 2023, 9, 67.

[45] E. Wu , Y. Ma , Q. Tian , Z. Wang , Z. Song , S. Huo , F. Meng , Y. Xie , C. Pan , ACS Nano 2025, 19, 35701. 41039875 10.1021/acsnano.5c11809

[46] E. Camarillo Abad , H. J. Joyce , L. C. Hirst , ACS Photonics 2022, 9, 2724. 35996371 10.1021/acsphotonics.2c00472 PMC9389614

[47] A. Ravilla , C. A. Perini , J.‐P. Correa‐Baena , A. W. Ho‐Baillie , I. Celik , Energy Adv. 2024, 3, 800.

[48] S. Qiao , J. Liu , C. Yao , N. Yang , F. Zheng , W. Meng , Y. Wan , P. C. Y. Chow , D.‐K. Ki , L. Zhang , Y. Shi , L.‐J. Li , Light: Sci. Appl. 2025, 14, 22. 39743635 10.1038/s41377-024-01691-z PMC11693758

[49] S. Aftab , S. Hussain , A. A. Al‐Kahtani , Adv. Mater. 2023, 35, 2301280. 10.1002/adma.202301280 37104492

[50] C. A. Nelson , N. R. Monahan , X.‐Y. Zhu , Energy Environ. Sci. 2013, 6, 3508.

[51] Z. Zhou , J. Lv , C. Tan , L. Yang , Z. Wang , Adv. Funct. Mater. 2024, 34, 2316175.

[52] S. Aftab , H. H. Hegazy , Small 2023, 19, 2205778. 10.1002/smll.202205778 36732842

[53] M. Nakamura , S. Horiuchi , F. Kagawa , N. Ogawa , T. Kurumaji , Y. Tokura , M. Kawasaki , Nat. Commun. 2017, 8, 281. 28819286 10.1038/s41467-017-00250-y PMC5561111

[54] H. Yuan , X. Wang , B. Lian , H. Zhang , X. Fang , B. Shen , G. Xu , Y. Xu , S.‐C. Zhang , H. Y. Hwang , Y. Cui , Nat. Nanotechnol. 2014, 9, 851. 25194947 10.1038/nnano.2014.183

[55] A.‐Y. Lu , H. Zhu , J. Xiao , C.‐P. Chuu , Y. Han , M.‐H. Chiu , C.‐C. Cheng , C.‐W. Yang , K.‐H. Wei , Y. Yang , Y. Wang , D. Sokaras , D. Nordlund , P. Yang , D. A. Muller , M.‐Y. Chou , X. Zhang , L.‐J. Li , Nat. Nanotechnol. 2017, 12, 744. 28507333 10.1038/nnano.2017.100

[56] Q. Ma , S.‐Y. Xu , H. Shen , D. Macneill , V. Fatemi , T.‐R. Chang , A. M. Mier Valdivia , S. Wu , Z. Du , C.‐H. Hsu , S. Fang , Q. D. Gibson , K. Watanabe , T. Taniguchi , R. J. Cava , E. Kaxiras , H.‐Z. Lu , H. Lin , L. Fu , N. Gedik , P. Jarillo‐Herrero , Nature 2019, 565, 337. 30559379 10.1038/s41586-018-0807-6

[57] W. Choi , N. Choudhary , G. H. Han , J. Park , D. Akinwande , Y. H. Lee , Mater. Today 2017, 20, 116.

[58] S. Wi , H. Kim , M. Chen , H. Nam , L. J. Guo , E. Meyhofer , X. Liang , ACS Nano 2014, 8, 5270. 24783942 10.1021/nn5013429

[59] F. Liu , L. You , K. L. Seyler , X. Li , P. Yu , J. Lin , X. Wang , J. Zhou , H. Wang , H. He , S. T. Pantelides , W. Zhou , P. Sharma , X. Xu , P. M. Ajayan , J. Wang , Z. Liu , Nat. Commun. 2016, 7, 12357. 27510418 10.1038/ncomms12357 PMC4987531

[60] S. Qiao , J. Liu , C. Yao , N. Yang , F. Zheng , W. Meng , Y. Wan , P. C. Y. Chow , D.‐K. Ki , L. Zhang , Y. Shi , L.‐J. Li , Light Sci. Appl. 2025, 14, 22. 39743635 10.1038/s41377-024-01691-z PMC11693758

[61] R.‐C. Xiao , Y. Gao , H. Jiang , W. Gan , C. Zhang , H. Li , npj Comput. Mater. 2022, 8, 138.

[62] S. Manzeli , D. Ovchinnikov , D. Pasquier , O. V. Yazyev , A. Kis , Nat. Rev. Mater. 2017, 2, 17033.

[63] Y.‐X. Zhao , H. Jin , Z.‐Y. Han , X. Zhao , Y.‐N. Ren , R.‐H. Zhang , X.‐F. Zhou , W. Duan , B. Huang , Y. Zhang , L. He , Nat. Commun. 2025, 16, 3659. 40246906 10.1038/s41467-025-59007-7 PMC12006514

[64] M. A. Akhound , K. W. Jacobsen , K. S. Thygesen , J. Am. Chem. Soc. 2025, 147, 5743. 39919305 10.1021/jacs.4c13863 PMC11848928

[65] S. M. Nahid , S. Nam , A. M. van der Zande , ACS Nano 2024, 18, 14198. 38771928 10.1021/acsnano.3c11558

[66] Z. Zeng , Z. Tian , Y. Wang , C. Ge , F. Strauß , K. Braun , P. Michel , L. Huang , G. Liu , D. Li , M. Scheele , M. Chen , A. Pan , X. Wang , Nat. Commun. 2024, 15, 5355. 38918419 10.1038/s41467-024-49760-6 PMC11199638

[67] X. Huang , Q. Wang , K. Song , Q. Hu , H. Zhang , X. Gao , M. Long , J. Xu , Z. Chen , G. Zhou , B. Wu , Nano Lett. 2025, 25, 1495. 39810623 10.1021/acs.nanolett.4c05418

[68] Y. Gong , R. Duan , Y. Hu , Y. Wu , S. Zhu , X. Wang , Q. Wang , S. P. Lau , Z. Liu , B. K. Tay , Nat. Commun. 2025, 16, 230. 39747133 10.1038/s41467-024-55562-7 PMC11695928

[69] W. Wang , Y. Xiao , T. Li , X. Lu , N. Xu , Y. Cao , J. Phys. Chem. Lett. 2024, 15, 3549. 38526184 10.1021/acs.jpclett.4c00470

[70] T. Akamatsu , T. Ideue , L. Zhou , Y. Dong , S. Kitamura , M. Yoshii , D. Yang , M. Onga , Y. Nakagawa , K. Watanabe , T. Taniguchi , J. Laurienzo , J. Huang , Z. Ye , T. Morimoto , H. Yuan , Y. Iwasa , Science 2021, 372, 68. 33795452 10.1126/science.aaz9146

[71] S. Aftab , M. Z. Iqbal , M. W. Iqbal , M. A. Shehzad , Laser Photonics Rev. 2023, 17, 2200429.

[72] Y. Dong , M.‐M. Yang , M. Yoshii , S. Matsuoka , S. Kitamura , T. Hasegawa , N. Ogawa , T. Morimoto , T. Ideue , Y. Iwasa , Nat. Nanotechnol. 2023, 18, 36. 36411374 10.1038/s41565-022-01252-8

[73] Y. J. Zhang , T. Ideue , M. Onga , F. Qin , R. Suzuki , A. Zak , R. Tenne , J. H. Smet , Y. Iwasa , Nature 2019, 570, 349. 31217597 10.1038/s41586-019-1303-3

[74] S. Chen , Z. Liang , J. Miao , X.‐L. Yu , S. Wang , Y. Zhang , H. Wang , Y. Wang , C. Cheng , G. Long , T. Wang , L. Wang , H. Zhang , X. Chen , Nat. Commun. 2024, 15, 8834. 39397018 10.1038/s41467-024-53125-4 PMC11471851

[75] B. A. Hanedar , M. C. Onbaşlı , Phys. Chem. Chem. Phys. 2025, 27, 1809. 39692347 10.1039/d4cp04017a PMC11698123

[76] C. R. Ryder , J. D. Wood , S. A. Wells , M. C. Hersam , ACS Nano 2016, 10, 3900. 27018800 10.1021/acsnano.6b01091

[77] F. Wang , C. Wang , A. Chaves , C. Song , G. Zhang , S. Huang , Y. Lei , Q. Xing , L. Mu , Y. Xie , H. Yan , Nat. Commun. 2021, 12, 5628. 34561443 10.1038/s41467-021-25941-5 PMC8463555

[78] A. Krasnok , S. Lepeshov , A. Alú , Opt. Express 2018, 26, 15972. 30114850 10.1364/OE.26.015972

[79] J. Zhou , T.‐Y. Cai , S. Ju , Phys. Rev. Res. 2020, 2, 033288.

[80] A. Sreevalsan , H. Choi , Light: Sci. Appl. 2025, 14, 89. 39979246 10.1038/s41377-025-01764-7 PMC11842759

[81] N. Urakami , S. Ozaki , Y. Hashimoto , Appl. Phys. Lett. 2024, 125, 073102.

[82] M. S. Sokolikova , C. Mattevi , Chem. Soc. Rev. 2020, 49, 3952. 32452481 10.1039/d0cs00143k

[83] A. V. Kolobov , J. Tominaga , in Two‐Dimensional Transition‐Metal Dichalcogenides, Springer, New York City, New York 2016.

[84] H. Chen , J. Zhang , D. Kan , J. He , M. Song , J. Pang , S. Wei , K. Chen , Crystals 2022, 12, 1381.

[85] D. Sahoo , S. Senapati , R. Naik , FlatChem 2022, 36, 100455.

[86] W. Zhao , F. Ding , Nanoscale 2017, 9, 2301. 28128387 10.1039/c6nr08628d

[87] M. A. Macchione , R. Mendoza‐Cruz , L. Bazán‐Diaz , J. J. Velázquez‐Salazar , U. Santiago , M. J. Arellano‐Jiménez , J. F. Perez , M. José‐Yacamán , J. E. Samaniego‐Benitez , New J. Chem. 2020, 44, 1190.

[88] S. S. Awate , K. Xu , J. Liang , B. Katz , R. Muzzio , V. H. Crespi , J. Katoch , S. K. Fullerton‐Shirey , ACS Nano 2023, 17, 22388. 37947443 10.1021/acsnano.3c04701 PMC10690768

[89] K.‐A. N. Duerloo , Y. Li , E. J. Reed , Nat. Commun. 2014, 5, 4214. 24981779 10.1038/ncomms5214

[90] S. Song , D. H. Keum , S. Cho , D. Perello , Y. Kim , Y. H. Lee , Nano Lett. 2016, 16, 188. 26713902 10.1021/acs.nanolett.5b03481

[91] D. H. Keum , S. Cho , J. H. Kim , D.‐H. Choe , H.‐J. Sung , M. Kan , H. Kang , J.‐Y. Hwang , S. W. Kim , H. Yang , K. J. Chang , Y. H. Lee , Nat. Phys. 2015, 11, 482.

[92] H. Hughes , R. Friend , J. Phys. C: Solid State Phys. 1978, 11, L103.

[93] J. Shi , P. Yu , F. Liu , P. He , R. Wang , L. Qin , J. Zhou , X. Li , J. Zhou , X. Sui , S. Zhang , Y. Zhang , Q. Zhang , T. C. Sum , X. Qiu , Z. Liu , X. Liu , Adv. Mater. 2017, 29, 1701486. 10.1002/adma.201701486 28590583

[94] S. Fan , C. Han , K. He , L. Bai , L. Q. Chen , H. Shi , C. Shen , T. Yang , Adv. Mater. 2025, 37, 2418839. 40345971 10.1002/adma.202418839 PMC12288825

[95] S. Cho , S. Kim , J. H. Kim , J. Zhao , J. Seok , D. H. Keum , J. Baik , D.‐H. Choe , K. J. Chang , K. Suenaga , S. W. Kim , Y. H. Lee , H. Yang , Science 2015, 349, 625. 26250680 10.1126/science.aab3175

[96] S. Yuan , X. Luo , H. L. Chan , C. Xiao , Y. Dai , M. Xie , J. Hao , Nat. Commun. 2019, 10, 1775. 30992431 10.1038/s41467-019-09669-x PMC6467908

[97] L. Yadgarov , B. Višić , T. Abir , R. Tenne , A. Y. Polyakov , R. Levi , T. V. Dolgova , V. V. Zubyuk , A. A. Fedyanin , E. A. Goodilin , T. Ellenbogen , R. Tenne , D. Oron , Phys. Chem. Chem. Phys. 2018, 20, 20812. 30004095 10.1039/c8cp02245c

[98] H. Ai , Y. Kong , D. Liu , F. Li , J. Geng , S. Wang , K. H. Lo , H. Pan , J. Phys. Chem. C 2020, 124, 11221.

[99] P. Johari , V. B. Shenoy , ACS Nano 2012, 6, 5449. 22591011 10.1021/nn301320r

[100] S. Pace , L. Martini , D. Convertino , D. H. Keum , S. Forti , S. Pezzini , F. Fabbri , V. Mišeikis , C. Coletti , ACS Nano 2021, 15, 4213. 33605730 10.1021/acsnano.0c05936 PMC8023802

[101] R. Beams , L. G. Cançado , S. Krylyuk , I. Kalish , B. Kalanyan , A. K. Singh , K. Choudhary , A. Bruma , P. M. Vora , F. Tavazza , A. V. Davydov , S. J. Stranick , ACS Nano 2016, 10, 9626. 27704774 10.1021/acsnano.6b05127 PMC5542881

[102] S.‐Y. Chen , T. Goldstein , D. Venkataraman , A. Ramasubramaniam , J. Yan , Nano Lett. 2016, 16, 5852. 27517466 10.1021/acs.nanolett.6b02666

[103] M.‐M. Yang , D. J. Kim , M. Alexe , Science 2018, 360, 904. 29674433 10.1126/science.aan3256

[104] S. Nadupalli , J. Kreisel , T. Granzow , Sci. Adv. 2019, 5, aau9199. 10.1126/sciadv.aau9199 PMC6397022 30838328

[105] X. Cui , Z. Kong , E. Gao , D. Huang , Y. Hao , H. Shen , C.‐a. Di , Z. Xu , J. Zheng , D. Zhu , Nat. Commun. 2018, 9, 1301. 29615627 10.1038/s41467-018-03752-5 PMC5883047

[106] S. Aftab , M. Z. Iqbal , Y. S. Rim , Small 2023, 19, 2205418. 10.1002/smll.202205418 36373722

[107] J. M. de Albornoz‐Caratozzolo , F. Cervantes‐Sodi , Nanoscale Adv. 2024, 6, 79. 10.1039/d3na00301a PMC10729892 38125603

[108] R. A. Wells , H. Johnson , C. R. Lhermitte , S. Kinge , K. Sivula , ACS Appl. Nano Mater. 2019, 2, 7705.

[109] W. Shockley , Bell Syst. Tech. J. 1949, 28, 435.

[110] H. Zeng , G.‐B. Liu , J. Dai , Y. Yan , B. Zhu , R. He , L. Xie , S. Xu , X. Chen , W. Yao , X. Cui , Sci. Rep. 2013, 3, 1608. 23575911 10.1038/srep01608 PMC3622914

[111] M. Freitag , T. Low , F. Xia , P. Avouris , Nat. Photonics 2013, 7, 53.

[112] Z. Peng , X. Chen , Y. Fan , D. J. Srolovitz , D. Lei , Light: Sci. Appl. 2020, 9, 190. 33298826 10.1038/s41377-020-00421-5 PMC7680797

[113] L. I. Bendavid , Y. Zhong , Z. Che , Y. Konuk , J. Appl. Phys. 2022, 132, 225303.

[114] A. Jangir , D. T. Ho , U. Schwingenschlögl , J. Phys. Chem. Lett. 2025, 16, 811. 39812594 10.1021/acs.jpclett.4c03464 PMC11770754

[115] J. Wang , L. He , Y. Zhang , H. Nong , S. Li , Q. Wu , J. Tan , B. Liu , Adv. Mater. 2024, 36, 2314145. 10.1002/adma.202314145 38339886

[116] J. Jiang , Z. Chen , Y. Hu , Y. Xiang , L. Zhang , Y. Wang , G.‐C. Wang , J. Shi , Nat. Nanotechnol. 2021, 16, 894. 34140672 10.1038/s41565-021-00919-y

[117] Z. Liang , X. Zhou , L. Zhang , X.‐L. Yu , Y. Lv , X. Song , Y. Zhou , H. Wang , S. Wang , T. Wang , P. P. Shum , Q. He , Y. Liu , C. Zhu , L. Wang , X. Chen , Nat. Commun. 2023, 14, 4230. 37454221 10.1038/s41467-023-39995-0 PMC10349808

[118] X. Mu , Q. Xue , Y. Sun , J. Zhou , Phys. Rev. Res. 2023, 5, 013001.

[119] Q. Ma , S.‐Y. Xu , C.‐K. Chan , C.‐L. Zhang , G. Chang , Y. Lin , W. Xie , T. Palacios , H. Lin , S. Jia , P. A. Lee , P. Jarillo‐Herrero , N. Gedik , Nat. Phys. 2017, 13, 842.

[120] Y. Zhang , T. Ideue , M. Onga , F. Qin , R. Suzuki , A. Zak , R. Tenne , J. Smet , Y. Iwasa , Nature 2019, 570, 349. 31217597 10.1038/s41586-019-1303-3

[121] X. Luo , Q. Li , Y. Wang , Materials 2024, 17, 844.38399095

[122] Y. Liu , J. Guo , E. Zhu , L. Liao , S.‐J. Lee , M. Ding , I. Shakir , V. Gambin , Y. Huang , X. Duan , Nature 2018, 557, 696. 29769729 10.1038/s41586-018-0129-8

[123] J. Toušek , Phys. Status Solidi 1991, 128, 531.

[124] X. Wang , X. Zhou , A. Cui , M. Deng , X. Xu , L. Xu , Y. Ye , K. Jiang , L. Shang , L. Zhu , J. Zhang , Y. Li , Z. Hu , J. Chu , Mater. Horiz. 2021, 8, 1985. 34846475 10.1039/d1mh00024a

[125] R.‐C. Xiao , Y. Gao , H. Jiang , W. Gan , C. Zhang , H. Li , *arXiv* 2022, 2201.04980.

[126] K. Kyuma , E. Lange , J. Ohta , A. Hermanns , B. Banish , M. Oita , Nature 1994, 372, 197.

[127] J. Jiang , X. a. Feng , F. Liu , Y. Xu , H. Huang , IEEE Access 2019, 7, 20607.

[128] X. Chen , K. Xu , T. Qin , Y. Wang , Q. Xiong , H. Liu , Nanoscale 2025, 17, 5005. 39885812 10.1039/d4nr05317f

[129] J. Rijnsdorp , F. Jellinek , J. Less Common Met. 1978, 61, 79.

[130] C. Liu , X. Zhang , X. Wang , Z. Wang , I. Abdelwahab , I. Verzhbitskiy , Y. Shao , G. Eda , W. Sun , L. Shen , K. P. Loh , ACS Nano 2023, 17, 7170. 37036127 10.1021/acsnano.2c09267

[131] D. Qiu , P. Hou , J. Wang , X. Ouyang , Appl. Phys. Lett. 2023, 123, 111102.

[132] V. M. Fridkin , Crystallogr. Rep. 2001, 46, 654.

[133] J. Yu , B. Huang , S. Yang , Y. Zhang , Y. Bai , C. Song , W. Ming , W. Liu , J. Wang , C. Li , Q. Wang , J. Li , Nano Lett. 2024, 24, 6337. 38742772 10.1021/acs.nanolett.4c01173

[134] M. Buscema , M. Barkelid , V. Zwiller , H. S. van der Zant , G. A. Steele , A. Castellanos‐Gomez , Nano Lett. 2013, 13, 358. 23301811 10.1021/nl303321g

[135] L. You , F. Zheng , L. Fang , Y. Zhou , L. Z. Tan , Z. Zhang , G. Ma , D. Schmidt , A. Rusydi , L. Wang , L. Chang , A. M. Rappe , J. Wang , Sci. Adv. 2018, 4, aat3438. 10.1126/sciadv.aat3438 PMC6035034 29984307

[136] Y. Bai , W. Hao , Y. Wang , J. Tian , C. Wang , Y. Lei , Y. Yang , X. Yao , Q. Liu , C. Li , M. Gu , J. Wang , PRX Energy 2024, 3, 023004.

[137] W. Ji , K. Yao , Y. C. Liang , Phys. Rev. B 2011, 84, 094115.

[138] Y. T. Huang , N. K. Chen , Z. Z. Li , X. P. Wang , H. B. Sun , S. Zhang , X. B. Li , InfoMat 2022, 4, 12341.

[139] C. Cui , W.‐J. Hu , X. Yan , C. Addiego , W. Gao , Y. Wang , Z. Wang , L. Li , Y. Cheng , P. Li , X. Zhang , H. N. Alshareef , T. Wu , W. Zhu , X. Pan , L.‐J. Li , Nano Lett. 2018, 18, 1253. 29378142 10.1021/acs.nanolett.7b04852

[140] W. Ding , J. Zhu , Z. Wang , Y. Gao , D. Xiao , Y. Gu , Z. Zhang , W. Zhu , Nat. Commun. 2017, 8, 14956. 28387225 10.1038/ncomms14956 PMC5385629

[141] W. Shockley , H. Queisser , in Renewable energy, Routledge, England, United Kingdom 2018.

[142] D. Rauh , A. Wagenpfahl , C. Deibel , V. Dyakonov , Appl. Phys. Lett. 2011, 98, 133301.

[143] S. Qiao , J. Liu , C. Yao , N. Yang , F. Zheng , W. Meng , Y. Wan , P. C. Y. Chow , D.‐K. Ki , L. Zhang , Y. Shi , L.‐J. Li , Light: Sci. Appl. 2025, 14, 22. 39743635 10.1038/s41377-024-01691-z PMC11693758

[144] Z. Yang , C. Kim , K. Y. Lee , M. Lee , S. Appalakondaiah , C.‐H. Ra , K. Watanabe , T. Taniguchi , K. Cho , E. Hwang , J. Hone , W. J. Yoo , Adv. Mater. 2019, 31, 1808231. 10.1002/adma.201808231 31066475

[145] A. Bablich , S. Kataria , M. C. Lemme , Electronics 2016, 5, 13.

[146] Z. Lin , Y. Liu , U. Halim , M. Ding , Y. Liu , Y. Wang , C. Jia , P. Chen , X. Duan , C. Wang , F. Song , M. Li , C. Wan , Y. Huang , X. Duan , Nature 2018, 562, 254. 30283139 10.1038/s41586-018-0574-4

[147] X. Chen , S. Lv , Y. Liu , H. Gu , X. Sun , Q. Hu , Y. Zhao , Z. Li , T. Guo , J. Kang , Angew. Chem., Int. Ed. 2025, 202515222. 10.1002/anie.202515222 40916753

[148] Y. Guan , Y. Ding , Y. Fang , J. Li , Y. Liu , R. Wang , J. Hao , H. Xie , C. Xu , L. Zhen , Y. Li , L. Yang , Nat. Commun. 2025, 16, 4149. 40320417 10.1038/s41467-025-59520-9 PMC12050275

[149] C. Wang , J. Hao , L. Duan , D. Zhao , Y. Ma , J. Zhang , P. Ma , D. Han , F. Meng , Ceram. Int. 2025, 51, 35133.

[150] K. Xin , Z. Zhou , S. Qiu , T. Liu , Y. Yu , J. Yang , W. Hu , Z. Wei , Nat. Rev. Electr. Eng. 2025, 2, 480.

[151] S. Wang , Z. Yang , D. Wang , C. Tan , L. Yang , Z. Wang , ACS Appl. Mater. Interfaces 2023, 15, 3357. 36599121 10.1021/acsami.2c19660

[152] X. Du , H. Wu , Z. Peng , C. Tan , L. Yang , Z. Wang , Mater. Sci. Eng.: R: Rep. 2024, 161, 100839.

[153] J. Zhang , J. Wu , V. M. Le Corre , J. A. Hauch , Y. Zhao , C. J. Brabec , InfoMat 2025, 7, 70005.

[154] Y. Ouyang , C. Zhang , J. Wang , Z. Guo , Z. Wang , M. Dong , Adv. Sci. 2025, 12, 2416259. 10.1002/advs.202416259 PMC12061284 40071782

[155] C. Tan , H. Wu , Z. Lin , L. Yang , G. Hu , Z. Wang , Adv. Funct. Mater. 2025, 07832.

[156] E. Marinho Jr , C. E. Villegas , P. Venezuela , A. R. Rocha , ACS Appl. Energy Mater. 2024, 7, 1051.

[157] Z. Hu , S. Wang , J. Lynch , D. Jariwala , ACS Photonics 2024, 11, 4616.

[158] N. Rono , C. C. Ahia , E. L. Meyer , AIP Adv. 2024, 14, 070702.

